# Isolation and Electronic Structures of Lanthanide(II) Bis(trimethylsilyl)phosphide Complexes

**DOI:** 10.1021/acs.inorgchem.4c02888

**Published:** 2024-09-16

**Authors:** Jack Baldwin, Adam Brookfield, George F. S. Whitehead, Louise S. Natrajan, Eric J. L. McInnes, Meagan S. Oakley, David P. Mills

**Affiliations:** Department of Chemistry, University of Manchester, Oxford Road, Manchester, M13 9PL, U.K.

## Abstract

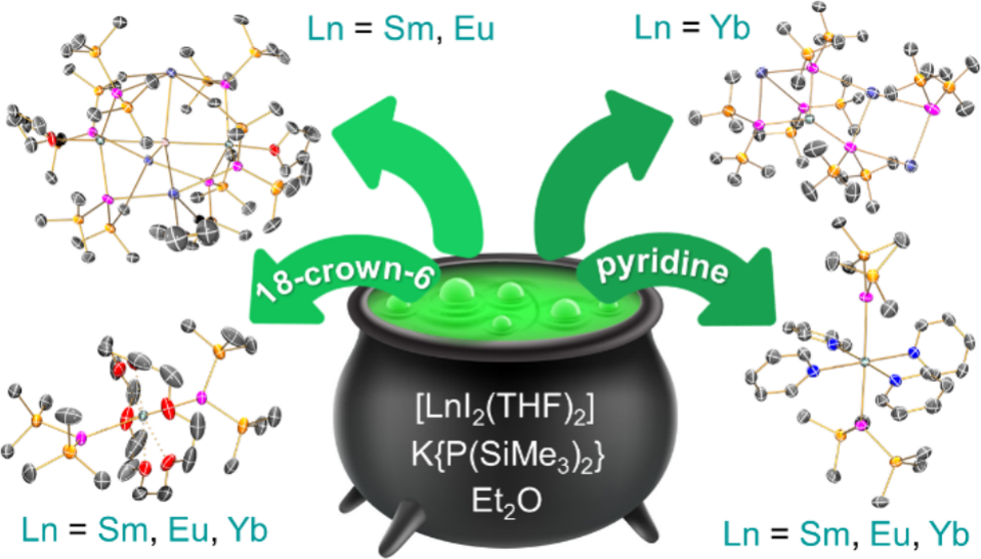

While lanthanide (Ln) silylamide chemistry is mature, the corresponding silylphosphide chemistry is underdeveloped, with [Sm{P(SiMe_3_)_2_}{μ-P(SiMe_3_)_2_}_3_Sm(THF)_3_] being the sole example of a structurally authenticated Ln(II) silylphosphide complex. Here, we expand the Ln(II) {P(SiMe_3_)_2_} chemistry through the synthesis and characterization of nine complexes. The dinuclear “ate” salt-occluded complexes [{Ln[P(SiMe_3_)_2_]_3_(THF)}_2_(μ-I)K_3_(THF)] (**1-Ln**; Ln = Sm, Eu) and polymeric “ate” complex [KYb{P(SiMe_3_)_2_}_3_{μ-K[P(SiMe_3_)_2_]}_2_]_∞_ (**2-Yb**) were prepared by the respective salt metathesis reactions of parent [LnI_2_(THF)_2_] (Ln = Sm, Eu, Yb) with 2 or 3 equiv of K{P(SiMe_3_)_2_} in diethyl ether. The separate treatment of these complexes with either pyridine or 18-crown-6 led to the formation of the mononuclear solvated adducts *trans*-[Ln{P(SiMe_3_)_2_}_2_(py)_4_] (**3-Ln**; Ln = Sm, Eu, Yb) and [Ln{P(SiMe_3_)_2_}_2_(18-crown-6)] (**4-Ln**; Ln = Sm, Eu, Yb), with concomitant loss of K{P(SiMe_3_)_2_}. The complexes were characterized by a combination of NMR, electron paramagnetic resonance (EPR), attenuated total reflectance infrared (ATR-IR), electronic absorption and emission spectroscopies, elemental analysis, SQUID magnetometry, and single crystal X-ray diffraction. We find that these complexes contrast with those of related Ln(II) bis(silyl)amide complexes due to differences in ligand donor atom hardness and ligand steric requirements from Ln−P bonds being longer than Ln−N bonds. This leads to higher coordination numbers, shorter luminescence lifetimes, and smaller eas*y*-axis magnetic anisotropy parameters.

## Abstract 2

Nine lanthanide(II) bis(trimethylsilyl)phosphide complexes are synthesized by salt metathesis methods from parent [LnI_2_(THF)_2_] (Ln = Sm, Eu, Yb) and K{P(SiMe_3_)_2_} in the presence of various donor solvents. The electronic structures of these complexes are probed by a variety of experimental and computational methods.

## Introduction

Compounds containing lanthanide (Ln) elements have found multiple applications, due to their inherent magnetic, optical, and catalytic properties.^1^ As Ln-ligand bonding is mainly ionic Ln ions tend to maximize coordination numbers to saturate their coordination spheres;^1−3^ these can be controlled with appropriate ligands to give complexes with favorable properties.^4−9^ Due to the hard Lewis acidic nature of Ln ions, the vast majority of Ln complexes contain ligands with hard Lewis basic light p-block donor atoms.^1^ For the group 15 elements this trend is epitomized by Ln amide chemistry being well-developed,^10−13^ while Ln complexes bound by analogous ligands containing heavier softer pnictogens are comparatively rare.^14−16^

The bis(trimethylsilyl)amide ligand, {N(SiMe_3_)_2_} (N″), has been used extensively by f-block chemists since the trigonal pyramidal Ln(III) complexes [Ln{N(SiMe_3_)_2_}_3_] were first reported over half a century ago.^10,17−19^ Ln(II) N″ chemistry has flourished since [Eu{N(SiMe_3_)_2_}_2_(sol)_2_] (sol = DME or THF) provided the first structurally authenticated Eu(II) amide complexes in 1981;^10,20^ for example, a rare example of an Sc(II) complex, [K(2.2.2-cryptand)][Sc{N(SiMe_3_)_2_}_3_], was reported in 2017.^21^ By contrast, the chemistry of the heavy pnictogen analogue bis(trimethylsilyl)phosphide, {P(SiMe_3_)_2_} (P″), is immature. This discrepancy can be assigned to the ready commercial availability of HN′′ and its s-block metal salts,^22,23^ while alkali metal P″ reagents are typically synthesized from P(SiMe_3_)_3_.^24,25^ This parent phosphine starting material is pyrophoric and relatively expensive unless it is prepared directly from PNa_3_ (generally made *in situ* from red phosphorus and 3 equiv sodium in DME at reflux) and 3 equiv ClSiMe_3_; this reaction is often performed on large scales (*ca*. 1 mol) and therefore needs dedicated procedures and glassware.^26^

To date, there are only a handful of structurally authenticated group 3 and f-block metal P″ complexes, namely [Sc{C(PPh_2_S)_2_}{P(SiMe_3_)_2_}(py)_2_] (py = pyridine),^27^ [Y{P(SiMe_3_)_2_}_2_{μ-P(SiMe_3_)_2_}]_2_,^28,29^ [Ln{P(SiMe_3_)_2_}_3_(THF)_2_] (Ln = Nd, Tm),^30,31^ [An{P(SiMe_3_)_2_}(Cp*)_2_(Cl)] (An = Th, U), (Cp* = C_5_Me_5_),^25^ [An(Tren^DMBS^){P(SiMe_3_)_2_}], and [An(Tren^TIPS^){P(SiMe_3_)_2_}] (An = Th, U; Tren^DMBS^ = N(CH_2_CH_2_NSiMe_2_^*t*^Bu)_3_, Tren^TIPS^ = N(CH_2_CH_2_NSi^*i*^Pr_3_)_3_);^32^ the asymmetric dinuclear Sm(II) complex [Sm{P(SiMe_3_)_2_}{μ-P(SiMe_3_)_2_}_3_Sm(THF)_3_]^33^ is the sole previously reported Ln(II) P″ complex.

Here, we extend Ln(II) P″ chemistry via the synthesis and characterization of nine complexes that vary by the Ln(II) ion and the donor solvent: the “ate” complexes [{Ln[P(SiMe_3_)_2_]_3_(THF)}_2_(μ-I)K_3_(THF)] (**1-Ln**; Ln = Sm, Eu) and [KYb{P(SiMe_3_)_2_}_3_{μ-K[P(SiMe_3_)_2_]}_2_]_∞_ (**2-Yb**), the pyridine adducts *trans*-[Ln{P(SiMe_3_)_2_}_2_(py)_4_] (**3-Ln**; Ln = Sm, Eu, Yb), and the crown ether derivatives [Ln{P(SiMe_3_)_2_}_2_(18-crown-6)] (**4-Ln**; Ln = Sm, Eu, Yb). These complexes were characterized by a combination of NMR, electron paramagnetic resonance (EPR), attenuated total reflectance infrared (ATR-IR), electronic absorption and emission spectroscopies, elemental analysis, SQUID magnetometry, and single crystal X-ray diffraction. We find that the long Ln−P distances and hard−soft metal−ligand mismatch in **1-Ln**, **2-Yb**, **3-Ln**, and **4-Ln** lead to their electronic structures being vastly different from their Ln(II) N″ counterparts.

## Results and Discussion

### Synthesis

**Scheme 1:**
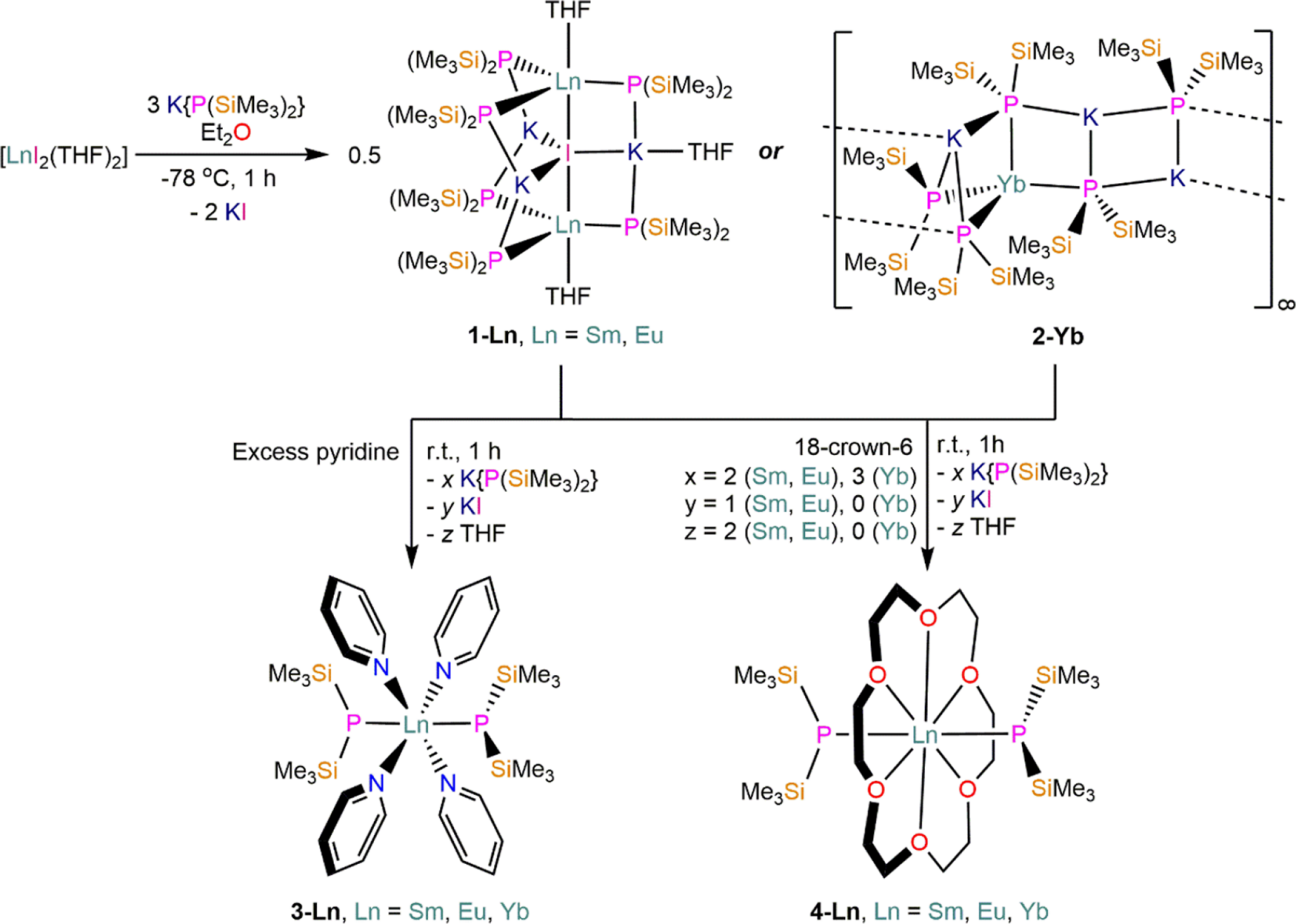
Synthesis of **1-Ln**, **2-Yb**, **3-Ln**, and **4-Ln**

We adapted literature salt metathesis protocols for the synthesis of the dinuclear Sm(II) complex [Sm{P(SiMe_3_)_2_}{μ-P(SiMe_3_)_2_}_3_Sm(THF)_3_] from [SmI_2_(THF)_2_] and 2 equiv K{P(SiMe_3_)_2_} in THF^33^ to prepare **1-Ln** and **2-Yb** from parent [LnI_2_(THF)_2_] (Ln = Sm, Eu, Yb) and 2 or 3 equiv of K{P(SiMe_3_)_2_} in diethyl ether (Scheme 1). Reactions were initiated at −78 °C and reaction mixtures were allowed to stir for 1 h at this temperature before allowing to warm to room temperature briefly, before volatiles were removed *in vacuo* and the products extracted into pentanes. Under these conditions, recrystallization from saturated solutions reproducibly gave poor isolated yields of **1-Ln** (*ca*. 15%); **2-Yb** was only characterized by single crystal X-ray diffraction (XRD) (see below). These “ate” complexes were the only Ln-containing complexes that we were able to identify in the reaction mixtures, however, if either excess pyridine or stoichiometric 18-crown-6 is added before workup then the respective solvated adducts **3-Ln** and **4-Ln** form, accompanied by a concomitant loss of K{P(SiMe_3_)_2_} (Scheme 1). Recrystallization of complexes **3-Ln** and **4-Ln** from saturated toluene solutions gave a wide range of isolated yields of products (from 6−46%), with the Yb(II) analogs tending to give the lowest crystalline yields this may simply be due to differences in ligand: metal size ratios being nonideal for crystal growth for the smaller Yb(II) ions.^34,35^

The bulk compositions of microcrystalline **1-Ln**, **3-Ln**, and **4-Ln** were assessed by a combination of elemental analysis and ATR-IR spectroscopy; we were not able to confidently assign any functional groups using the latter technique (ATR-IR spectra are compiled in the SI Figures S1−S11). The powder XRD pattern of the microcrystalline sample that contained crystals of **2-Yb** showed that a mixture of compounds was present (see SI Figure S17). As we were not able to isolate a pure sample of **2-Yb** we limited our discussion of analytical data obtained for this complex to single crystal XRD. We found that for **4-Ln** the elemental analysis results were in excellent agreement with predicted values, but for **1-Ln** the carbon values obtained were reproducibly lower than expected; we attribute this observation to incomplete combustion arising from carbide formation, which is a common feature for highly air- and moisture-sensitive complexes.^36,37^ For **3-Ln** the hydrogen and nitrogen values obtained are consistent with the loss of one bound pyridine upon drying samples *in vacuo*, with low carbon values again assigned to carbide formation; the low carbon and hydrogen values observed in elemental analysis results of [Yb(PPh_2_)_2_(THF)_4_] was previously assigned to the partial loss of THF *in vacuo*.^38^ The ATR-IR spectra of **1-Ln**, **3-Ln**, and **4-Ln** show largely overlapping absorption bands for each separate family, indicating that the bulk samples show similar features in the solid state.

### X-ray Crystallography

**Figure 1:**
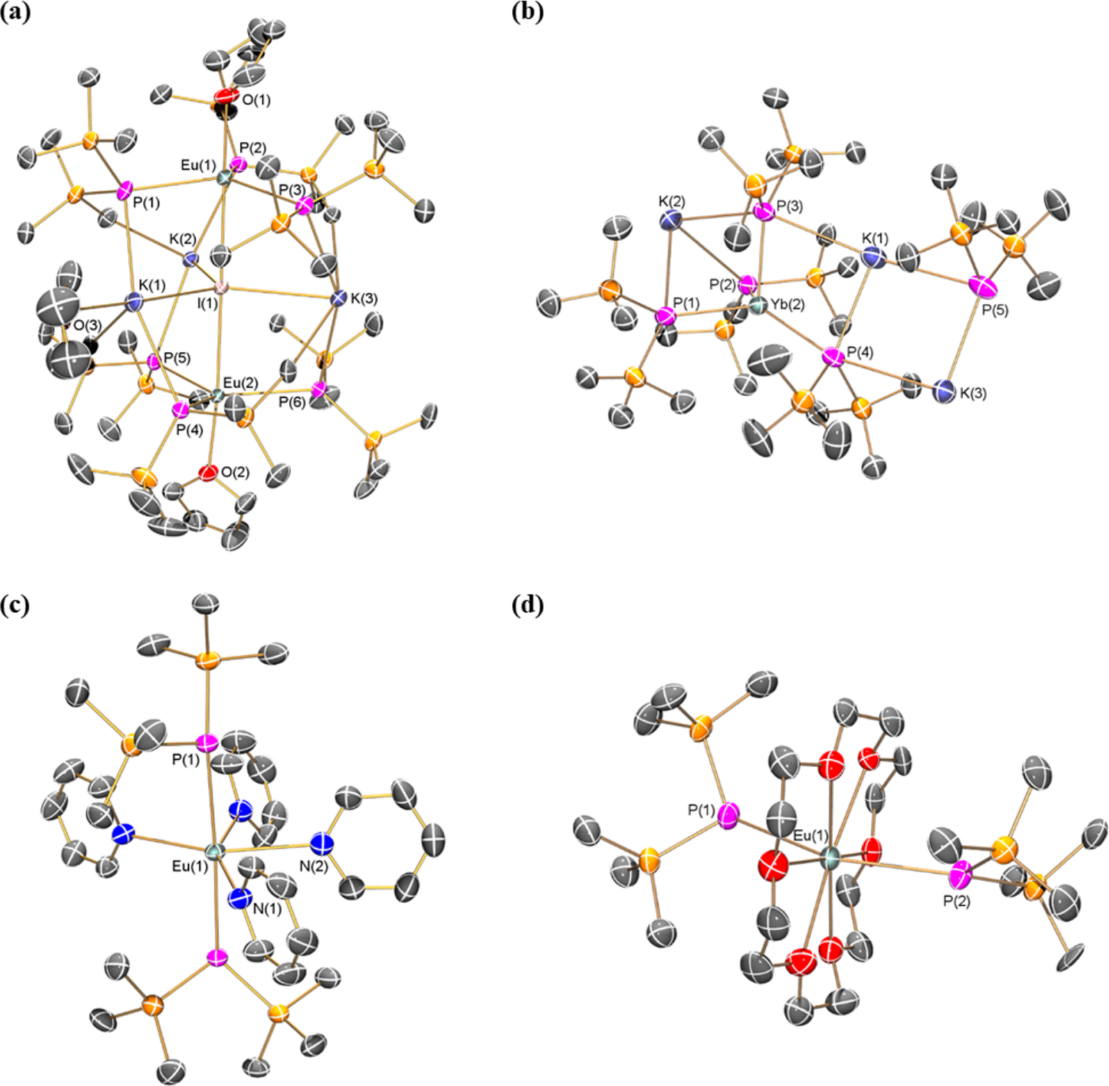
Solid-state structures of (a) **1-Eu** and (b) a single repeating unit of polymeric **2-Yb**, (c) **3-Eu**, and (d) **4-Eu** with selected atomic labeling; Ln = cyan, P = magenta, Si = yellow, I = pink, K = navy blue, O = red, *N* = blue, C = gray. Displacement ellipsoids are set at a 50% probability level; hydrogen atoms are omitted for clarity.

The solid-state structures of **1-Ln**, **2-Yb**, **3-Ln**, and **4-Ln** were determined by single crystal XRD (see Figure 1 for depictions of **1-Eu**, **2-Yb**, **3-Eu**, and **4-Eu**; as other congeners of **1-Ln**, **3-Ln**, and **4-Ln** are isostructural they are depicted in the SI Figures S12−S16). Selected bond distances and angles for all complexes are presented in Tables 1−3, and crystallographic parameters are compiled in SI Tables S1−S3.

The dinuclear solvated Ln(II) “ate” complexes **1-Ln** (Figure 1a and Table 1) can be formally viewed as salt-occluded complexes, with two {KLn(P″)_3_(THF)} fragments trapping KI; an alternative description of the cluster is of a dicationic {IK_3_(THF)}^2+^ moiety sandwiched between two {Ln(P″)_3_(THF)}^−^ anions. Both complexes contain two Ln ions with distorted trigonal bipyramidal geometries, supported by τ_5_ analysis (**1-Sm**: Sm(1) = 0.8460, and Sm(2) = 0.8740; **1-Eu**: Eu(1) = 0.8595 and Eu(2) = 0.8205, see SI Table S4),^39^ with each metal center coordinated by three equatorial phosphides, one axial THF, and one axial iodide. The single iodide bridges the two Ln ions in a near-linear arrangement (Ln−I−Ln = 178.08(2)°, **1-Sm**; 177.93(2)°, **1-Eu**) and also exhibits a distorted trigonal bipyramidal geometry, with three equatorial potassium cations completing its coordination sphere; the Ln centers lie 0.298(2) Å (**1-Sm**) and 0.296(2) Å (**1-Eu**) above the P_3_ plane such that they are closer to the capping THF molecules. The potassium cations each act as a bridge between two phosphides from opposing {Ln(P″)_3_}^−^ fragments to provide the cage structure, and their coordination spheres are completed by multiple electrostatic interactions with methyl groups of P″ ligands; one potassium cation is also bound by THF to reduce the overall symmetry of the cluster. The mean Ln−P distances in **1-Ln** (Ln = Sm, 3.033(7) Å; Eu, 3.038(7) Å) are shorter than the six bridging Sm−P″ bonds (range = 3.100(3)−3.178(3) Å) in [Sm{P(SiMe_3_)_2_}{μ-P(SiMe_3_)_2_}_3_Sm(THF)_3_], but are statistically equivalent with the sole terminal Sm−P″ bond (3.027(3) Å) in the literature complex.^33^ In contrast, the Ln−O distances in **1-Ln** (Ln = Sm, 2.489(7) Å; Eu, 2.496(6) Å), which fall within the range of known Sm−O_THF_ and Eu−O_THF_ bond lengths (2.284(13)−2.951(3) Å),^40^ are far shorter than the mean Sm−O bond length in [Sm{P(SiMe_3_)_2_}{μ-P(SiMe_3_)_2_}_3_Sm(THF)_3_] (2.593(13) Å);^33^ we attribute this to **1-Ln** having more electronically and sterically unsaturated Ln centers.

The solvent-free Yb(II) “ate” complex **2-Yb** (Figure 1b and Table 1) is a one-dimensional (1D) coordination polymer in the solid state that is held together by electrostatic interactions between Yb and K cations, and P″ anions. The structure can be viewed as a block copolymer with alternating {KYb(P′′)_3_} and {K_2_(P″)_2_} units, or alternatively as {K_2_Yb(P″)_4_} fragments bridged by {K(P″)}. The structure is highly disordered, and as both parts of the model show the same connectivity, we discuss the metrical parameters of only the major component for brevity. The Yb centers are each bound by four phosphides and exhibit heavily distorted tetrahedral geometries (P−Yb−P angles: 93.79(13)°, 98.4(2)°, 98.5(2)°, 105.94(14)°, 126.55(14)°, and 125.8(2)°), supported by a τ_4_ parameter of 0.7633,^41^ with the K cations of the {K_2_Yb(P″)_4_} fragments bridging between either two or three coordinated phosphides. The Yb−P distances in the **2-Yb** range between 2.837(7)−3.043(3) Å and the mean Yb−P bond length (2.952(10) Å) is shorter than the corresponding Ln−P distances for **1-Ln**, as expected from consideration of the smaller seven-coordinate ionic radii of Yb(II) (1.08 Å) vs. Sm(II) (1.22 Å) and Eu (1.20 Å).^42^ The K cations are bound by either three or four P atoms, and as with **1-Ln** their coordination spheres are completed by multiple electrostatic interactions with methyl groups of bridging P″ ligands; the phosphides show a range of coordination modes, including μ_2_-Yb,K, μ_3_-K_3_, and μ_3_-K_2_Yb. The structures of **1-Ln** and **2-Yb** differ markedly from distorted tetrahedral Ln(II) *bis*-N″ THF solvates [Ln{N(SiMe_3_)_2_}_2_(THF)_2_] (Ln = Sm, Eu, Yb)^20^ and trigonal planar Ln(II) *tris*-N″ “ate” complexes [Ln(N′′)(μ-N′′)_2_M] (M = Li, Na, K; Ln = Sm, Eu, Yb).^43−45^ These Ln(II) N″ literature complexes are all mononuclear, as a result of their Ln−N bonds being shorter and stronger than the Ln−P bonds in **1-Ln** and **2-Yb**, coupled with the numerous stabilizing Ln···Si−C_γ_ electrostatic interactions and interligand London dipole interactions that are hallmarks of low-coordinate Ln N″ chemistry.^10^

**Table 1:** Selected bond lengths and angles for **1-Ln** and **2-Yb**

parameter	1-Sm	1-Eu	2-Yb
mean Ln−P/Å	3.033(5)	3.0316(4)	2.952(10)
Ln−O/Å	2.489(7)	2.496(6)	-
RangeK−P/Å	3.319(3)−3.407(2)	3.308(2)−3.429(2)	3.143(7)−3.612(8)
Ln(1)−I(1)−Ln(2)/°	178.08(2)	177.93(2)	-
P(1)−Ln(1)−P(2)/°	121.86(5)	118.42(4)	-
P(2)−Ln(1)−P(3)/°	113.93(5)	116.42(4)	-
P(3)−Ln(1)−P(1)/°	121.29(5)	122.37(4)	-
P(4)−Ln(2)−P(5)/°	116.62(4)	121.72(4)	-
P(5)−Ln(2)−P(6)/°	118.19(5)	121.01(4)	-
P(6)−Ln(2)−P(4)/°	122.20(4)	114.46(4)	-
P(1)−Yb(1)−P(5)**/°**	-	-	125.8(2)
P(5)−Yb(1)−P(2)**/°**	-	-	105.94(2)
P(2)−Yb(1)−P(1)**/°**	-	-	98.4(2)
P(5)−Yb(1)−P(3)**/°**	-	-	126.6(2)
P(3)−Yb(1)−P(2)**/°**	-	-	93.8(2)
P(3)−Yb(1)−P(1)**/°**	-	-	98.5(2)

The pyridine-solvated Ln(II) complexes **3-Ln** (Figure 1c and Table 2) crystallize in two different polymorphs, with **3-Sm** and **3-Eu** in the *C*_2_/*c* space group and **3-Yb** in *P*2_1_/*c*; this discrepancy leads to there being one unique molecule in the unit cells of the former and two in the latter. Complexes **3-Ln** all exhibit distorted octahedral geometries and *trans*-configurations, with two phosphides and four equatorially bound pyridines; distortion parameters of 4.200 (**3-Sm**), 4.029 (**3-Eu**), and 0.530 (**3-Yb**) were determined via SHAPE analysis (see SI Table S5).^39,46^ This coordination motif is analogous to several previously reported solvated Ln(II) *bis*-phosphide complexes, e.g., *trans*-[Yb(PPh_2_)_2_(THF)_4_],^38^*trans*-[Ln(PPh_2_)_2_(*N*-MeIm)_4_] (Ln = Sm, Eu, Yb)^38,47^ (*N*-MeIm = *N*-methylimidazole), *trans*-[Ln{P(Mes)_2_}_2_(THF)_4_] (Ln = Sm, Yb),^48,49^ and *trans*-[Sm(κ^1^-*P*-dibenzophospholyl)_2_(THF)_4_].^50^ Complexes **3-Sm** and **3-Eu** are more distorted from ideal octahedral geometries than **3-Yb**, with the early Ln exhibiting three N−Ln−N angles between 74.87(12) and 77.75(14)° and a fourth of 133.3(2)° for **3-Sm** and 131.81(12)° for **3-Eu**; in contrast for **3-Yb** the range of N−Ln−N angles is much narrower (86.16(8)−92.09(7)°). This can be attributed to the increased size of the metal coordination sphere for the larger Ln(II) ions allowing two Me groups from opposing P″ ligands to form additional electrostatic interactions in a mutually *trans*-fashion, with Ln···C distances of 3.809(5) Å (**3-Sm**) and 3.840(4) Å (**3-Eu**); as a consequence the P atoms in these complexes show a greater degree of pyramidalization than in **3-Yb**.

Despite the significant deviation from ideal octahedral geometries in **3-Sm** and **3-Eu** the Ln ions are situated within the N_4_ plane and the P−Ln−P angles do not deviate far from linearity (**3-Sm**: 177.32(4)°; **3-Eu**: 176.65(3)°), in common with both molecules of **3-Yb** (P−Ln−P: 177.69(2)° and 178.09(2)°). The mean Ln−P distances (**3-Sm**: 3.0341(10) Å, **3-Eu**: 3.0363(8) Å, **3-Yb**: 2.924(2) Å) and a range of Ln−N bond lengths (**3-Sm**: 2.698(4)−2.714(4) Å, **3-Eu**: 2.688(3)−2.711 Å, **3-Yb**: 2.501(3)−2.547(3) Å) of **3-Ln** are perhaps best compared with the corresponding metrics of the distorted octahedral imidazole-substituted Ln(II) *bis*-phosphide complexes *trans*-[Ln(PPh_2_)_2_(*N*-MeIm)_4_] (Sm−P: 3.139(2) Å, Eu−P: 3.127(3) Å, Yb−P: 3.0277(7) Å; mean Sm−N: 2.620(2) Å, Sm−N: 2.609(2) Å, Yb−N: 2.491(4) Å).^38^ The shorter Ln−P and longer Ln−N distances in **3-Ln** compared to their respective literature complexes can be attributed to the stronger donor properties of *N*-MeIm vs. pyridine, where the second N atom increases the Lewis basicity of the imidazole. It is noteworthy that the P−Ln−P angles in *trans*-[Ln(PPh_2_)_2_(*N*-MeIm)_4_] are linear as a consequence of the longer Ln−P bond lengths reducing steric buttressing in the Ln coordination spheres, and this is also reflected in smaller deviations of the N−Ln−N angles in the literature complexes (Ln = Sm, 85.4(2)−94.6(2)°; Ln = Eu, 85.5(3)−94.5(3)°; Ln = Yb, 85.61(7)−94.39(7)°).^38^

Complexes **4-Ln** exhibit distorted hexagonal bipyramidal geometries, with mutually *trans*-phosphides and the hexadentate 18-crown-6 coordinated about the equatorial girdle; both **4-Sm** and **4-Eu** have two independent molecules in the unit cell, so the metrical parameters of only one of these is discussed for brevity (Figure 1d and Table 3). The increased Ln coordination number and presence of a puckered macrocyclic ligand in **4-Ln** leads to correspondingly longer Ln−P distances in **4-Ln** (Ln = Sm, 3.089(3) Å; Eu, 3.086(6) Å; Yb: 2.9662(11) Å) and increased pyramidalization of P centers compared with **1-Ln**; complexes **4-Ln** also exhibit a wide range of relatively long Ln−O distances (Ln = Sm, 2.654(7)−2.773(7) Å; Eu, 2.643(8)−2.781(11) Å; Yb, 2.585(4)−2.665(3) Å). The P−Ln−P angle for **4-Yb** (173.98(4)°) is not linear like *trans*-[Yb(PPh_2_)_2_(THF)_4_] (180.0(8)°)^38,47^ and *trans*-[Yb(PPh_2_)_2_(*N*-MeIm)_4_]^38^ (180.0(4)°), which is likely a consequence of the puckered crown ether in **4-Yb**. This deviation from linearity is also seen to a greater extent in **4-Sm** (161.65(7)°) and **4-Eu** (154.80(11)°) due to the larger Ln(II) metal centers having a less saturated coordination sphere than Yb(II). The bulk structural features of **4-Ln** are comparable with *trans*-[Yb(PPh_2_)_2_(THF)_4_],^38^*trans*-[Ln{P(Mes)_2_}_2_(THF)_4_]^48,49^ (Ln = Sm, Yb),^48^ and *trans*-[Sm(κ^1^-*P*-dibenzophospholyl)_2_(THF)_4_],^50^ which also have equatorial O-donor ligands but due to lower Ln coordination numbers exhibit shorter mean Ln−P and Ln−O distances for the corresponding Ln, e.g., *trans*-[Sm{P(Mes)_2_}_2_(THF)_4_] (mean Sm−P: 3.034(2) Å; mean Sm−O: 2.564(5) Å),^48^*trans*-[Yb(PPh_2_)_2_(THF)_4_] (mean Yb−P: 2.911(2) Å; mean Yb−O: 2.434(6) Å).^38^

**Table 2:** Selected Bond Lengths and Angles for **3-Ln**

parameter	3-Sm	3-Eu	3-Yb
Ln−P/Å	3.0342(9)	3.0364(7)	2.928(13)
range Ln−N/Å	2.698(4)−2.715(4)	2.688(3)−2.711(3)	2.501(3)−2.544(2)
Ln···C(1)/Å	3.809(5)	-	-
Ln···C(2)/Å	-	3.840(4)	-
P(1)−Ln−P(1A)/°	177.32(4)	176.65(3)	177.69(2)
N(1)−Ln−N(1A)/°	133.3(2)	131.8(2)	-
N(2)−Ln−N(2A)/°	77.1(2)	77.8(2)	-
N(1)−Ln−N(2A)/°	74.9(2)	75.29(9)	-
N(1A)−Ln−N(2)/°	74.9(2)	75.29(9)	-
N(1)-Ln-N(2)/°	-	-	86.89(7)
N(2)-Ln-N(3)/°	-	-	91.78(7)
N(3)-Ln-N(4)/°	-	-	91.60(7)
N(1)-Ln-N(4)/°	-	-	89.73(7)
angles about P/°	104.20(6)	104.11(5)	103.28(6)
	106.28(4)	106.77(3)	124.57(5)
	134.18(5)	133.64(4)	127.96(5)
∑ angles about P/°	344.66(9)	344.52(7)	355.81(9)

**Table 3:** Selected Bond Lengths and Angles for **4-Ln**

parameter	4-Sm	4-Eu	4-Yb
Ln−P/Å	3.089(3)	3.096(4)	2.9662(10)
range Ln−O/Å	2.654(7)−2.773(7)	2.657(9)−2.781(11)	2.585(5)−2.665(4)
P(1)−Ln−P(2)/°	161.65(7)	154.8(2)	-
P(1)−Yb-P(1a)/°	-	-	173.98(4)
angles about P/°	101.74(19)	101.1(3)	103.51(6)
	120.73(16)	122.0(3)	121.97(5)
	131.17(16)	131.1(3)	124.84(7)
∑ angles about P/°	353.7(3)	354.2(5)	350.32(10)

### Solution NMR Spectroscopy

^1^H, ^13^C{^1^H}, ^29^Si DEPT90, and ^31^P{^1^H} NMR spectra were collected for crystalline samples of **1-Ln**, **3-Ln**, and **4-Ln** and the mixture containing **2-Yb**; ^171^Yb{^1^H} NMR spectra were also obtained for diamagnetic **3-Yb** and **4-Yb** (see SI Figures S18−S37 for NMR spectra of all complexes; selected NMR data are compiled in Table 4). The solid-state structures of **1-Ln** do not appear to be fully maintained in C_6_D_6_ solutions, with three broad signals observed in the ^1^H NMR spectra of each complex due to a combination of paramagnetic broadening and dynamic aggregation processes. These resonances did not give reliable integrations for silyl groups or the α- and β-H of THF and so these spectra could not be interpreted; similarly, no signals arising from **1-Ln** could be assigned in their ^13^C{^1^H}, ^29^Si DEPT90, and ^31^P{^1^H} NMR spectra.

Although **2-Yb** is diamagnetic, this complex was obtained as a mixture together with other Yb(II)-containing products; thus, the NMR spectra could also not be fully assigned. At 298 K the ^1^H NMR spectrum of a *d*_8_-toluene solution of **2-Yb** showed the presence of THF, indicating that THF-solvated Yb or K complexes were present in this mixture, thus the broad resonance at 0.65 ppm that is likely due to the SiMe_3_ groups could not be reliably integrated. Similarly, multiple signals were observed in the ^31^P{^1^H} NMR spectrum of this mixture at 298 K, with only a signal at −236.9 ppm that could be assigned to K{P(SiMe_3_)_2_} with reasonable confidence by comparison with an authentic sample. We do not observe a signal with well-resolved ^1^*J*_PYb_ coupling for unambiguous characterization of ^171^Yb-bound ^31^P nuclei, though the broad signal at δ_P_ = −219.0 ppm is the predominant feature by approximate integration and is likely a Yb(II)-containing complex. We investigated the dynamic behavior of this mixture by performing variable-temperature ^1^H and ^31^P{^1^H} NMR experiments between 213−323 K; surprisingly we were not able to observe signals that could be assigned to **2-Yb** in the ^13^C{^1^H}, ^29^Si DEPT90 and ^171^Yb{^1^H} NMR spectra, which we attribute to rapid aggregation processes. We found that upon cooling the mixture to 213 K the signals in the ^1^H NMR spectra tend to broaden and shift but no additional signals were resolved with heating or cooling. The corresponding ^31^P{^1^H} NMR spectra of this mixture show that upon cooling the major signal at δ_P_ = −219.0 ppm broadens further and two new signals form at *ca*. −211 and −243 ppm; at 213 K the original signal is still seen and is less broad than at 298 K but again no ^171^Yb satellites could be reliably assigned. The ^31^P{^1^H} NMR spectrum of this mixture at 323 K could also not be fully interpreted, with temperature-dependent changes in intensity and chemical shifts of resonances, which are in accord with the presence of a mixture of products and complex solution equilibria.

Samples of **3-Sm** and **3-Eu** that had been dried under dynamic vacuum are only sparingly in C_6_D_6_, likely due to the loss of some coordinated pyridine *in vacuo* (see above), thus solutions for NMR spectroscopy were prepared by adding several drops of pyridine to separate C_6_D_6_ suspensions of **3-Sm** and **3-Eu**. Solutions of **4-Sm** and **4-Eu** in C_6_D_6_ were also prepared, and all four complexes were studied by ^1^H, ^13^C{^1^H}, and ^29^Si DEPT90, and ^31^P{^1^H} NMR spectroscopy. Due to paramagnetic broadening, which was more pronounced for the 4f^7^ Eu(II) complexes, only the ^1^H NMR spectra of **3-Sm** and **4-Sm** could be tentatively assigned. The resonances corresponding to the silyl groups were seen at δ_H_ = 1.40 (FWHM = 50 Hz) for **3-Sm** and δ_H_ = 2.70 ppm for **4-Sm**. Three broad signals were also seen for the *ortho*-, *meta*- and *para*-pyridine protons of **3-Sm**, but these could not be reliably integrated as pyridine was in excess and should be subject to dynamic coordination equilibria. Similarly, for **4-Sm** a signal corresponding to 18-crown-6 was observed (δ_H_ = −1.45 ppm), together with some unbound 18-crown-6 (3.20 ppm) and [K{P(SiMe_3_)_2_}(18-crown-6)] (0.76 ppm), in accord with dynamic processes occurring in solution.

**Figure 2:**
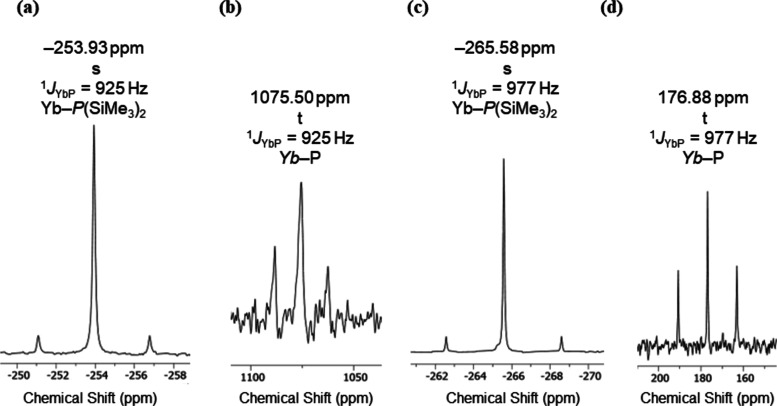
^31^P{^1^H} NMR (162 MHz) and ^171^Yb{^1^H} NMR (71 MHz) spectra of **3-Yb** in C_6_D_6_/pyridine (a, b) and **4-Yb** in C_6_D_6_ (c, d).

All multinuclear NMR spectra of C_6_D_6_ solutions of the diamagnetic Yb(II) complexes **3-Yb** and **4-Yb** could be fully assigned, with the caveat that several drops of pyridine had to be added to solubilize **3-Yb**, which precluded reliable integration of resonances in the ^1^H NMR spectrum (Table 4). The ^1^H and ^13^C{^1^H} NMR spectra of **3-Yb** and **4-Yb** were essentially unremarkable, save that the molecular symmetry in solution is higher than in the solid state and that in the ^13^C{^1^H} NMR spectra the silyl group resonances are virtual triplets due to splitting by strongly coupled 100% abundant *I* = ^1^/_2_^31^P nuclei (δ_C_ = 7.85 ppm, ^2^*J*_PC_ = 5.4 Hz for **3-Yb**; δ_C_ = 8.43 ppm, ^2^*J*_PC_ = 5.6 Hz for **4-Yb**). Similar second-order effects were also seen in the ^29^Si DEPT90 NMR spectra of **3-Yb** (δ_Si_ = 1.58 ppm, ^1^*J*_PSi_ = 16 Hz) and **4-Yb** (δ_Si_ = 1.98 ppm, ^1^*J*_PSi_ = 17 Hz), with these signals also split into virtual triplets from coupling with the strongly coupled ^31^P nuclei. The ^31^P{^1^H} NMR spectra of **3-Yb** and **4-Yb** exhibit resonances at −253.93 and −265.58 ppm, respectively, with satellites to 14.3% abundant *I* = ^1^/_2_^171^Yb nuclei allowing the determination of ^1^*J*_YbP_ coupling constants (925 Hz for **3-Yb** and 977 Hz for **4-Yb**) (Figure 2); a trace amount of K{P(SiMe_3_)_2_} was also observed in the sample of **3-Yb**. The ^31^P chemical shifts of **3-Yb** and **4-Yb** are downfield to those previously observed for [Y{P(SiMe_3_)_2_}_2_{μ-P(SiMe_3_)_2_}]_2_ (δ_P_: − 104.8 ppm, ^1^*J*_YP_ = 122.4 Hz; − 107.8 ppm, ^1^*J*_YP_ = 56.7 Hz),^29^ while their ^1^*J*_YbP_ coupling constants are greater than those reported for *trans*-[Yb(PPh_2_)_2_(THF)_4_] (δ_P_ = −3.0 ppm, ^1^*J*_YbP_ = 840 Hz).^38^ The ^171^Yb{^1^H} NMR spectra of **3-Yb** and **4-Yb** show the expected triplet resonances at 1075.50 and 176.88 ppm, respectively, vs. a [Yb(Cp*)_2_(THF)_2_] external reference^51^ from the respective coupling of ^171^Yb nuclei with two equivalent ^31^P nuclei; the ^1^*J*_YbP_ coupling constants extracted were identical to the respective ^31^P{^1^H} NMR experiments (Figure 2). These resonances can be compared with the doublet of doublets resonance observed for the Yb(II) phosphide complex [Yb{P[CH(SiMe_3_)_2_](C_6_H_3_-2-OMe-3-Me)}_2_(THF)_2_] (δ_Yb_ = 663.6 ppm, ^1^*J*_YbP_ = 603 and 767 Hz).^52^ It has been shown previously that ^171^Yb{^1^H} NMR chemical shifts can vary significantly with coordinated solvent for the Yb(II) aryloxide complexes [Yb(OAr′)_2_(L)_*n*_] (Ar′= C_6_H_2_Bu_2_^t^-2,6-Me-4; (L)_*n*_ = (OEt_2_)_2_, (THF)_2_, (THF)_3_, (py)_2_, Me_2_PCH_2_CH_2_PMe_2_) and [Yb(μ-OAr′)(X)]_2_ (X = OR′, {N(SiMe_3_)_2_}), e.g., [Yb(OAr′)_2_(THF)_2_] δ_Yb_ = 345.0 ppm and [Yb(OAr′)_2_(py)_2_] δ_Yb_ = 745.0 ppm.^53^

**Table 4:** ^1^H, ^13^C{^1^H}, ^29^Si DEPT90, ^31^P{^1^H} and ^171^Yb{^1^H} NMR Chemical Shifts for **3-Yb** and **4-Yb** in C_6_D_6_

complex	^1^H (δ)	^13^C{^1^H} (δ)	^29^Si DEPT90 (δ)	^31^P{^1^H} (δ)	^171^Yb{^1^H} (δ)
**3-Yb**	0.30 (s, 36H, C*H*_3_)	7.85 (Vir. t, *C*H_3_, ^2^*J*_PC_ = 5.4 Hz)	1.58 (Vir. t, *Si*Me_3_, ^1^*J*_Psi_ = 16 Hz)	−253.93 (s, Yb-*P*, ^1^*J*_YbP_ = 925 Hz)	1075.50 (t, *Yb*-P, ^1^*J*_YbP_ = 925 Hz)
	6.65 (m, br, *m*−C*H*)	124.14 (*m*-*C*H)			
	6.95 (m, br, *p*−C*H*)	137.46 (*p*-*C*H)			
	8.69(m, br, *o*−C*H*)	151.45 (*o*-*C*H)			
**4-Yb**	0.56 (s, 36H, C*H*_3_)	8.43 (Vir. t, *C*H_3_, ^2^*J*_PC_ = 5.6 Hz)	1.94 (Vir. t, *Si*Me_3_, ^1^*J*_Psi_ = 17 Hz)	−265.58 (s, Yb-*P*, ^1^*J*_YbP_ = 977 Hz)	176.88 (t, *Yb*-P, ^1^*J*_YbP_ = 977 Hz)
	3.43 (s, 24H, {-C_2_*H*_4_O-}_6_)	69.06 ({-*C*_2_H_4_O-}_6_)			

### UV−vis−NIR Spectroscopy

**Figure 3:**
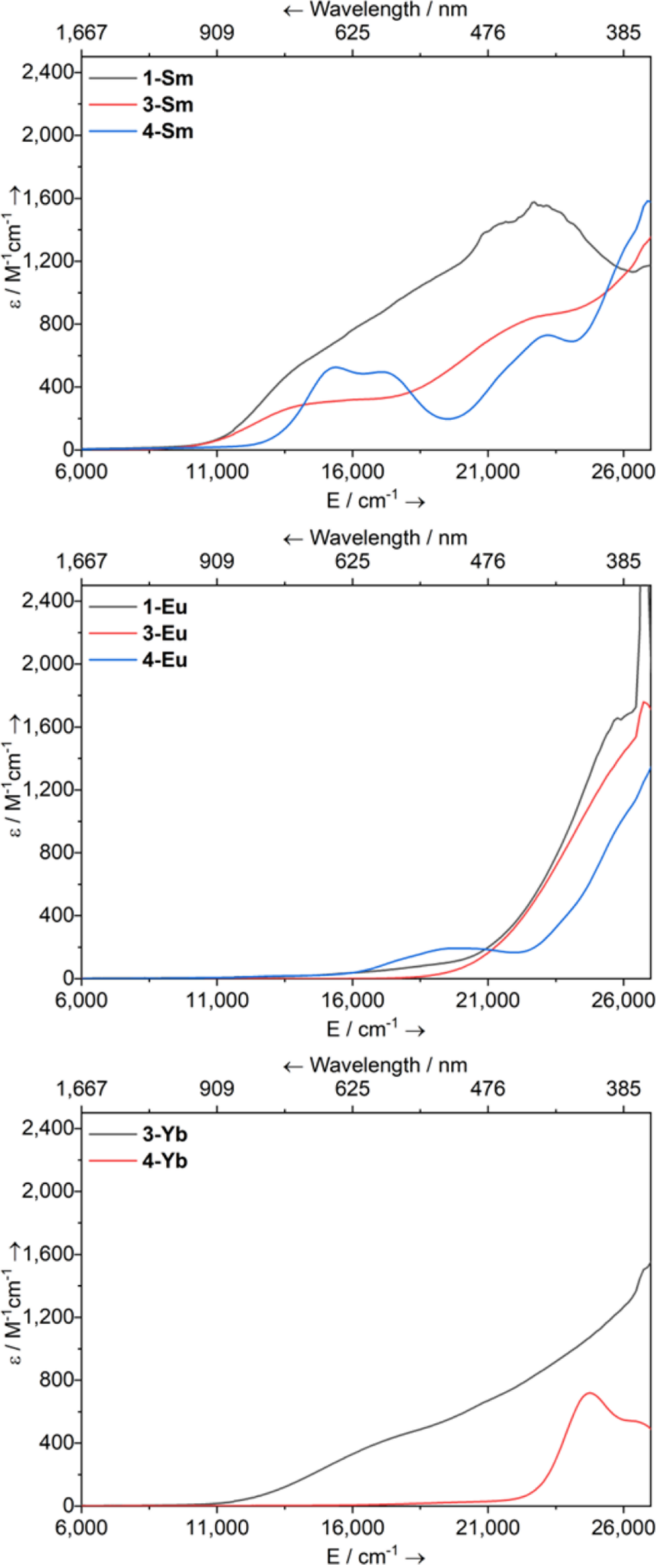
Overlaid electronic UV−vis−NIR absorption spectra of **1-Ln**, **3-Ln**, and **4-Ln** (2 mM in toluene) between 6000 and 27,000 cm^−1^ (1667−370 nm).

As the electronic absorption features of Ln(II) complexes are sensitive to both the identity of the Ln(II) ion and the coordination environment,^1^ the electronic transitions of 2 mM toluene solutions of crystalline samples of **1-Ln**, **3-Ln**, and **4-Ln** were studied by UV/vis/NIR absorption spectroscopy (see Figure 3 for compiled spectra; individual spectra are available in the SI Figures S38−S48). Within the visible region for Ln(II) complexes, strong broad absorptions are observed as a result of the formal spin- and Laporte-allowed f−d transitions from the stabilization of the 5d orbitals (*c.f*. Ln(III) complexes). The intraconfigurational f−f transitions, which are typically Laporte-forbidden are often not observed for Ln(II) complexes due to the intense, broad f−d features.^54,55^

It is known that the Sm(II) ion gives rise to complexes that exhibit intense colors as a result of the multiple spin- and orbital-allowed f−d transitions within the visible region.^1^ Toluene solutions of **1-Sm** are dark green and display a broad and intense absorption feature containing multiple shoulders, which span across the visible region (Figure 3), with λ_max_ = 22,730 cm^−1^ (440 nm, ε = 1575 M^−1^ cm^−1^). This complex has the highest absorption coefficient among the family of complexes reported herein, due to there being twice as many Sm(II) ions in the sample. The broad absorption band in the visible region likely arises from 4f^5^5d^1^ ← 4f^6^ transitions, due to its similarity with absorption features previously assigned for [Sm{N(Si^i^Pr_3_)_2_}_2_] by comparison with its emission spectrum (rather than intraconfigurational f−f transitions that are expected to be much sharper and higher in energy).^56^ Toluene solutions of mononuclear **3-Sm** and **4-Sm** are also dark green and exhibit broad intense absorptions similar to those of **1-Sm**, with these bands also spanning the visible and UV regions. Complex **3-Sm** shows two absorption maxima at λ_max_ = 22,530 cm^−1^ (444 nm, ε = 833 M^−1^ cm^−1^) and λ_max_ = 13,950 cm^−1^ (717 nm, ε = 278 M^−1^ cm^−1^), while **4-Sm** has three absorption maxima at λ_max_ = 23,150 cm^−1^ (432 nm, ε = 730 M^−1^ cm^−1^), λ_max_ = 17,360 cm^−1^ (576 nm, ε = 488 M^−1^ cm^−1^), and λ_max_ = 15,390 cm^−1^ (650 nm, ε = 526 M^−1^ cm^−1^); the most intense f−d transition for all three Sm(II) P″ complexes occurs at similar energies. These values can be compared to complexes such as [Sm{N(SiMe_3_)_2_}_2_]_2_ [λ_max_, cm^−1^ (nm) = 28,600 (350), 24,000 (416), 18,000 (556), and 16,200 (618)],^57^ [Sm{N(Si^*i*^Pr_3_)_2_}_2_] [λ_max_, cm^−1^ (nm) = 29,900 (335), 23,500 (426), and 17,200 (582)],^58^ and [Sm(Cp*)_2_] (Cp* = C_5_Me_5_) [λ_max_, cm^−1^ (nm) = 16,694 (599)]^59^ in toluene, which exhibit similar features to those displayed by **1-Sm**, **3-Sm**, and **4-Sm**.

Eu(II) complexes are usually much paler in color than their Sm(II) homologues as the Laporte-allowed f−d transitions in a given ligand environment tend to occur at higher energy and lie in the UV spectral region, particularly in aqueous solutions and when complexed by macrocyclic ligand such as 2,2,2-cryptand. In addition, weaker f−f transitions may not be observed.^56^ This generality holds for **1-Eu** (pale green/yellow) and **3-Eu** (pale orange), which are essentially featureless apart from bands that tail into the visible region that are similar to those observed for their Sm(II) congeners. By contrast, **4-Eu** (pale yellow) additionally exhibits a low-intensity broad absorption with λ_max_ = 19,610 cm^−1^ (510 nm, ε = 192 M^−1^ cm^−1^), which may be due to the coordinated 18-crown-6 imposing a strong equatorial ligand field that red-shifts some f−d transitions into the visible region. Reports of the electronic absorption spectra of Eu(II) phosphide complexes are rare, but a tetrahydrofuran solution of *trans*-[Eu(PPh_2_)_2_(*N*-MeIm)_4_] was reported to display absorptions in the UV region at 46,730 cm^−1^ (214 nm, ε = 18,000 M^−1^ cm^−1^), 40,820 cm^−1^ (245 nm, ε = 6200 M^−1^ cm^−1^), and 37,450 cm^−1^ (267 nm, ε = 3000 M^−1^ cm^−1^).^60^ The electronic absorption spectra of the Eu(II) P″ complexes herein can also be compared to those of related Eu(II) silylamide complexes such as [Eu{N(Si^*i*^Pr_3_)_2_}_2_] [λ_max_, cm^−1^ (nm) = 33,300 (300) and 30,200 (331)]^58^ and [K(2.2.2-cryptand)][Eu{N(Si^*t*^BuMe_2_)_2_}_3_] [λ_max_, cm^−1^ (nm) = 33,300 (300)].^61^

For Yb(II) complexes, no f−f transitions are possible, due to their 4f^14^ closed-shell electronic structures. However, the crystal field imposed is able to strongly influence the colors of Yb(II) complexes by varying the energies of the f−d transitions.^1^ Toluene solutions of **3-Yb** are dark green and exhibit a band tailing into the visible region from UV, possessing a shoulder at approximately λ_max_ = 17,200 cm^−1^ (581 nm, ε = 416 M^−1^ cm^−1^). Finally, pale yellow toluene solutions of **4-Yb** show an absorption band at λ_max_ = 24,750 cm^−1^ (404 nm, ε = 720 M^−1^ cm^−1^) tailing in from the UV region though, compared to **3-Yb**, this absorption is weak. There is a paucity of UV−vis data for Yb(II)-phosphide systems within the literature, but [Yb{(μ-P^*t*^Bu_2_)_2_Li(THF)}_2_] exhibits a set of absorption bands [λ_max_, cm^−1^ (nm) = 45,000 (222), 37,000 (270), 33,300 (300), and 27,500 (363)], with no features reported at lower energies.^62^ As with the Eu(II) P″ complexes above, the electronic absorption spectra of **3-Yb** and **4-Yb** can also be compared to those of related Yb(II) silylamide complexes, e.g., [Yb{N(Si^*i*^Pr_3_)_2_}_2_] [λ_max_, cm^−1^ (nm) = 31,000 (323), 28,200 (355), 24,600 (406), and 20,400 (491)] and [K(2.2.2-cryptand)][Yb{N(Si^*t*^BuMe_2_)_2_}_3_] [λ_max_, cm^−1^ (nm) = 30,750 (325), 28,550 (350), and 21,050 (475)].^58,61^

### Photoluminescence Studies

**Figure 4:**
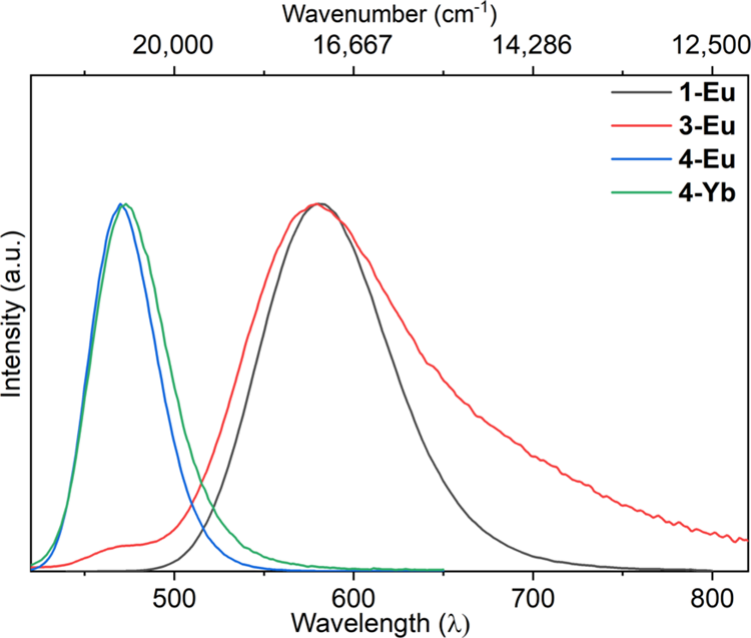
Emission spectra of toluene solutions of **1-Eu** (0.508 mM, excited at 31,750 cm^−1^, 315 nm; emission at 17,270 cm^−1^, 579 nm), **3-Eu** (0.111 mM, excited at 34,480 cm^−1^, 290 nm; emission at 17,270 cm^−1^, 579 nm), **4-Eu** (1.600 mM, excited at 24,690 cm^−1^, 405 nm; emission at 21,050 cm^−1^, 475 nm), and **4-Yb** (1.580 mM, excited at 27,030 cm^−1^, 370 nm; emission at 21,050 cm^−1^, 475 nm); and spectrum of **3-Yb** (0.177 mM, excited at 28,570 cm^−1^, 350 nm; emission at 15,040 cm^−1^, 665 nm) omitted as it showed very weak emission.

The optical properties of toluene solutions of crystalline samples of **1-Eu**, **3-Ln** (Ln = Eu, Yb), and **4-Ln** (Ln = Eu, Yb) at room temperature were studied by photoluminescence spectroscopy (see Figure 4, and SI Figures S49−S54 for individual spectra). The Sm(II) analogs of these complexes were not studied as the intense absorption features spanning the visible region fully quenches emission from f−d charge transfer electronic transitions through nonradiative decay. The intra-4f luminescent transitions of Ln(III) complexes are well-understood and do not vary significantly with the ligand field due to the radially contracted 4f orbitals.^63^ By contrast, the energies of the f−d luminescence transitions of Ln(II) complexes tend to vary to a much greater extent and are more vibrationally broadened. Here, the local crystal field and solvent stabilization effects in the excited state, in combination with the relatively more stabilized d-orbitals, enable other competitive nonradiative relaxation pathways.^64,65^

We observed vibrationally broadened emission bands for all solutions studied, which are characteristic of f−d transitions;^66^ the luminescence profile of **3-Eu** is asymmetric and very broad, spanning most of the visible spectrum. Following excitation between 24,390−50,000 cm^−1^ (200−410 nm) for **1-Eu** and **3-Eu** the emission maxima occur in the green region (λ_em_ = 18,020 cm^−1^, 555 nm) of the electromagnetic spectrum and for **4-Eu** and **4-Yb** these are in the blue region (λ_em_ = 21,050 cm^−1^, 475 nm); the spectra of **3-Yb** are very weak and could not be interrogated further. As the emission spectra observed do not vary with changes in excitation wavelength, it can be inferred that each complex has one excited state in common. These emission bands are best compared with those seen for the Ln(II) complexes [K(2.2.2-cryptand)][Ln{N(Si^*t*^BuMe_2_)_2_}_3_] (Ln = Eu, λ_em_ = 18,520 cm^−1^, 540 nm, green; Ln = Yb, λ_em_ = 15,380 cm^−1^, 650 nm, red) and [Eu{N(Si^*i*^Pr_3_)_2_}_2_] (λ_em_ = 16,950 cm^−1^, 590 nm, yellow-orange).^58,61^ The excitation spectra of all complexes studied are asymmetric and exhibit some features that match those in the electronic absorption spectra with higher energy maxima than literature complexes (**1-Eu**: λ = 31,750 cm^−1^, 315 nm; **3-Eu**: λ = 34,480 cm^−1^, 290 nm; **4-Eu** and **4-Yb**: λ = 24,690 cm^−1^, 405 nm). The THF-solvated Eu(II) complex **1-Eu** and the pyridine-solvated complex **3-Eu** exhibit larger Stokes shifts between their excitation and emission spectra and emit at lower energies than **4-Eu** and **4-Yb**. This suggests that the f−d charge transfer bands are sensitive to small changes in the local coordination environment. The excitation spectra additionally exhibit vibrationally broadened bands at lower energies and no higher energy electronic excitations are observed that are similar to those seen for [K(2.2.2.cryptand)][Ln{N(Si^*t*^BuMe_2_)_2_}_3_], where two features are clearly resolved (Ln = Eu, 30,300 and 35,710 cm^−1^, 280 and 330 nm; Yb, 27,780 and 31,250 cm^−1^, 320 and 360 nm).^61^ The data obtained for all complexes are in accord with emission being due to the initial population of a higher energy charge transfer state followed by the deactivation of an f−d excited state.^67^

The luminescence lifetimes of **1-Eu** and **3-Eu** recorded at the respective emission maxima following 26,670 cm^−1^ (375 nm) excitation were fitted satisfactorily to biexponential decay models, whereas **4-Eu** was modeled with a monoexponential decay function (see SI Figures S55−S58). This indicates the presence of two emissive excited states in solution on the nanosecond time scale for **1-Eu** and **3-Eu** and a single- or time-averaged emitting state for **4-Eu**. Significant variation in the luminescence lifetimes across the series is observed; **1-Eu** (τ_1_ = 811(±270) ns; τ_2_ = 1760(±34) ns), **3-Eu** (τ_1_ = 42(±0.5) ns; τ_2_ = 771(±10.40) ns) and **4-Eu** (τ_1_ = 447(±1.2) ns) (Table 5). The lifetimes for **1-Eu** and **3-Eu** were fitted to a biexponential decay, indicating that there are two emissive states for these complexes, whereas **4-Eu** exhibited monoexponential decay kinetics. The origin of the larger error on the lifetimes for **1-Eu** is not immediately obvious, but this is possibly due to the second emissive component being much longer-lived than in the other complexes, leading to a larger error when fitting the longer tail of the kinetic trace. These lifetimes are all significantly shorter than those seen for the Eu(II) silylamide complexes [Eu{N(Si^*i*^Pr_3_)_2_}_2_] (49.6(±1.3) μs)^68^ and [K(2.2.2-cryptand)][Eu{N(Si^*t*^BuMe_2_)_2_}_3_] (5.8(±0.005) μs),^61^ and are of the order of those previously reported for macrocyclic Eu(II) complexes,^69^ such as [Eu(benzo-15-crown-5)_2_][ClO_4_]_2_ (0.14 μs in MeOH)^70^ and [Eu(benzo-18-crown-6)_2_][Cl]_2_ (0.028 μs).^71^ As expected from the energy gap law, the luminescence lifetimes of the Yb(II) complex **4-Yb** is shorter than the Eu(II) congener **4-Eu**, due to additional competitive nonradiative quenching processes and was also modeled as a biexponential decay.^72^

**Table 5:** Summary of λ_ex_ (nm), λ_em_ (nm), and τ (ns) for Complexes **1-Eu**, **3-Eu**, **4-Eu**, and **4-Yb**

complex	λ_ex_ (nm)	λ_em_ (nm)	τ_1_ (ns)	error (ns)	% cont.	τ_2_ (ns)	error (ns)	% cont.
**1-Eu**	375	579	811	270	17	1760	34	83
**3-Eu**	375	579	42	0.50	19	771	10.40	81
**4-Eu**	375	475	447	1.20	100			
**4-Yb**	375	475	16	0.04	47	348	3.54	53

### Magnetism

The magnetic susceptibility for powdered samples of crystalline **1-Ln**, **3-Ln**, and **4-Ln** (Ln = Sm, Eu) suspended in eicosane were studied by variable-temperature DC SQUID magnetometry as well as Complete Active Space Self-Consistent Field Spin−Orbit (CASSCF-SO) calculations (Selected parameters compiled in Table 6, see SI Figures S59−S70 for all magnetic data). In general, there is reasonable agreement between the measured and calculated susceptibility values as well as those for the respective free ion values (Sm(II) 4f^6 7^F_0_; Eu(II) 4f^7 8^S_7/2_) for all complexes.

The calculated values for **1-Sm**, **3-Sm**, and **4-Sm** are slightly higher than predicted due to the thermal population of the lowest-lying ^7^F_J_ excited states; the shapes of the calculated curves are accurately reproduced. At most, the CASSCF-calculated χ_M_*T* values for **1-Sm**, **3-Sm**, and **4-Sm** differ from the measured values by 0.74 cm^3^ mol^−1^ K. Complex **1-Sm** exhibits a χ_M_*T* value of 3.09 cm^3^ mol^−1^ K at 300 K, which is in close agreement with the free ion value for two Sm(II) ions.

**Table 6:** Product of the Molar Susceptibility and Temperature, χ_M_*T* (cm^3^ mol^−1^ K), of **1-Sm**, **1-Eu**, **3-Sm**, **3-Eu**, **4-Sm**, and **4-Eu**, at 2 and 300 K as Determined by SQUID Magnetometry on Powder Samples, CASSCF Calculations, and Free Ion Values for Monomeric Ions

	SQUID magnetometry		CASSCF calculations		free ion^1^
complex	1.8 K	300 K	2 K	300 K	χ_M_*T*
**1-Sm**	0.04	3.09	0.05	3.57	1.45
**1-Eu**	14.38	17.35	15.71	15.71	7.88
**3-Sm**	0.02	1.37	0.15	2.11	1.45
**3-Eu**	7.20	9.51	7.90	7.90	7.88
**4-Sm**	0.01	1.44	0.02	1.79	1.45
**4-Eu**	1.30	7.68	7.85	7.86	7.88

Complexes **1-Eu**, **3-Eu**, and **4-Eu** all exhibit only minor changes in χ_M_*T* as the temperature is decreased, which is characteristic of spin-only systems, and the χ_M_*T* values are consistent with Eu(II), 4f^7^. However, there are small inflections in χ_M_*T* at low temperatures for all of the Eu(II) complexes, which we attribute to poor thermal equilibration of the sample; this observation for bimetallic **1-Eu** could also be due to a magnetic interaction between the two Eu(II) ions. For **1-Eu** and **3-Eu**, the experimental values lie approximately 1.6 cm^3^ K mol^−1^ above that predicted by CASSCF, whereas there are closer agreements with the experimental and calculated values for **4-Eu**, which differ from those predicted by 0.18 cm^3^ K mol^−1^. Analogously to **1-Sm**, complex **1-Eu** exhibits a χ_M_*T* value of 17.35 cm^3^ mol^−1^ K at 300 K, which is in close agreement with the free ion value for two Eu(II) ions; the low-temperature magnetization data are also consistent with spin-only ions.

### EPR Spectroscopy

**Figure 5:**
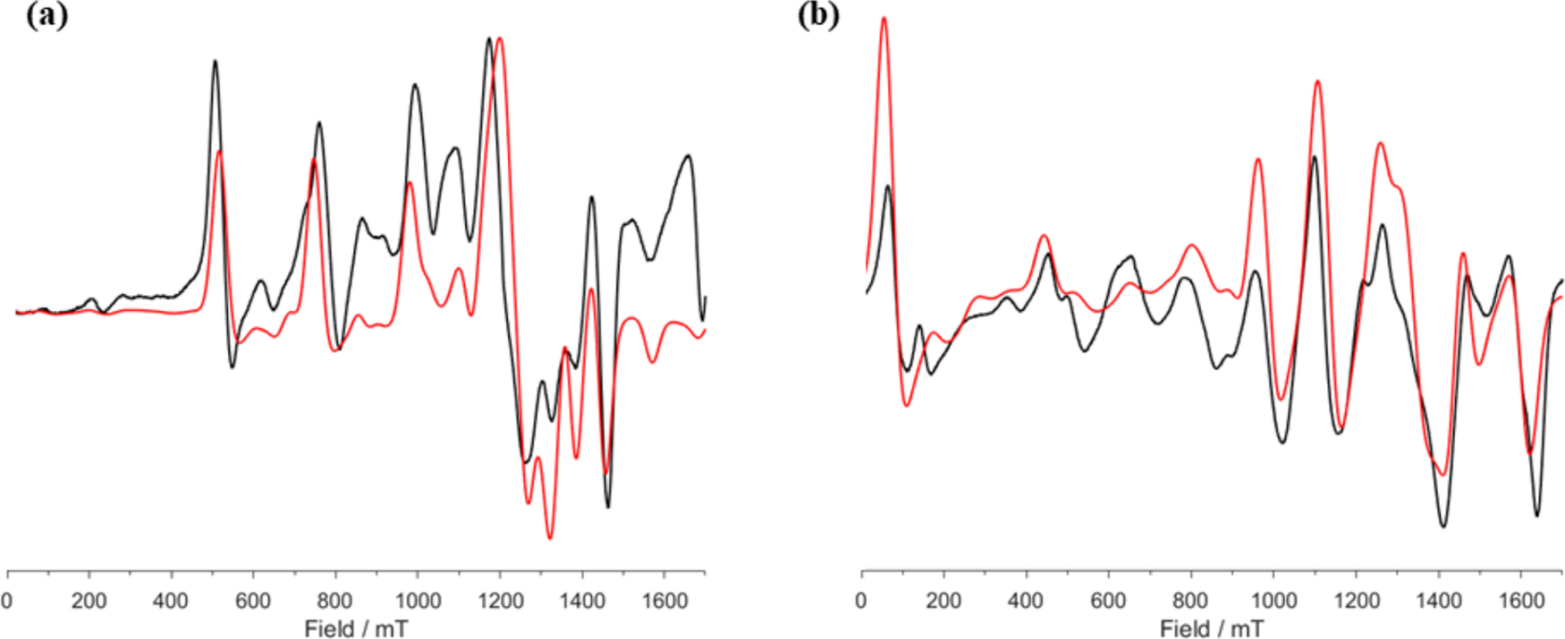
Experimental (black trace) and simulated (red trace) Q-band powder EPR spectra for (a) **3-Eu** and (b) **4-Eu** at 5 K.

Because Eu(II) is a 4f^7^, spin-only ion (*S* = 7/2) it is relatively straightforward to study by EPR spectroscopy, and there is a substantial literature, although nowhere near as much as for the isoelectronic Gd(III) ion. This literature mostly addresses solid-state chemistry, and phosphor materials in particular, but several molecular Eu(II) systems have been studied.^58,73,74^ The electronic structures of crystalline samples of the Eu(II) complexes **1-Eu**, **3-Eu** and **4-Eu** were investigated by c.w. X-band (*ca*. 9.4 GHz) and Q-band (*ca*. 34 GHz) EPR spectroscopy (see Figure 5 and SI Figures S71−S125). The spectra of powder samples are well-resolved, while those from frozen solution (9:1 toluene/hexane, 10 mM) are less so. This is presumably due to discrepancies between solid-state and solution structures (see above) as well as strain effects on the solution structure and hence the spin Hamiltonian parameters. Hence, we focus our discussion on solid-state spectra. The spectra for the monometallic complexes were simulated, using EasySpin,^75^ with the simple Zeeman and ZFS spin Hamiltonian:
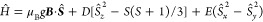
where *S* is the electron spin quantum number, μ_B_ is the Bohr magneton, ***B*** is the applied magnetic field, *g* is the electronic *g*-value (treated as isotropic), and *D* and *E* are the axial and rhombic components, respectively, of the ZFS interaction matrix. Higher-order ZFS terms, which are possible for *S* = 7/2, were neglected.

Powder samples of **3-Eu** at the Q-band and low temperature give spectra with intense resonances ranging from *ca*. 0.5 T (with weaker features at lower-field) to the maximum range of the electromagnet (1.7 T). In the wings of the spectrum, a regular progression of transitions with separation *ca*. 2 kG is observed (with more closely spaced transitions in the middle); modeling shows these to be associated with the principal (*z*) axis of the ZFS interaction (see below), hence corresponding to an axial ZFS parameter (*D*) of magnitude *ca*. 1 kG in field units or 3 GHz (for *g* = 2). Upon cooling the sample of **3-Eu** from 50 to 5 K, there is a marked increase in intensity of the lower field of these transitions, consistent with a negative *D*. Powder samples of **4-Eu** gave well-resolved spectra that spread over a wider field range than for **3-Eu**, hence **4-Eu** has a larger ZFS. Again, a clear regular progression with separations of *ca*. 2 kG is observed, but these are due to transitions in the *xy* plane of a near-axial spectrum, corresponding to a dominant axial ZFS term of magnitude *ca*. 6 GHz.

Good simulations were obtained for **3-Eu** with *D* = −3.3 GHz and a rhombicity parameter |*E*/*D*| = 0.19 (defined with axial and rhombic limits of *E*/*D* = 0 and 1/3, respectively), with *g* = 2.0. In order to reasonably match the relative intensities of the dominant (at least at low temperature) low-field resonances, it was necessary to assume a slight preferential ordering of the system along the molecular *z*-axis in the magnetic field (as opposed to a true powder average); this is consistent with a negative *D* (hence eas*y*-axis magnetic anisotropy). For **4-Eu**, good simulations were achieved with *D* = −5.4 GHz and a rhombicity parameter |*E*/*D*| = 0.06, again with *g* = 2.0. In contrast to **4-Eu**, it was not necessary to introduce an ordering parameter.

The sign of *D* is the same, being negative for both **3-Eu** and **4-Eu**; |*D*| is larger for **4-Eu** but the ZFS is much nearer the axial limit. Negative *D* parameters have also been found for the near-linear *bis*-amide Eu(II) complexes [Eu{N(SiMePh_2_)_2_}_2_]^73^ and [Eu{N(Si^i^Pr_3_)_2_}_2_]^58^ (*D* = −13.8 and −17.3 GHz, respectively). These observations are consistent with ZFS being dominated by the axial phosphide ligands in **3-Eu** and **4-Eu**. Both **3-Eu** and **4-Eu** have a substantially smaller |*D*| than the linear amides above, presumably a function of the softer ligands. The reason for the larger |*D*| for **4-Eu** than for **3-Eu** is less obvious: the P−Eu−P angle in **4-Eu** is further from linear geometry (154.80(11)−161.19(9)° *cf*. 176.65(3)° for **3-Eu**) and the Eu−P bonds are longer (3.075(3)−3.099(3) Å) than for **3-Eu** (3.0364(7) Å). One possible explanation may come from the superposition model for S-state ions, in which the ZFS is modeled as a sum of contributions from individual nuclei in the ligand sphere.^76^ A recent study of several Gd(III) complexes found the contribution to the ZFS from N-donors to be greater than that of O-donors; the leading terms are negative and along the M−L bonds.^77^ In **3-Eu** and **4-Eu**, the Eu−O/N bonds are in the equatorial plane (approximating to axial symmetry) hence the effect of these ligands would be a *positive* contribution to the total ZFS. This could result in a smaller |*D*| for **3-Eu**, which has N-donor equatorial ligands. Consistent with this, it was recently reported that *trans-*[Gd(Cl)_2_(py)_4_][BPh_4_] has a smaller |*D*| than *trans-*[Gd(Cl)_2_(THF)_5_][BPh_4_], where the only difference is the equatorial ligands.^78^ The smaller |*E*/*D*| for **4-Eu** is more readily explainable in terms of the smaller distortion from axiality in the molecular *xy* plane: the O−Eu−O angles in the equatorial {EuO_6_} plane of **4-Eu** are all 60.0(3) ± 2°, i.e., very regular, while in the equatorial {EuN_4_} plane of **3-Eu** there are three small N−Eu−N angles of 75.29(9)−77.75(14)° and one large angle of 131.81(12)°. This appears to be more important in determining the rhombicity than the smaller (greater deviation from linearity) P−Eu−P angle found in **4-Eu** than in **3-Eu**.

The EPR spectra of bimetallic **1-Eu** are more complex at low temperatures, and on warming, the intensity rapidly grows in the middle of the spectrum (see SI Figures S71−S88). This is consistent only with an exchange interaction between the two Eu(II) ions. Modeling of low-temperature magnetic data shows that this exchange must be very small (|*J*| < *ca*. 30 GHz), but this puts it in the same regime as the Eu(II) ZFS, hence very complex spectra result and we have as yet been unable to model them satisfactorily.

### Density Functional Theory (DFT) Calculations

The electron densities of **3-Yb** and **4-Yb** were calculated using PBE^79^ to determine the nature of the Yb−P interactions (see SI Figures S129−S131). Frontier molecular orbitals show that there is no substantial orbital overlap between Yb and P, where the lone pair remains localized on P, although a larger P 3p contribution is present in the highest occupied molecular orbital of **3-Yb**. Delocalization indices, δ(Yb−P) and Wiberg bond orders (WBO) report the number of electrons shared between two nuclei; for **4-Yb**, the mean δ(Yb−P) = 0.24 and WBO is 0.28, however, these values are higher for **3-Yb**: δ(Yb−P) = 0.33 and WBO is 0.36. The electron density at the bond critical point ρ_BCP_(Yb−P) shows the Yb−P interaction in **3-Yb** (0.034 au) is stronger than in **4-Yb** (0.015 au). To put these values in a broader context, metal−ligand bonding in the Yb(II) complexes [Yb(Cp*)_2_(THF)]^80^ and [Yb{N(Si^i^Pr_3_)_2_}_2_]^58^ was calculated at the same level of theory using structures from single crystal XRD. For [Yb(Cp*)_2_(THF)] the ten Yb−C interactions were averaged to give a δ(Yb−C) of 0.11, WBO of 0.09, and ρ_BCP_(Yb−C) of 0.027 au; within the same complex, the two Yb−O interactions with THF were averaged and gave a δ(Yb−O) of 0.16, WBO of 0.15, and ρ_BCP_(Yb−O) of 0.031 au.^80^ For [Yb{N(Si^i^Pr_3_)_2_}_2_] the two Yb−N interactions were averaged to give a δ(Yb−N) of 0.33, WBO of 0.27, and ρ_BCP_(Yb−N) of 0.051 au.^58^ The strengths of the Yb−P interactions in **3-Yb** and **4-Yb** are similar to the Yb−N and Yb−O interactions of these literature examples, but the values obtained are highly dependent on ligand substituents, coordination numbers, and the identity of coligands, precluding extended comparisons.

### *Ab Initio* Calculations

The electronic structures of **1-Sm**, **1-Eu**, **3-Sm**, **3-Eu**, **4-Sm**, and **4-Eu** were investigated by CASSCF-SO calculations using the OpenMolcas package (see SI Tables S6−S12 and Figures S126−S128).^81^ For **1-Eu**, two models were calculated with one Eu(II) center replaced by a diamagnetic Sr(II) center in each case. As the coordination environments at each of these positions are different, two sets of effective *g*-values for the lowest Kramers doublet (KD) are obtained (*g*_1_ = 13.91, *g*_2_ = 0.05, *g*_3_ = 0.04; *g*_1_ = 13.59, *g*_2_ = 0.52, *g*_3_ = 0.33) where the largest *g*-value is directed at ca. 44° and *ca*. 83° from the Eu−I−Eu axis, respectively. The ground state |*m*_*s*_⟩ composition of the two magnetic centers in **1-Eu** are also differently mixed, where the first is | ∓ 5/2⟩ (39%), | ∓ 7/2⟩ (31%), | ∓ 3/2⟩ (20%), and | ∓ 1/2⟩ (7%) and the other is | ± 3/2⟩ (39%), | ± 5/2⟩ (36%), | ± 1/2⟩ (13%), and | ± 7/2⟩ (11%). Like its isoelectronic analogue Gd(III), Eu(II) is isotropic, and this high level of mixing is expected with a nonsymmetry-preserving ligand field and a large number of low-lying excited states, as seen in Tables S6 and S7. In addition, the EPR results showed a weak exchange interaction between the two Eu(II) ions on the same order of magnitude as the zero-field splitting, adding further complexity to the electronic structure.

For **3-Eu** and **4-Eu**, the calculated energies of the lowest-lying KDs differ from the experimental ZFS values, especially for **3-Eu**. However, these energy values are very small (the lowest four KDs of **3-Eu** span 0.015 cm^−1^ whereas for **4-Eu** the KDs span 0.619 cm^−1^), which may indicate that the discrepancy is due to the energy scale being outside of the accuracy of the methods used; this has been seen previously in CASSCF-SO studies of the [GdPc]^−^ complex.^82^ For **3-Eu**, a mixed ground state KD is observed, consisting of | ± 7/2⟩ (44%), | ± 5/2⟩ (29%), | ± 3/2⟩ (16%), and | ± 1/2⟩ (7%) with effective g-values *g*_1_ = 13.70, *g*_2_ = 0.41, and *g*_3_ = 0.27, with the largest *g*-value directed at *ca*. 34° from the P−Eu−P axis. Complex **4-Eu** also exhibits a mixed ground state consisting of | ± 7/2⟩ (33%), | ± 3/2⟩ (16%), | ± 5/2⟩ (13%), | ± 1/2⟩ (12%), | ∓ 1/2⟩ (11%), | ∓ 5/2⟩ (9%), and | ∓ 3/2⟩ (5%) with *g*_1_ = 11.11, *g*_2_ = 4.41, and *g*_3_ = 1.64 with the largest *g*-value being approximately perpendicular to the P−Eu−P axis. Both **3-Eu** and **4-Eu** have ground state KDs with |*m*_*s*_⟩ = 7/2 as the largest component, which is what was found experimentally for [Eu{N(SiMePh_2_)_2_}_2_]^73^ and [Eu{N(Si^i^Pr_3_)_2_}_2_].^58^ However, **3-Eu** and **4-Eu** still exhibit highly mixed KDs similar to those of the bimetallic complex **1-Eu**. Conversely, the ground state |*m*_*J*_⟩ compositions for **1-Sm** and **4-Sm** are very pure, consisting of |0⟩ (98%), and |0⟩ (99%), respectively. By contrast, **3-Sm** possesses what appears to be a mixed ground state with |0⟩ (46%).

## Conclusions

Nine Ln(II) bis(trimethylsilyl)phosphide complexes have been synthesized by salt metathesis methodologies from parent LnI_2_ and K{P(SiMe_3_)_2_}, by variation of the Ln(II) ion and donor solvents. While “ate” complexes form as multinuclear aggregates in the presence of THF, the addition of pyridine or 18-crown-6 to reaction mixtures leads to the displacement of THF and excess K{P(SiMe_3_)_2_} to give neutral mononuclear complexes with mutually *trans*-phosphide ligands and equatorially bound pyridine or crown ether. A mixture of compounds was observed in samples of **2-Yb**, which limited characterization of this complex to single crystal XRD, but all other complexes were isolated cleanly to allow for more complete characterization and comparisons with related Ln(II) phosphide and silylamide complexes; experimental data were benchmarked by DFT and *ab initio* calculations as appropriate. The solid-state structures of the Ln(II) P″ complexes showed higher coordination numbers than their N′′ counterparts. This is due to their longer and weaker Ln−P bonds compared to Ln−N bonds leading to coordinative unsaturation, with Ln coordination spheres completed by additional interactions through bridging P′′ or by charge-dense O- and N-donor ligands.

Paramagnetic broadening precluded the full interpretation of multinuclear solution state NMR spectra of the paramagnetic 4f^6^ Sm(II) and 4f^7^ Eu(II) P″ complexes reported herein, but we were able to assign NMR spectra of the diamagnetic 4f^14^ Yb(II) complexes. We extracted ^171^Yb, ^31^P, ^29^Si, ^13^C, and ^1^H chemical shifts, finding that the former was extremely sensitive to the nature of the coligand(s), and we observed relatively large ^1^*J*_YbP_ coupling constants. The electronic absorption spectra of toluene solutions of all Ln(II) P″ complexes herein showed features similar to those previously reported for related Ln(II) silylamide complexes. The Yb(II) and Eu(II) P′′ complexes also exhibited broad luminescence bands, with a significant ancillary ligand-dependent variation of the wavelengths of emission maxima and luminescence lifetimes; these tended to be shorter than those for related Ln(II) silylamide complexes. As expected rich EPR spectra were observed for the *S* = 7/2 Eu(II) P″ complexes due to significant zero-field splitting, whereby small exchange interactions for the bimetallic complex convoluted precluded facile modeling. For the two monometallic Eu(II) complexes, these data show mixed ground states, with *g*_*z*_ axes that vary in direction depending on the ligand environment. The eas*y*-axis magnetic anisotropy parameters were found to be smaller than those for related Eu(II) silylamide complexes due to a combination of weaker Ln−P vs. Ln−N bonds and the identity of ancillary ligands, which also led to large variations between the two P″ complexes.

## Experimental Section

### General Methods

All manipulations were conducted under argon with the strict exclusion of oxygen and water by using a Schlenk line and glovebox techniques. K{P(SiMe_3_)_2_} (KP″)^25^ and LnI_2_(THF)_2_ (Ln = Sm, Eu, Yb) were prepared following the literature procedures.^68^ Diethyl ether and toluene were purged with nitrogen and passed through columns containing the alumina catalyst and molecular sieves, and pyridine was dried over CaH_2_ before being degassed, refilled with argon, and stored over 4 Å molecular sieves (pyridine) or stored over a potassium mirror before use (diethyl ether, toluene). Hexane was dried by refluxing over potassium, stored over a potassium mirror, and then degassed before use. 18-Crown-6 was dissolved in DCM and dried over 4 Å molecular sieves before being filtered and the removal of the solvent *in vacuo*. For NMR spectroscopy, C_6_D_6_ was dried by refluxing over K, and it was vacuum transferred and degassed by three freeze−pump−thaw cycles before use. NMR spectra were recorded on a Bruker AVIII HD 400 spectrometer operating at 400.07 (^1^H), 100.60 (^13^C{^1^H}), 79.48 (^29^Si DEPT90) MHz, referenced to SiMe_4_, and 161.98 (^31^P{^1^H}) MHz, referenced to 85% H_3_PO_4_, and a JEOL JNM-ECZ 400 MHz spectrometer operating at 399.78 (^1^H), 100.52 (^13^C{^1^H}), 79.42 (^29^Si DEPT90) MHz, referenced to SiMe_4_, 161.83 (^31^P{^1^H}) MHz referenced to 85% H_3_PO_4_ and 70.67 (^171^Yb{^1^H}) MHz, referenced to [Yb(Cp*)_2_(THF)_2_]. ATR-IR spectra were recorded on microcrystalline solids by using a Bruker α spectrometer with a Platinum-ATR module. UV−vis−NIR spectroscopy were recorded on a PerkinElmer Lambda 750 spectrometer on 2 mM toluene solutions in a 1 cm path length cuvette and was corrected to a toluene reference cell. Elemental analysis was carried out by Mr Martin Jennings and Mrs Anne Davies at the Microanalytical Service, School of Chemistry, the University of Manchester using a Thermo Scientific Flash Smart Elemental Analyzer with D4001 Flat Base Smooth Wall Tin Capsules (6 × 3 mm).

Single crystals suspended in Fomblin on a Micromount were examined using a Rigaku XtalLAB AFC11 diffractometer equipped with a CCD area detector and graphite-monochromated Cu Kα (λ = 1.54178 Å) or Mo Kα radiation (λ = 0.71073 Å). Intensities were integrated from data recorded on 1° frames by ω rotation. Cell parameters were refined from the observed positions of all strong reflections in each data set. A Gaussian grid face-indexed correction was used to account for X-ray absorption.^85^ The structures were solved using SHELXS;^83^ the data sets were refined by full-matrix least-squares on all unique F 2 values,^84^ with anisotropic displacement parameters for all non-hydrogen atoms, and with constrained riding hydrogen geometries; Uiso(H) was set at 1.2 (1.5 for methyl groups) times Ueq of the parent atom. The largest features in final difference syntheses were close to heavy atoms and were of no chemical significance. CrysAlisPro^85^ was used for control and integration, and SHELX^83,84^ was employed through OLEX2^86^ for structure solution and refinement. ORTEP-3^87^ and POV-Ray^88^ were employed for molecular graphics.

Powder XRD data were obtained on small batches of microcrystalline **2-Yb** that were suspended in Fomblin oil to prevent sample decomposition from oxygen and water. These samples were mounted on a Micromount and placed on a goniometer head under a cryostream to cool the sample to 100 K, freezing the Fomblin to suspend the crystallites for the duration of the experiment. The PXRD data were measured on a Rigaku FR-X diffractometer, operating in powder diffraction mode using Cu Kα radiation (λ = 1.5418 Å) with a Hypix-6000HE detector and an Oxford Cryosystems nitrogen flow gas system. Data were collected between 3 and 70 °θ, with a detector distance of 150 mm and a beam divergence of 1.0 mRad.^89^ X-ray data were collected using CrysAlisPro.^85^ For data processing, the instrument was calibrated using LaB_6_ as the standard. Then, X-ray data were reduced and integrated using CrysAlisPro.^85^

Steady-state and time-resolved emission spectra were recorded at room temperature from 300 to 800 nm in Youngs tap appended 10 mm path length quartz cuvettes on an Edinburgh Instruments FLS-1000 photoluminescence spectrometer equipped with a 450 W steady-state xenon lamp, a 60 W microsecond pulsed xenon flash lamp, and interchangeable picosecond pulsed diode lasers (EPL-375 and EPL-405), with single 325 mm focal length excitation and emission monochromators in the Czerny Turner configuration and a red-sensitive photomultiplier in Peltier (air cooled) 53 housing (Hamamatsu R928P). Lifetime data were recorded following excitation at 375 nm using the EPL-375 diode laser with time correlated single photon counting (TCSPC). Lifetimes were obtained by a tail fit on the data obtained. Plotting, fitting, and analysis of data were carried out using the built-in instrumental software and Origin 2019b. All data were fitted with exponential decay models and the goodness of fit was evaluated by minimization of the residuals squared, χ^2^ and *R*^2^ analysis.

Static variable-temperature magnetic moment data were recorded in an applied DC field of 0.1 T on a Quantum Design MPMS XL7 superconducting quantum interference device (SQUID) by using doubly recrystallized powdered samples. Samples were prepared in an NMR tube containing a finely ground material, with eicosane as a restraint, which was then flame-sealed *in vacuo*. Samples were carefully checked for purity and data reproducibility between several independently prepared batches for each compound examined. Care was taken to ensure complete thermalization of the sample before each data point was measured, and samples were immobilized in an eicosane matrix to prevent sample reorientation during measurements. Diamagnetic corrections were applied using tabulated Pascal constants, and measurements were corrected for the effect of the blank sample holders (flame-sealed Wilmad NMR tube and straw) and the eicosane matrix.

Continuous Wave (CW) X-band (*ca*. 9.4 GHz) spectra were recorded with a Bruker EMX spectrometer fitted with a Super High Q X-band resonator and at Q-band (*ca*. 33.9 GHz) microwave frequency using a Bruker EMX300 spectrometer. Polycrystalline and frozen solution (9:1 toluene/hexane, 10 mM) samples of **1-Eu**, **3-Eu**, and **4-Eu** were sealed in quartz X-band and Q-band EPR tubes *in vacuo*; samples were lightly ground with a mortar and pestle to reduce the amount of sample decomposition, but we note that some effects due to polycrystallinity remain in the spectra below. The presence of a very sharp resonance at *g* = 2.00 is attributed to an impurity in the quartz EPR tubes and serves as an internal reference for comparing relative intensities. The spectra were simulated, using EasySpin 6.0.0-dev.48 using the pepper function.^75^

The OpenMolcas^81^ (version v19.11-d14be45) quantum chemistry package was used to perform CASSCF-SO calculations on **1-Ln**, **3-Sm**, **3-Eu**, **4-Sm**, and **4-Eu** to determine the electronic structures. The molecular geometry from a single crystal XRD structure was used by selecting a single molecule from the asymmetric unit and taking only the largest disorder component. Electron integrals were calculated using basis sets from the ANO-RCC library^90,91^ with VTZP quality on the metal atom, VDZP quality on the P and O atoms and VDZ quality on all other atoms, employing the second-order DKH Hamiltonian for scalar relativistic effects. Resolution of identity Cholesky decomposition (RICD) of the two-electron integrals with atomic compact Cholesky decomposition (acCD) auxiliary basis sets was employed to reduce computational demand.^92^ The molecular orbitals (MOs) for **1-Ln**, **3-Sm**, **3-Eu**, **4-Sm**, and **4-Eu** were optimized using state-averaged CASSCF (SA-CASSCF) wave functions; the active space taken was the valence 4f electrons and seven 4f orbitals. For both **3-Sm** and **4-Sm**, SA-CASSCF calculations were performed for all possible spins and configuration state functions: 7 septets. For **3-Eu** and **4-Eu**, SA-CASSCF calculations were performed for all possible spins and the following number of roots: 1 octet, 48 sextets, 392 quartets, and 560 doublets. A subset of these roots was then mixed by spin–orbit coupling with the restricted active space self-interaction (RASSI) method, where 1 octet, 48 sextets, 119 quartets, and 113 doublets were included. For **1-Sm**, one Sm(II) ion was replaced with Sr(II), a diamagnetic equivalent, to determine the electronic structure and magnetic properties of one spin center at a time. The SA-CASSCF calculations were performed for all possible spins and configuration state functions: 7 septets. For **1-Eu**, one Eu(II) ion was replaced with Sr(II), a diamagnetic equivalent, to determine the electronic structure and magnetic properties of one spin center at a time. The SA-CASSCF wave functions included 1 octet, 48 sextets, 392 quartets, and 560 doublets; a subset of these roots (1 octet, 48 sextets, 119 quartets, 113 doublets) were then mixed by spin–orbit coupling using the RASSI method. The SINGLE_ANISO module was used to decompose the resulting spin−orbit wave functions into the CF Hamiltonian formalism.^93^ Diamond was employed for molecular graphics.^94^

Gaussian 16 Rev C.01^95^ was used to perform density functional theory calculations. Hydrogen positions were optimized while freezing all heavier atoms at the crystal structure positions using the PBE^79^ density functional with the Stuttgart RSC 1997 ECP for Yb^96^ and cc-pDVZ^97^ basis set for the ligand atoms. Dispersion interactions were treated using Grimme’s D3 dispersion correction.^98^ Topological analysis of the total electron density was performed using Multiwfn 3.8,^99^ where the electron density at the bond critical point and delocalization indices were calculated to quantify the Yb−P interaction strength.

#### [{Sm[P(SiMe_3_)_2_]_3_(THF)}_2_(μ-I)K_3_(THF)] (**1-Sm**)

To a precooled (−78 °C) suspension of [SmI_2_(THF)_2_] (0.5484 g, 1 mmol) in diethyl ether (10 mL) was added dropwise a suspension of KP″ (0.6494 g, 3 mmol) in diethyl ether (10 mL). The resultant green reaction mixture was stirred for 1 h at −78 °C before being allowed to warm to room temperature over 20 min. All volatiles were removed *in vacuo*, the green solid was extracted with pentanes (30 mL) and was filtered to yield a dark green solution. The filtrate was concentrated slowly to *ca*. 20 mL and stored at 3 °C to yield dark green crystals, which were isolated and dried *in vacuo* to afford the title compound. Yield = 0.1330 g, 0.0729 mmol, 15%. Anal. calcd (%) for C_48_H_132_IK_3_O_3_P_6_Si_12_Sm: C, 31.58; H, 7.29. Found (%): C, 30.58; H, 7.42. ^1^H NMR (400 MHz, C_6_D_6_, 298 K): δ 0.10 (br, FWHM ≈ 180 Hz), 1.09 (br, FWHM ≈ 280 Hz), 1.78 (br, FWHM ≈ 260 Hz). ^13^C{^1^H} NMR (101 MHz, C_6_D_6_, 298 K), ^29^Si DEPT90 NMR (79 MHz, C_6_D_6_, 298 K), ^31^P{^1^H} NMR (162 MHz, C_6_D_6_, 298 K): Not observed due to paramagnetic broadening. FTIR ν/cm^−1^: 2938 (m), 2881 (m), 1389 (m), 1239 (s), 1027 (m), 808 (s), 625 (s).

#### [{Eu[P(SiMe_3_)_2_]_3_(THF)}_2_(μ-I)K_3_(THF)] (**1-Eu**)

Prepared by the same method as **1-Sm** using [EuI_2_(THF)_2_] (0.5500 g, 1 mmol) and KP″ (0.6494 g, 3 mmol) to give pale yellow/green crystals of **1-Eu**. Yield = 0.1332 g, 0.0728 mmol, 15%. Anal. calcd (%) for C_48_H_132_IK_3_O_3_P_6_Si_12_Eu: C, 31.53; H, 7.28. Found (%): C, 30.78; H, 7.54. ^1^H NMR (400 MHz, C_6_D_6_, 298 K): δ 0.23 (br, FWHM ≈ 40 Hz), 0.30 (br, FWHM ≈ 40 Hz), 0.87 (br, FWHM ≈ 30 Hz), 1.25 (br, FWHM ≈ 50 Hz). ^13^C{^1^H} NMR (101 MHz, C_6_D_6_, 298 K), ^29^Si DEPT90 NMR (79 MHz, C_6_D_6_, 298 K), ^31^P{^1^H} NMR (162 MHz, C_6_D_6_, 298 K): Not observed due to paramagnetic broadening. FTIR ν/cm^−1^: 2934 (m), 2883 (m), 1397 (m), 1235 (s), 1029 (m), 810 (s), 620 (s).

#### [KYb{P(SiMe_3_)_2_}_3_{μ-K[P(SiMe_3_)_2_]}_2_]_∞_ (**2-Yb**)

Prepared by the same method as **1-Sm** using [YbI_2_(THF)_2_] (0.5711 g, 1 mmol) and KP″ (0.6494 g, 3 mmol) to give several pale orange crystals of **2-Yb** within mainly microcrystalline material. As **2-Yb** could not be isolated the analytical data obtained on the mixture of complexes in this sample are provided here. Yield = 0.6014 g. Anal. calcd (%) for C_30_H_90_K_3_P_5_Si_10_Yb: C, 30.61; H, 7.71. Found (%): C, 28.60; H, 7.71. ^1^H NMR (400 MHz, C_6_D_6_, 298 K): δ 0.65 (s, 90H, PSi(C*H*_3_)_3_), 1.56 (br, m, 12H, THF), 4.01 (br, m, 12H, THF). ^13^C{^1^H} NMR (101 MHz, C_6_D_6_, 298 K): No resonances observed due to facile disaggregation. ^29^Si DEPT90 NMR (79 MHz, C_6_D_6_, 298 K): No resonances observed due to facile disaggregation. ^31^P{^1^H} NMR (162 MHz, C_6_D_6_, 298 K): δ −219.0 (s, [KYb{*P*(SiMe_3_)_2_}_3_{μ-K[*P*(SiMe_3_)_2_]}_2_]_∞_).

#### *trans*-[Sm{P(SiMe_3_)_2_}_2_(py)_4_] (**3-Sm**)

To a precooled (−78 °C) suspension of [SmI_2_(THF)_2_] (0.5484 g, 1 mmol) in diethyl ether (10 mL), a suspension of KP″ (0.4329 g, 2 mmol) in diethyl ether (10 mL) was added dropwise. The resultant green reaction mixture was stirred for 1 h at −78 °C before being allowed to warm to room temperature over 20 min. All volatiles were removed *in vacuo*, and the green solid was extracted with pentanes (30 mL). Pyridine (∼ few drops) was added and the resultant dark green solution was filtered. The filtrate was concentrated to *ca*. 20 mL and stored at 3 °C to yield dark green crystals, which were isolated and dried *in vacuo* to afford the title compound. Yield = 0.1786 g, 0.2174 mmol, 22%. Anal. calcd (%) for C_27_H_51_N_3_P_2_Si_4_Sm ([Sm{P(SiMe_3_)_2_}_2_(py)_3_], loss of py *in vacuo*): C, 43.68; H, 6.92; N, 5.66. Found (%): C, 40.87; H, 6.47; N, 5.45. ^1^H NMR (400 MHz, C_6_D_6_, 298 K): δ 1.40 (br, PSi(C*H*_3_)_3_, FWHM ≈ 50 Hz), 2.37 (br, pyridine, FWHM ≈ 120 Hz), 3.99 (br, pyridine, FWHM ≈ 140 Hz), 4.96 (br, pyridine, FWHM ≈ 120 Hz). ^13^C{^1^H} NMR (101 MHz, C_6_D_6_, 298 K), ^29^Si DEPT90 NMR (79 MHz, C_6_D_6_, 298 K), ^31^P{^1^H} NMR (162 MHz, C_6_D_6_, 298 K): Not observed due to paramagnetic broadening. FTIR ν/cm^−1^: 2941 (m), 2879 (m), 1595 (m), 1441 (m), 1235 (m), 1032 (m), 820 (s), 697 (s), 615 (s).

#### *trans*-[Eu{P(SiMe_3_)_2_}_2_(py)_4_] (**3-Eu**)

Prepared by the same method as **3-Sm** using [EuI_2_(THF)_2_] (0.5500 g, 1 mmol) and KP″ (0.4329 g, 2 mmol) to give orange crystals of **3-Eu**. Yield = 0.2145 g, 0.2606 mmol, 26%. Anal. calcd (%) for C_27_H_51_N_3_P_2_Si_4_Eu ([Eu{P(SiMe_3_)_2_}_2_(py)_3_], loss of py *in vacuo*): C, 43.59; H, 6.91; N, 5.65. Found (%): C, 44.39; H, 6.79; N, 6.50. ^1^H NMR (400 MHz, C_6_D_6_, 298 K), ^13^C{^1^H} NMR (101 MHz, C_6_D_6_, 298 K), ^29^Si DEPT90 NMR (79 MHz, C_6_D_6_, 298 K), ^31^P{^1^H} NMR (162 MHz, C_6_D_6_, 298 K): Not observed due to paramagnetic broadening. FTIR ν/cm^−1^: 2941 (m), 2879 (m), 1595 (m), 1439 (m), 1239 (m), 1034 (m), 802 (s), 701 (s), 619 (s).

#### *trans*-[Yb{P(SiMe_3_)_2_}_2_(py)_4_] (**3-Yb**)

Prepared by the same method as **3-Sm** using [YbI_2_(THF)_2_] (0.5711 g, 1 mmol) and KP″ (0.4329 g, 2 mmol) to give dark green crystals of **3-Yb**. Yield = 0.0506 g, 0.0599 mmol, 6%. Anal. calcd (%) for C_27_H_51_N_3_P_2_Si_4_Yb ([Yb{P(SiMe_3_)_2_}_2_(py)_3_], loss of py *in vacuo*): C, 42.39; H, 6.72; N, 5.49. Found (%): C, 36.97; H, 6.32; N, 5.23. ^1^H NMR (400 MHz, C_6_D_6_, 298 K): δ 0.30 (s, 36H, PSi(C*H*_3_)_3_), 6.65 (br, m, *meta*−C*H*), 6.95 (br, m, *para*−C*H*), 8.69 (br, m, *ortho*−C*H*). ^13^C{^1^H} NMR (101 MHz, C_6_D_6_, 298 K): δ 7.85 (Vir. t, PSi(*C*H_3_)_3_, ^2^*J*_PC_ = 5.4 Hz), 124.14 (*meta*-*C*H), 137.46 (*para*-*C*H), 151.45 (*ortho*-*C*H). ^29^Si DEPT90 NMR (79 MHz, C_6_D_6_, 298 K): δ 1.58 (Vir. t, *Si*(CH_3_)_3_, ^1^*J*_PSi_ = 16 Hz). ^31^P{^1^H} NMR (162 MHz, C_6_D_6_, 298 K): δ −253.93 (s, *P*-Yb, ^1^*J*_YbP_ = 925 Hz).^171^Yb{^1^H} NMR (70.67 MHz, C_6_D_6_, 298 K): δ 1075.50 (t, *Yb*-P, ^1^*J*_YbP_ = 925 Hz). FTIR ν/cm^−1^: 2939 (m), 2879 (m), 1595 (m), 1441 (m), 1229 (m), 1003 (m), 1001 (m), 810 (s), 699 (s), 617 (s).

#### [Sm{P(SiMe_3_)_2_}_2_(18-crown-6)] (**4-Sm**)

To a precooled (−78 °C) suspension of [SmI_2_(THF)_2_] (0.5484 g, 1 mmol) in diethyl ether (10 mL), a suspension of KP″ (0.6494 g, 3 mmol) in diethyl ether (10 mL) was added dropwise. The resultant green reaction mixture was stirred for 1 h at −78 °C before being allowed to warm to room temperature over 20 min. All volatiles were removed *in vacuo*, the green solid was extracted with pentanes (30 mL) and was filtered to yield a deep green solution. All volatiles were removed *in vacuo* and the resulting green solid was extracted into toluene (10 mL). 18-Crown-6 (0.2643 g, 1 mmol) in toluene (10 mL) was added dropwise at room temperature. The green solution was concentrated to *ca*. 5 mL and stored at 3 °C to yield deep green crystals, which were isolated and dried *in vacuo* to afford the title compound. Yield = 0.2668 g, 0.3468 mmol, 35%. Anal. calcd (%) for C_24_H_60_O_6_P_2_Si_4_Sm: C, 37.47; H, 7.86. Found (%): C, 37.45; H, 7.86. ^1^H NMR (400 MHz, C_6_D_6_, 298 K): δ −1.45 (m, 24H, {-C_2_*H*_4_O-}_6_), 2.70 (s, 36H, PSi(C*H*_3_)_3_). ^13^C{^1^H} NMR (101 MHz, C_6_D_6_, 298 K), ^29^Si DEPT90 NMR (79 MHz, C_6_D_6_, 298 K), ^31^P{^1^H} NMR (162 MHz, C_6_D_6_, 298 K): Not observed due to paramagnetic broadening. FTIR ν/cm^−1^: 2931 (m), 2877 (m), 1350 (m), 1229 (m), 1085 (s), 968 (s), 808 (s). 625 (s).

#### [Eu{P(SiMe_3_)_2_}_2_(18-crown-6)] (**4-Eu**)

Prepared by the same method as **4-Sm** using [EuI_2_(THF)_2_] (0.5500 g, 1 mmol) and KP″ (0.6494 g, 3 mmol) to give pale yellow crystals of **4-Eu**. Yield = 0.3548 g, 0.4602 mmol, 46%. Anal. calcd (%) for C_24_H_60_O_6_P_2_Si_4_Eu: C, 37.39; H, 7.84. Found (%): C, 37.52; H, 7.93. ^1^H NMR (400 MHz, C_6_D_6_, 298 K), ^13^C{^1^H} NMR (101 MHz, C_6_D_6_, 298 K), ^29^Si DEPT90 NMR (79 MHz, C_6_D_6_, 298 K), ^31^P{^1^H} NMR (162 MHz, C_6_D_6_, 298 K): Not observed due to paramagnetic broadening. FTIR ν/cm^−1^: 2931 (m), 2875 (m), 1354 (w), 1231 (s), 1100 (s), 968 (s), 806 (s), 621 (s).

#### [Yb{P(SiMe_3_)_2_}_2_(18-crown-6)] (**4-Yb**)

Prepared by the same method as **4-Sm** using [YbI_2_(THF)_2_] (0.5711 g, 1 mmol) and KP″ (0.6494 g, 3 mmol) to give pale yellow crystals of **4-Yb**. Yield = 0.1934 g, 0.2442 mmol, 24%. Anal. calcd (%) for C_24_H_60_O_6_P_2_Si_4_Yb: C, 36.39; H, 7.64. Found (%): C, 35.99; H, 7.77. ^1^H NMR (400 MHz, C_6_D_6_, 298 K): δ 0.56 (s, 36H, PSi(C*H*_3_)_3_), 3.43 (br m, 24H, {-C_2_*H*_4_O-}_6_). ^13^C{^1^H} NMR (101 MHz, C_6_D_6_, 298 K): δ 8.43 (Vir. t, PSi(*C*H_3_)_3_, ^2^*J*_PC_ = 5.6 Hz), 69.06 ({-C_2_*H*_4_O-}_6_). ^29^Si DEPT90 NMR (79 MHz, C_6_D_6_, 298 K): δ 1.94 (Vir. t, *Si*(CH_3_)_3_, ^1^*J*_PSi_ = 17 Hz). ^31^P{^1^H} NMR (162 MHz, C_6_D_6_, 298 K): δ −265.58 (s, *P*-Yb, ^1^*J*_YbP_ = 977 Hz). ^171^Yb{^1^H} NMR (70.67 MHz, C_6_D_6_, 298 K): δ 176.88 (t, *Yb*-P, ^1^*J*_YbP_ = 977 Hz). FTIR ν/cm^−1^: 2935 (m), 2879 (m), 1469 (w), 1354 (w), 1227 (s), 1081 (s), 964 (s), 804 (s), 619 (s).

## Data Availability

Research data files supporting this publication are available from FigShare at https://figshare.com/doi/10.6084/m9.figshare.26213303.

## Supporting Information Available

Additional experimental details, materials, and methods, and data associated with this manuscript (PDF)
