# Supplementary material for: Isolation and Electronic Structures of Lanthanide(II) Bis(trimethylsilyl)phosphide Complexes
Source: Inorg Chem. 2024 Sep 16;63(39):18120–36. doi: 10.1021/acs.inorgchem.4c02888 (PMC11445725; doi:10.1021/acs.inorgchem.4c02888)
Supplement: Supplementary file 1 — ic4c02888_si_001.pdf [file ic4c02888_si_001.pdf]

Supporting Information

Isolation and electronic structures of lanthanide(II) bis(trimethylsilyl)phosphide complexes

Jack Baldwin, Adam Brookfield, George F. S. Whitehead, Louise S. Natrajan,* Eric J. L. McInnes,* Meagan S. Oakley* and David P. Mills*

Department of Chemistry, University of Manchester, Oxford Road, Manchester, M13 9PL, UK.

*E-mail: louise.natrajan@manchester.ac.uk; eric.mcinnes@manchester.ac.uk; meagan.oakley@manchester.ac.uk; david.mills@manchester.ac.uk

Contents

1. **ATR-IR Spectroscopy** S2
2. **Crystallographic Details** S8
3. **Molecular Structures** S11
4. **Powder X-ray Diffraction** S17
5. **NMR Spectroscopy** S18
6. **UV-Vis-NIR Spectroscopy** S28
7. **Photoluminescence Properties: 1-Eu, 2-Yb, 3-Eu, 3-Yb, 4-Eu, 4-Yb** S34
8. **SQUID Magnetometry: 1-Sm, 1-Eu, 3-Sm, 3-Eu, 4-Sm, 4-Eu** S41
9. **EPR Spectroscopy: 1-Eu, 3-Eu, 4-Eu** S47
10. ***Ab initio* Calculations: 1-Sm, 1-Eu, 3-Sm, 3-Eu, 4-Sm, 4-Eu** S75
11. **DFT Calculations: 3-Yb, 4-Yb** S82

1. ATR-IR Spectroscopy

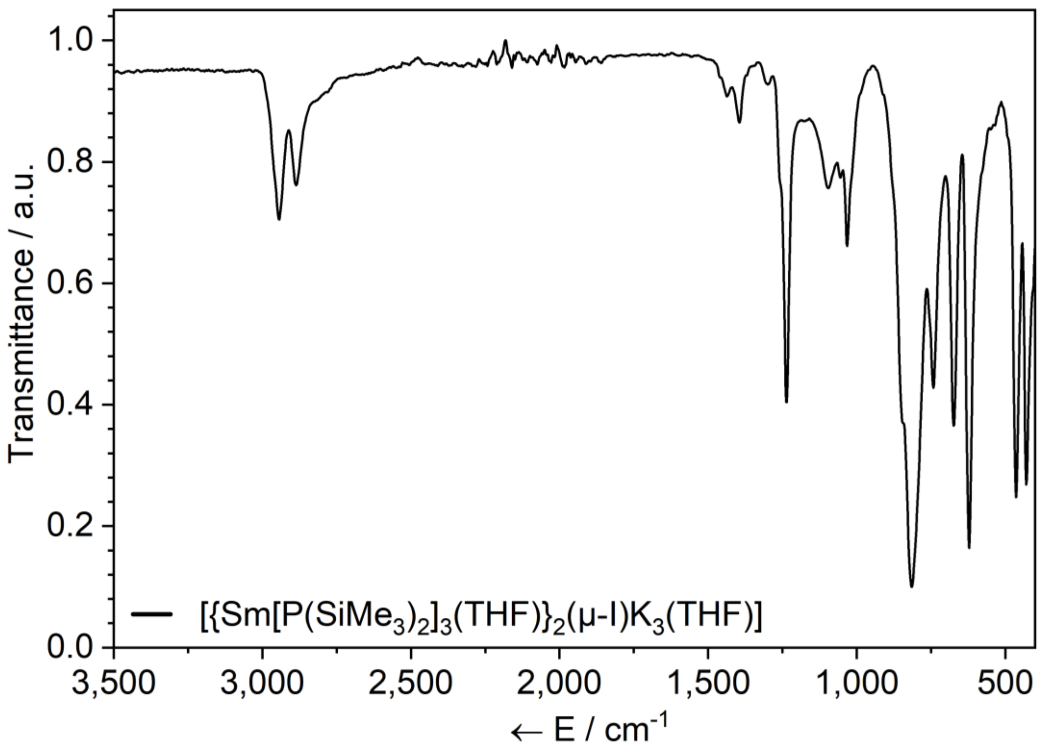

Figure S1. ATR-IR spectrum of **1-Sm** between 398-4000 cm^{-1}.

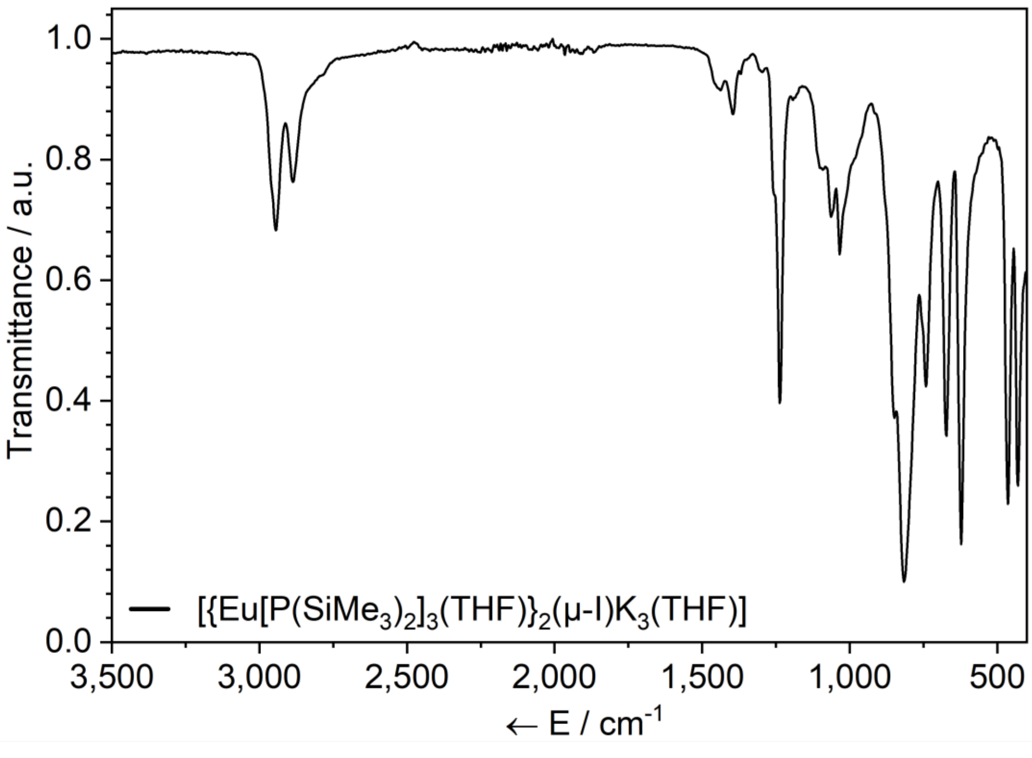

Figure S2. ATR-IR spectrum of **1-Eu** between 398-4000 cm^{-1}.

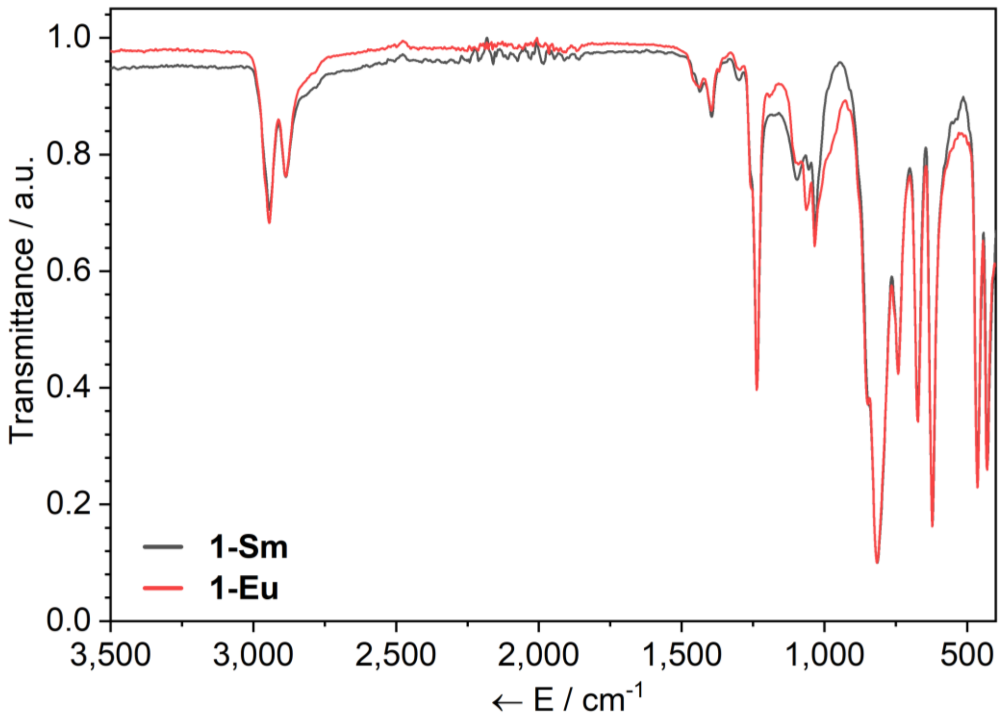

Figure S3. ATR-IR spectra of **1-Sm** and **1-Eu** between 398-4000 cm^{-1}.

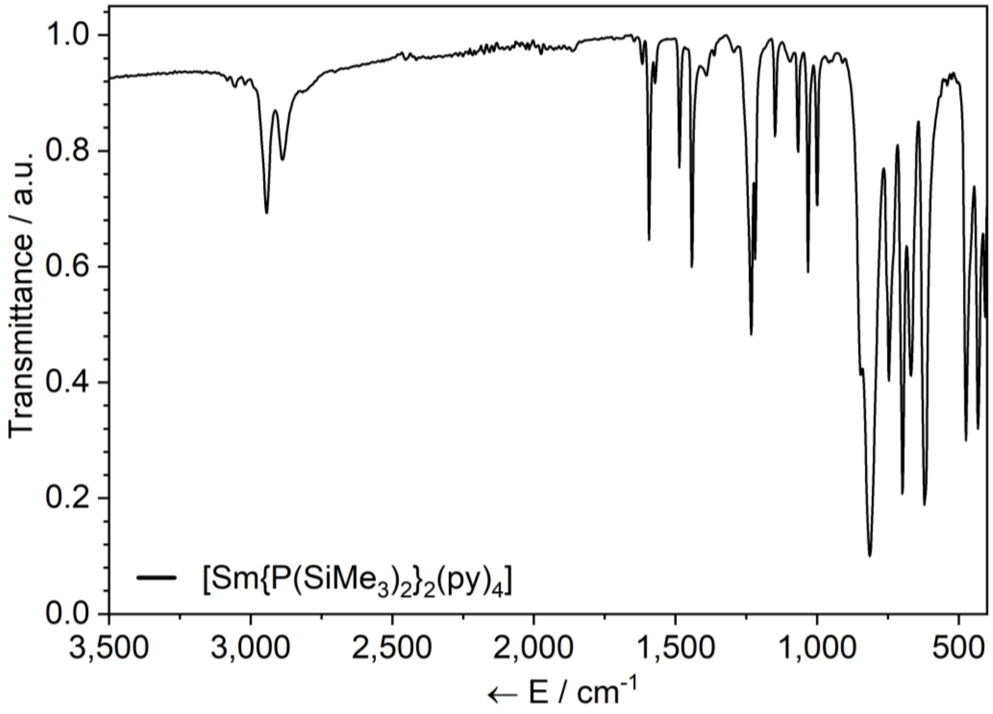

Figure S4. ATR-IR spectrum of **3-Sm** between 398-4000 cm^{-1}.

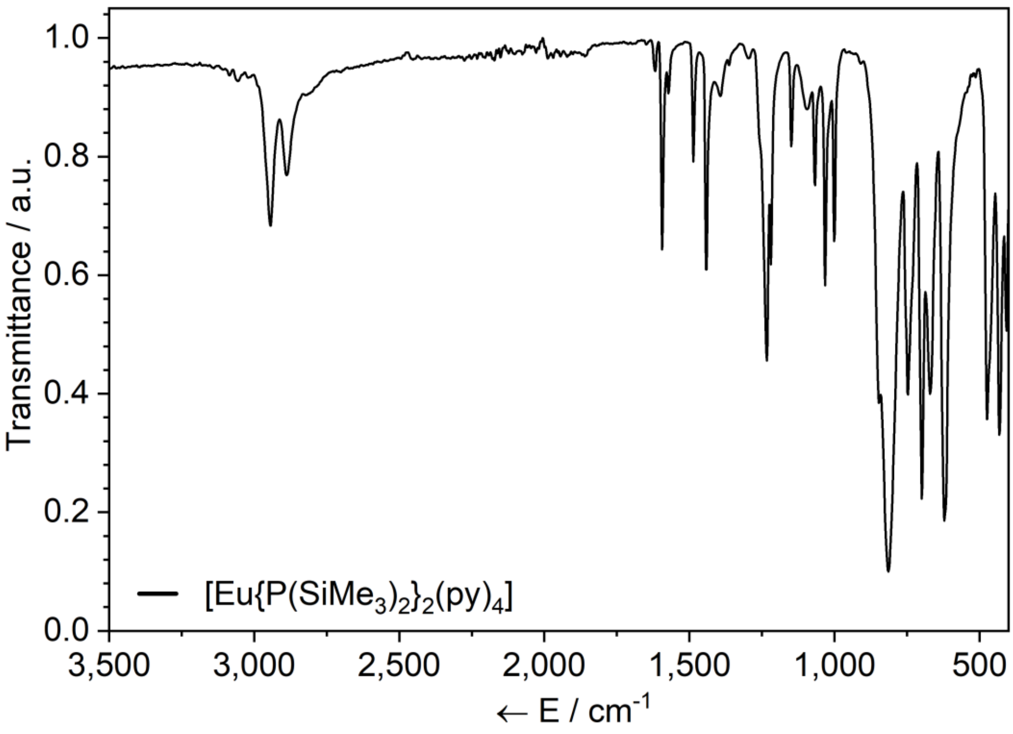

Figure S5. ATR-IR spectrum of **3-Eu** between 398-4000 cm^{-1}.

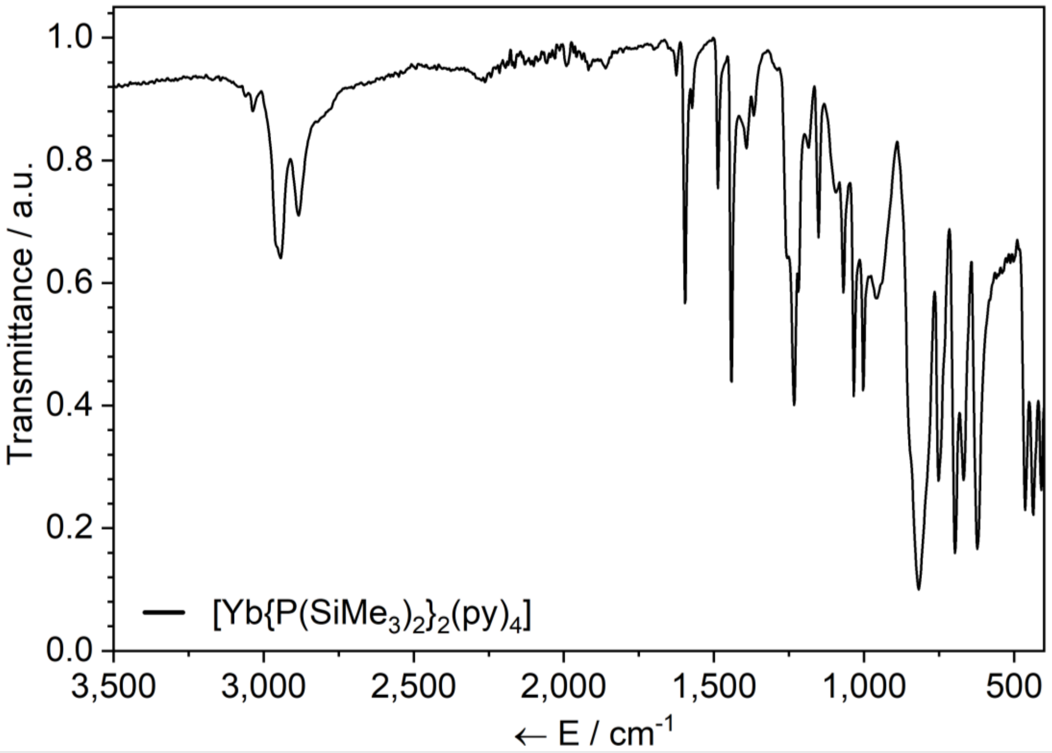

Figure S6. ATR-IR spectrum of **3-Yb** between 398-4000 cm^{-1}.

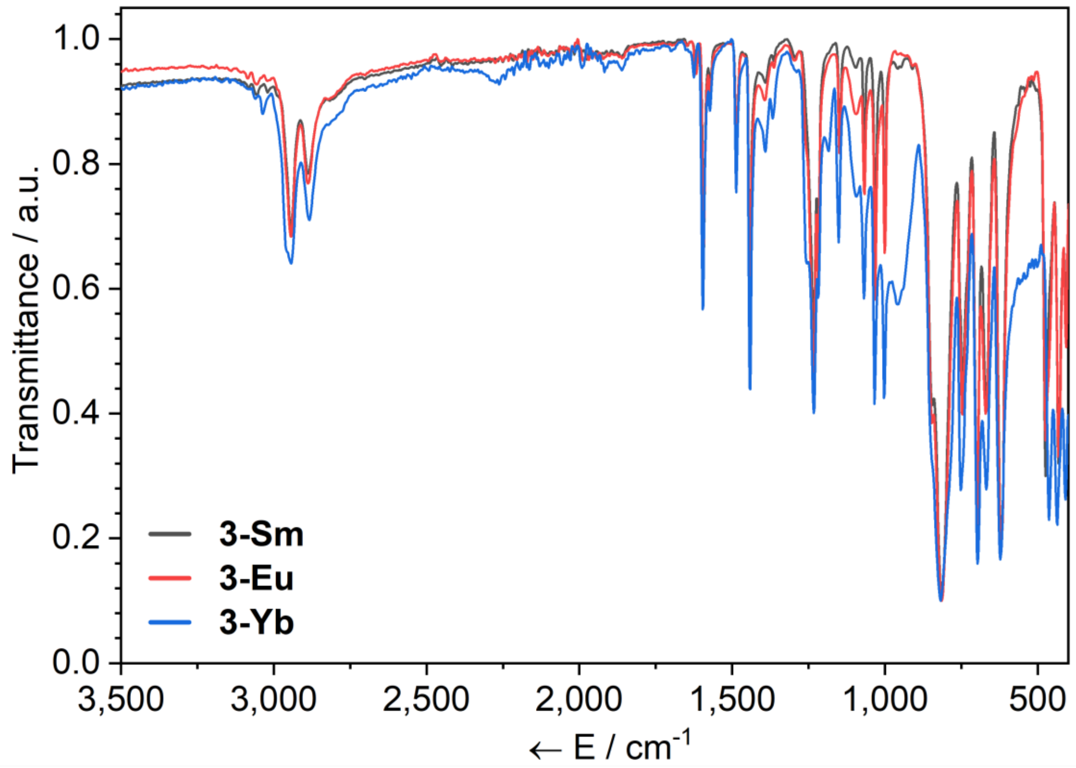

Figure S7. ATR-IR spectra of **3-Sm**, **3-Eu** and **3-Yb** between 398-4000 cm-1.

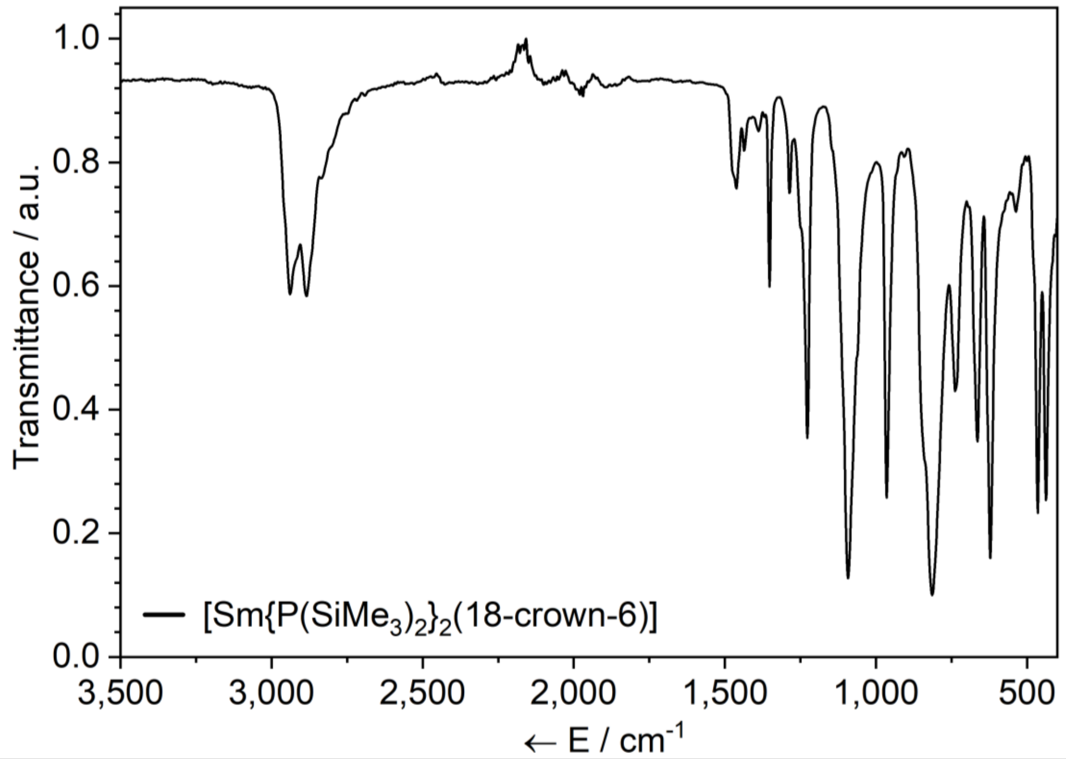

Figure S8. ATR-IR spectrum of **4-Sm** between 398-4000 cm-1.

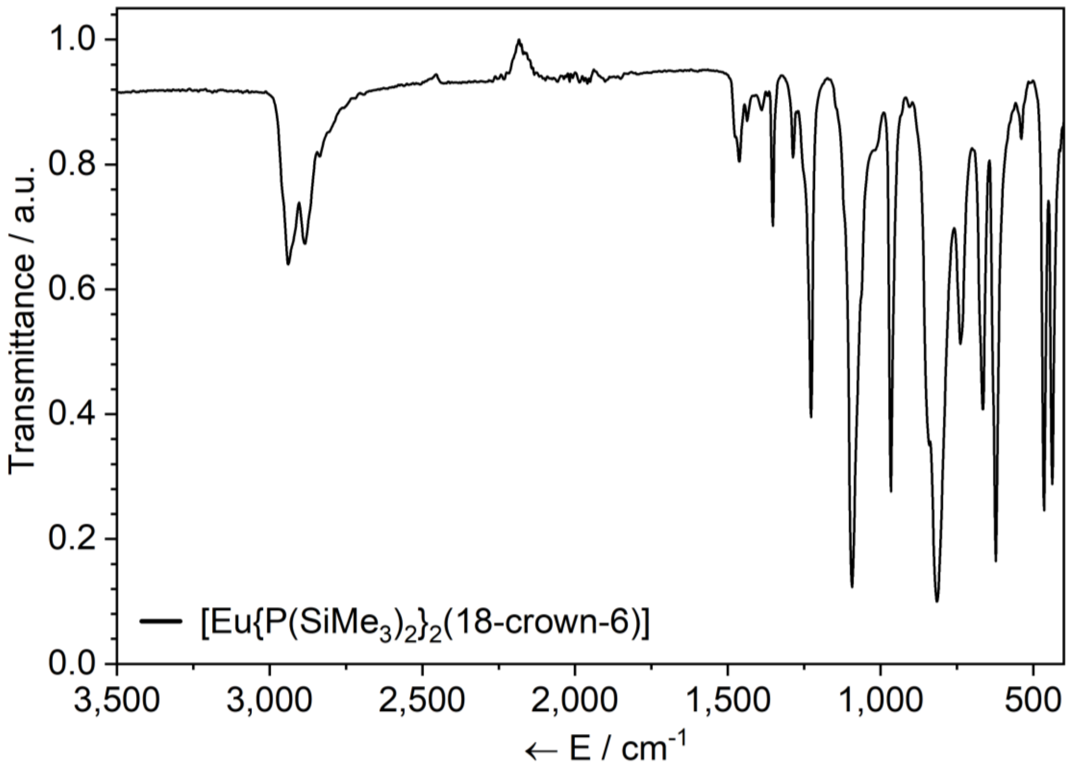

Figure S9. ATR-IR spectrum of **4-Eu** between 398-4000 cm^{-1}.

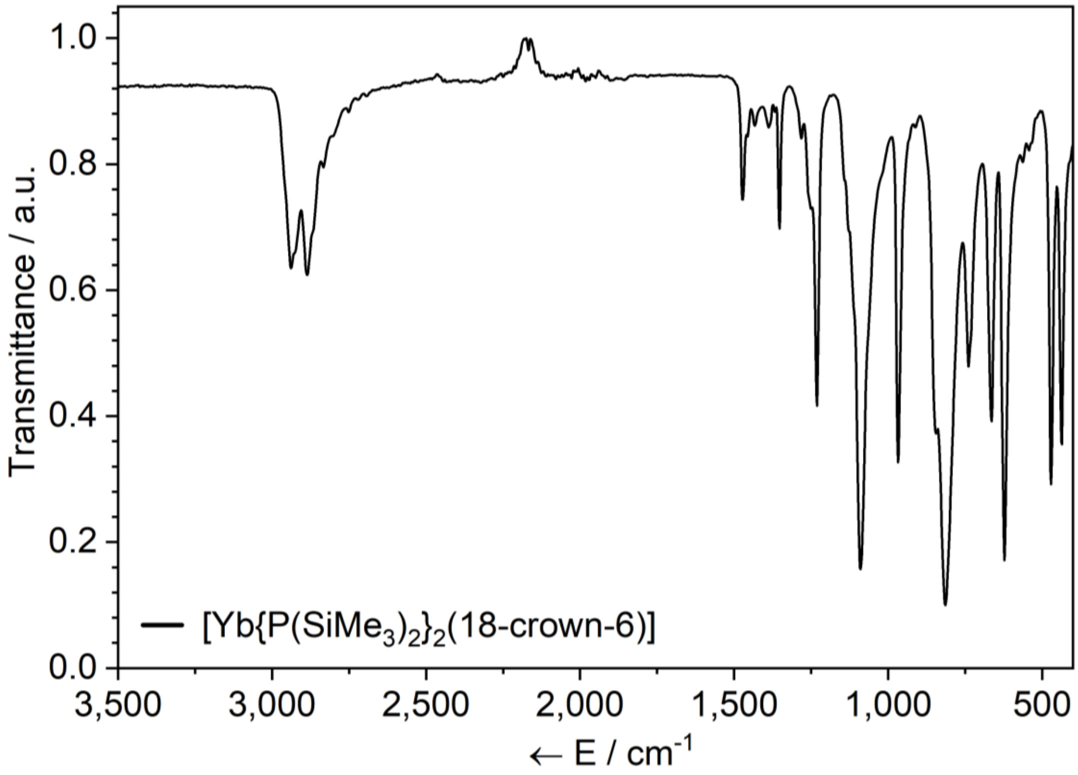

Figure S10. ATR-IR spectrum of **4-Yb** between 398-4000 cm^{-1}.

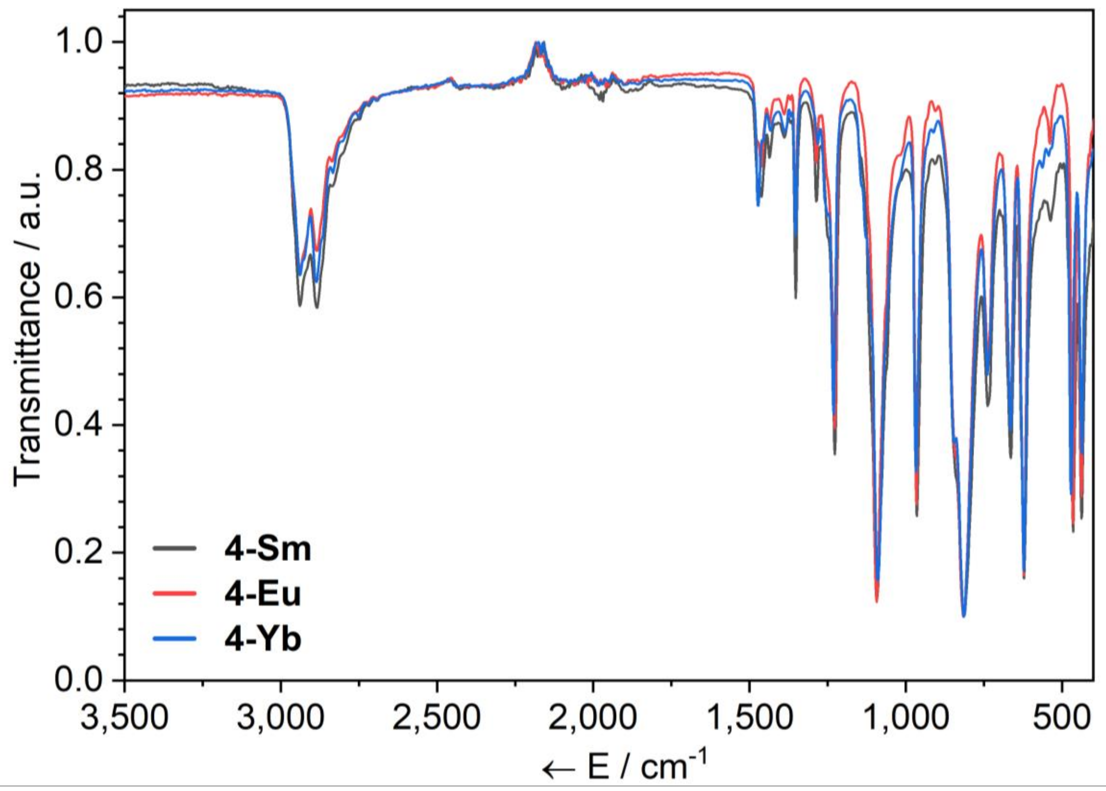

Figure S11. ATR-IR spectra of **4-Sm**, **4-Eu** and **4-Yb** between 398-4000 cm^{-1}.

2. Crystallographic Details

Table S1. Crystallographic data for [{Ln[P(SiMe$_3$)$_2$]$_3$(THF)}$_2$(μ-I)K$_3$(THF)] (**1-Ln**; Ln = Sm, Eu) and [KYb{P(SiMe$_3$)$_2$}$_3${μ-K[P(SiMe$_3$)$_2$]}$_2$]$_\infty$ (**2-Yb**). [a]Conventional $R = \Sigma||F_o| - |F_c||/\Sigma|F_o|$; $R_w = [\Sigma w(F_o^2 - F_c^2)^2/\Sigma w(F_o^2)^2]^{1/2}$; $S = [\Sigma w(F_o^2 - F_c^2)^2$ /no. data – no. params)]$^{1/2}$ for all data.

	**1-Sm**	**1-Eu**	**2-Yb**
Formula	$C_{48}H_{132}IK_3O_3P_6Si_{12}Sm_2$	$C_{48}H_{132}IK_3O_3P_6Si_{12}Eu_2$	$C_{30}H_{90}K_3P_5Si_{10}Yb$
Fw	1825.33	1828.55	1177.10
cryst size, mm	0.192×0.053×0.038	0.366×0.096×0.062	0.111×0.036×0.026
cryst syst	monoclinic	monoclinic	monoclinic
space group	$P2_1/c$	$P2_1/c$	Cc
a, Å	16.5763(6)	16.6586(5)	21.5862(16)
b, Å	25.1810(10)	24.9506(5)	13.1550(5)
c, Å	22.6282(8)	22.6595(5)	25.429(2)
α, °	90	90	90
β, °	103.822(4)	103.820(3)	121.424(11)
γ, °	90	90	90
V, Å^3	9171.7(6)	9145.6(4)	6162.0(9)
Z	4	4	4
ρ_{calc}, g/cm^3	1.322	1.328	1.269
μ, mm^{-1}	16.099	2.125	7.858
F(000)	3744	3752	2448
no. of unique reflns, *R*int	18595	22646	11147
R, *Rwa* ($F^2 > 2\sigma(F^2)$)	0.0547, 0.1365	0.0469, 0.0823	0.0750, 0.2017
S[a]	1.096	1.006	1.129
Rint	0.0660	0.0677	0.0801
max., min. diff map, e Å^{-3}	2.778, –3.157	2.066, –1.033	0.733, –1.198

Table S2. Crystallographic data for *trans*-[Ln{P(SiMe$_3$)$_2$}$_2$(py)$_4$] (**3-Ln**; Ln = Sm, Eu, Yb). ^{a}Conventional $R = \Sigma||F_o| - |F_c||/\Sigma|F_o|$; $R_w = [\Sigma w(F_o^2 - F_c^2)^2/\Sigma w(F_o^2)^2]^{1/2}$; S = $[\Sigma w(F_o^2 - F_c^2)^2$ /no. data – no. params)$]^{1/2}$ for all data.

	**3-Sm**	**3-Eu**	**3-Yb**
Formula	$C_{32}H_{56}N_4P_2Si_4Sm$	$C_{32}H_{56}N_4P_2Si_4Eu$	$C_{32}H_{56}N_4P_2Si_4Yb$
Fw	821.45	823.06	844.15
cryst size, mm	0.13×0.038×0.033	0.186×0.045×0.023	0.178×0.113×0.091
cryst syst	monoclinic	monoclinic	monoclinic
space group	$P2_1/n$	C_2/c	$P2_1/c$
a, Å	20.3285(5)	23.6113(4)	12.79670(10)
b, Å	9.4057(2)	9.3414(2)	27.7096(3)
c, Å	22.7303(4)	19.6964(4)	24.4703(3)
α, °	90	90	90
β, °	96.581(2)	101.428(2)	103.4880(10)
γ, °	90	90	90
V, Å^3	4317.48(16)	4258.16(15)	8437.64(16)
Z	4	4	8
ρ_{calc}, g/cm^3	1.264	1.284	1.329
μ, mm^{-1}	12.159	12.517	6.091
F(000)	1696	1700	3456
no. of unique reflns, *R*int	8773	4371	33382
R, *Rwa* ($F^2 > 2\sigma(F^2)$)	0.0543, 0.1414	0.0366, 0.0919	0.0394, 0.1215
S^a	1.038	1.051	1.113
Rint	0.0695	0.0572	0.0177
max., min. diff map, e Å^{-3}	1.329, –1.520	1.965, –0.844	1.033, –1.785

Table S3. Crystallographic data for [Ln{P($SiMe_3$)$_2$}$_2$(18-crown-6)] (**4-Ln**; Ln = Sm, Eu, Yb). [a]Conventional $R = \Sigma||F_o| - |F_c||/\Sigma|F_o|$; $R_w = [\Sigma w(F_o^2 - F_c^2)^2/\Sigma w(F_o^2)^2]^{1/2}$; $S = [\Sigma w(F_o^2 - F_c^2)^2$ /no. data – no. params)]$^{1/2}$ for all data.

	4-Sm	4-Eu	4-Yb
Formula	$C_{24}H_{60}O_6P_2Si_4Sm$	$C_{24}H_{60}O_6P_2Si_4Eu$	$C_{24}H_{60}O_6P_2Si_4Yb$
Fw	769.37	770.98	792.06
cryst size, mm	0.169×0.084×0.017	0.165×0.102×0.050	0.187×0.15×0.082
cryst syst	triclinic	monoclinic	monoclinic
space group	P-1	$P2_1/c$	I2/c
a, Å	15.4109(6)	16.3464(9)	14.4269(7)
b, Å	16.1932(9)	30.7558(12)	13.4956(6)
c, Å	16.6636(14)	16.6108(9)	20.6890(10)
α, °	115.320(7)	90	90
β, °	91.696(5)	115.581(7)	108.395(10)
γ, °	90.145(4)	90	90
V, Å^3	3756.8(5)	7532.4(8)	3822.3(3)
Z	4	8	4
ρ_{calc}, g/cm^3	1.360	1.360	1.376
μ, mm^{-1}	1.807	14.196	6.767
F(000)	1600	3208	1632
no. of unique reflns, *R*int	20623	14419	11595
R, *Rwa* ($F^2 > 2\sigma(F^2)$)	0.0501, 0.1294	0.0772, 0.1957	0.0393, 0.1198
S[a]	1.073	1.037	1.087
Rint	0.0449	0.0880	0.0253
max., min. diff map, e Å^{-3}	2.377, –1.116	3.909, –1.876	1.185, –1.548

Table S4. τ_4 and τ_5 shape analysis for **1-M** and **2-Yb** (M = Sm, Eu).

**Structure***	τ_4 [a]	τ_5 [b]
**1-Sm**	-	Sm(1) = 0.8460<br>Sm(2) = 0.8740
**1-Eu**	-	Eu(1) = 0.8595<br>Eu(2) = 0.8205
**2-Yb**	0.7633	-

$$^{a}\ \tau_4 = \frac{360° - (\alpha + \beta)}{360° - \theta} \approx -0.00709\alpha - 0.00709\beta + 2.55 \qquad (1)$$

$$^{b}\ \tau_5 = \frac{\beta - \alpha}{60°} \approx -0.01667\alpha + 0.01667\beta \qquad (2)$$

where: β > α are the two greatest valence angles of the coordination center.

* When $\tau_4 = 0$, square planar, when $\tau_4 = 1$, tetrahedral.

When $\tau_5 = 0$, square pyramidal, when $\tau_5 = 1$, trigonal bipyramidal.

Table S5. Continuous Shape Measures (CShM) calculations for **3-Ln** (Ln = Sm, Eu, Yb).

**Structure**[a]	OC-6	TPR-6
**3-Sm CShM**	4.200	9.048
**3-Eu CShM**	4.029	9.066
**3-Yb CShM**	0.530	16.701

[a] OC-6 Octahedron (Oh)

TPR-6 Trigonal prism (D_{3h})

3. Molecular Structures

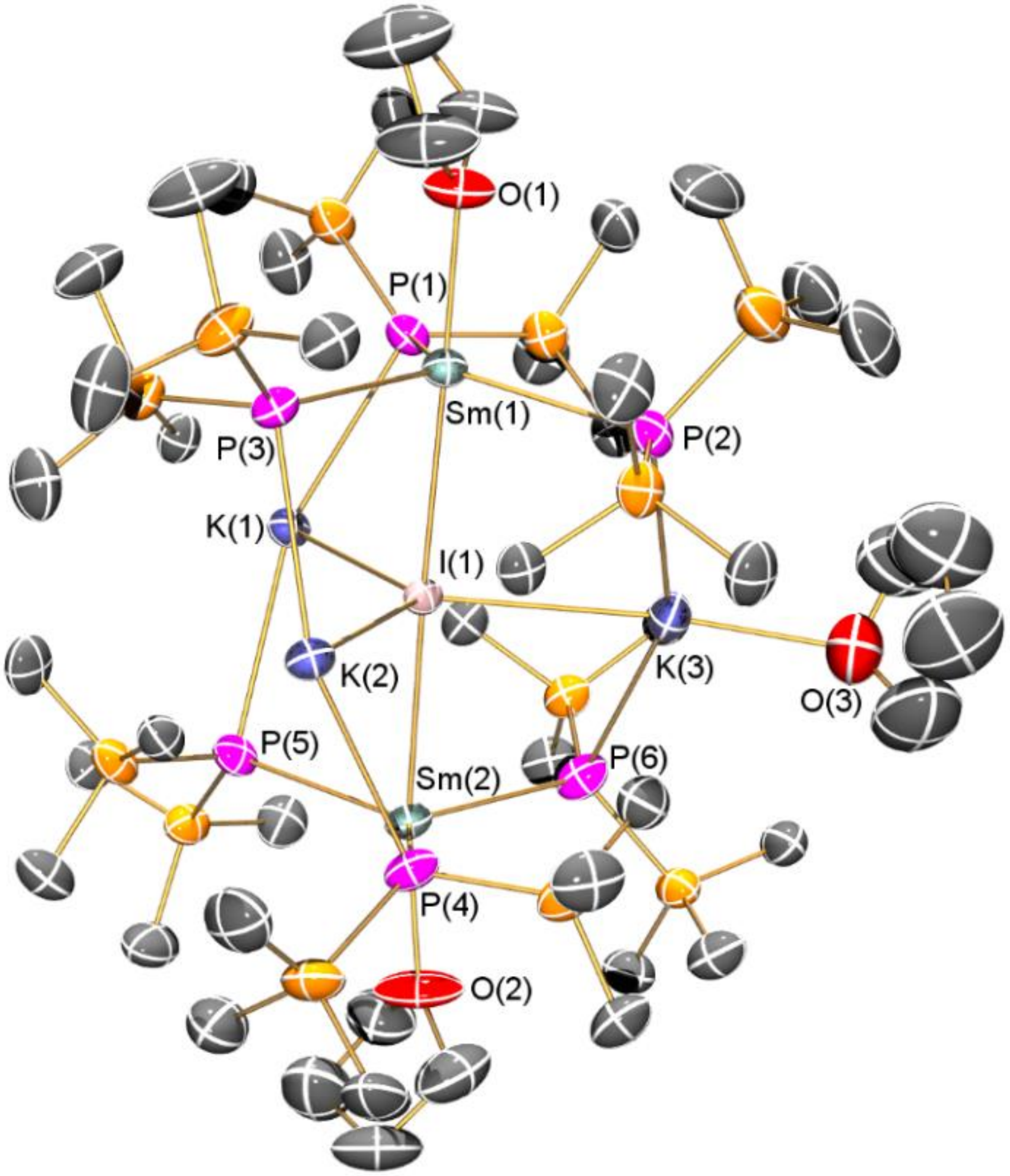

Figure S12. Solid state structure of **1-Sm** with selected atomic labeling; Ln = cyan, P = magenta, Si = yellow, I = pink, K = navy blue, O = red, N = blue, C = gray. Displacement ellipsoids set at 50% probability level, hydrogen atoms omitted for clarity.

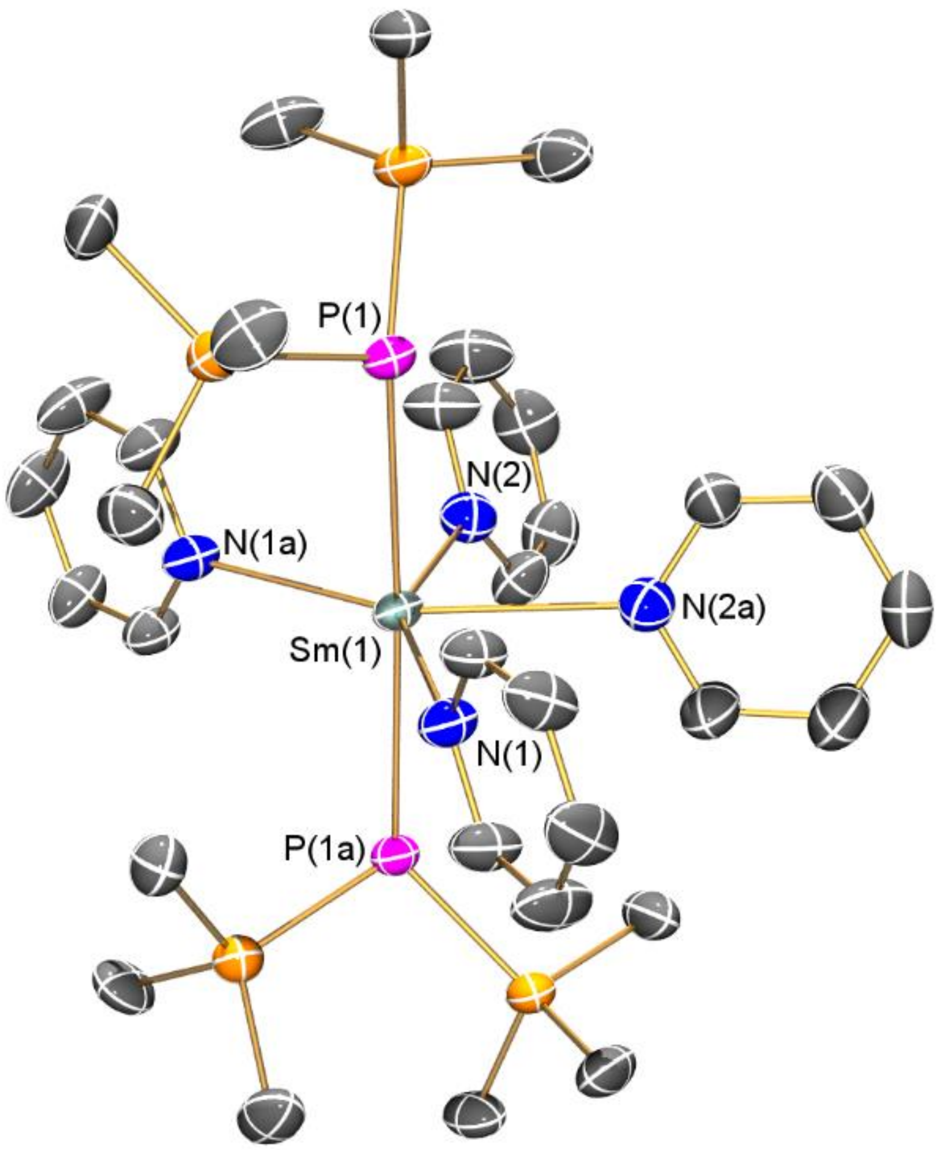

Figure S13. Solid state structure of **3-Sm** with selected atomic labeling; Ln = cyan, P = magenta, Si = yellow, N = blue, C = gray. Displacement ellipsoids set at 50% probability level, hydrogen atoms omitted for clarity.

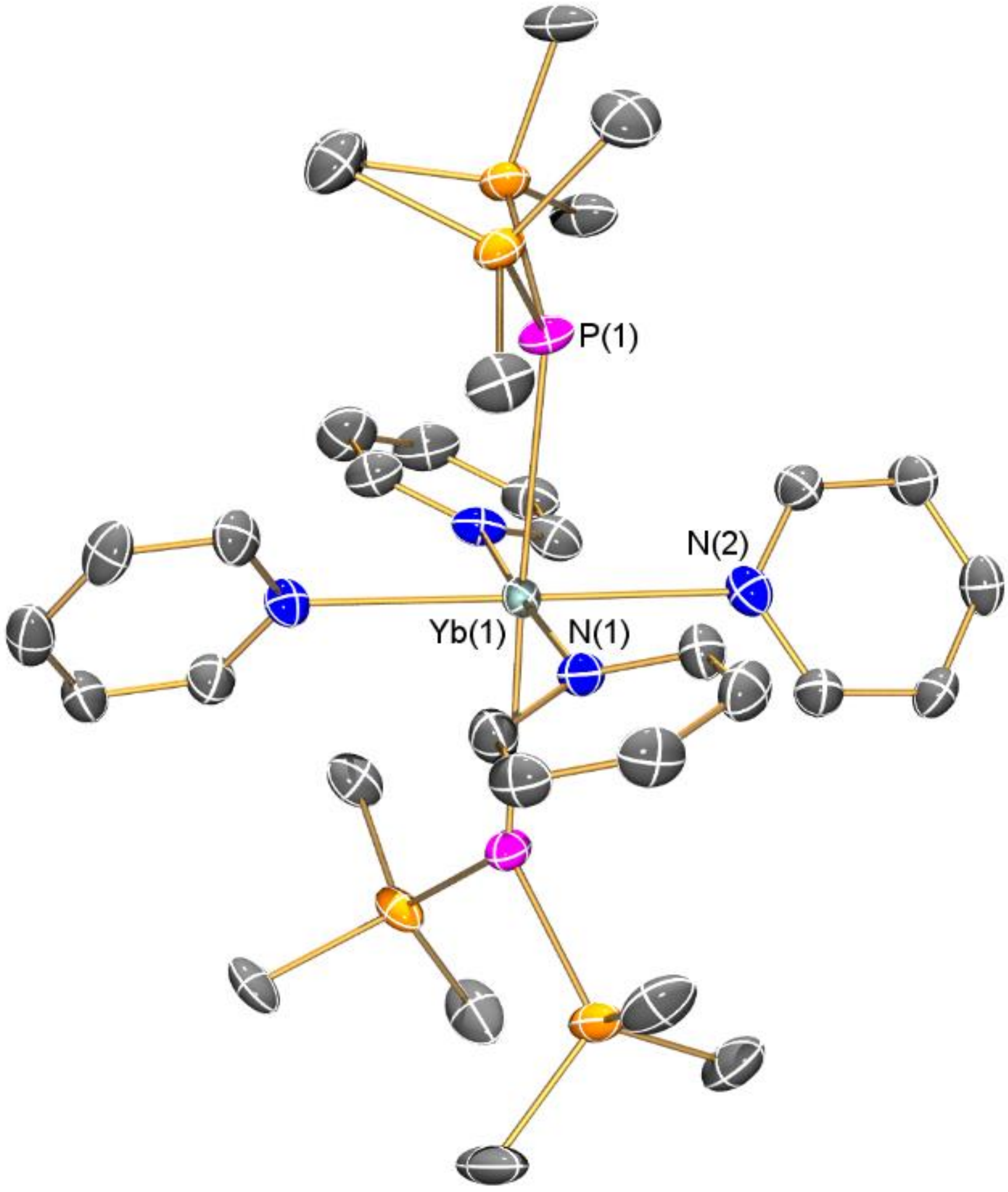

Figure S14. Solid state structure of **3-Yb** with selected atomic labeling; Ln = cyan, P = magenta, Si = yellow, , N = blue, C = gray. Displacement ellipsoids set at 50% probability level, hydrogen atoms omitted for clarity.

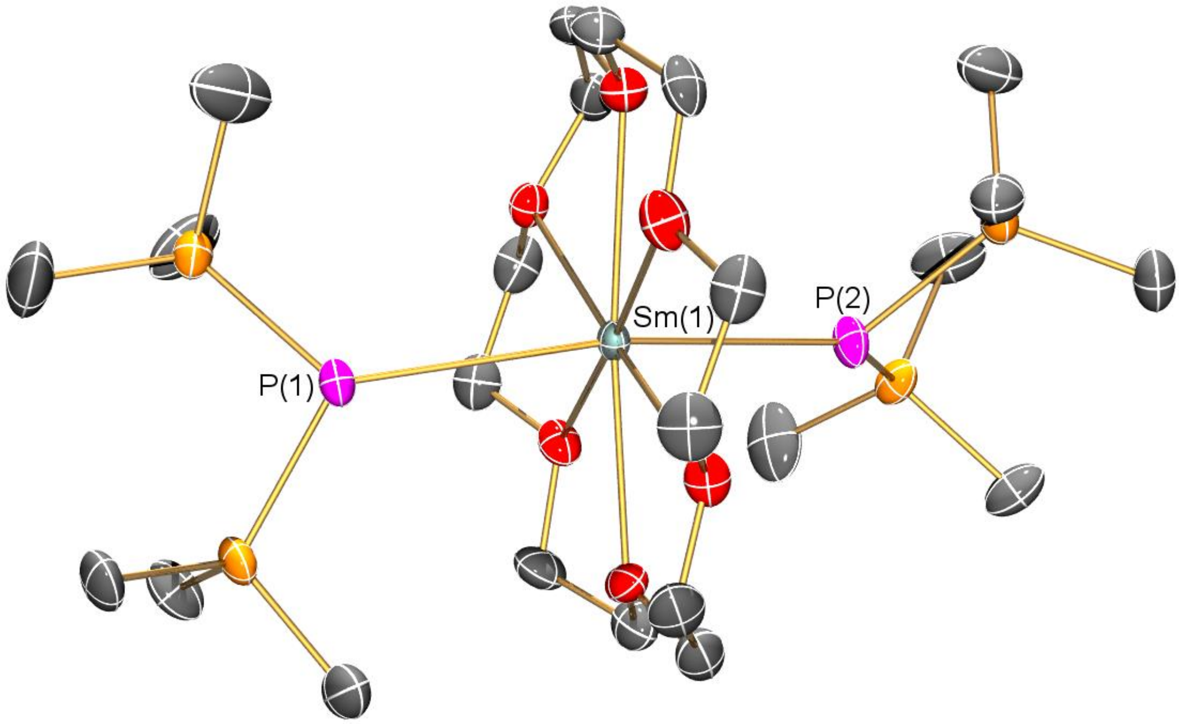

Figure S15. Solid state structure of **4-Sm** with selected atomic labeling; Ln = cyan, P = magenta, Si = yellow, O = red, C = gray. Displacement ellipsoids set at 50% probability level, hydrogen atoms omitted for clarity.

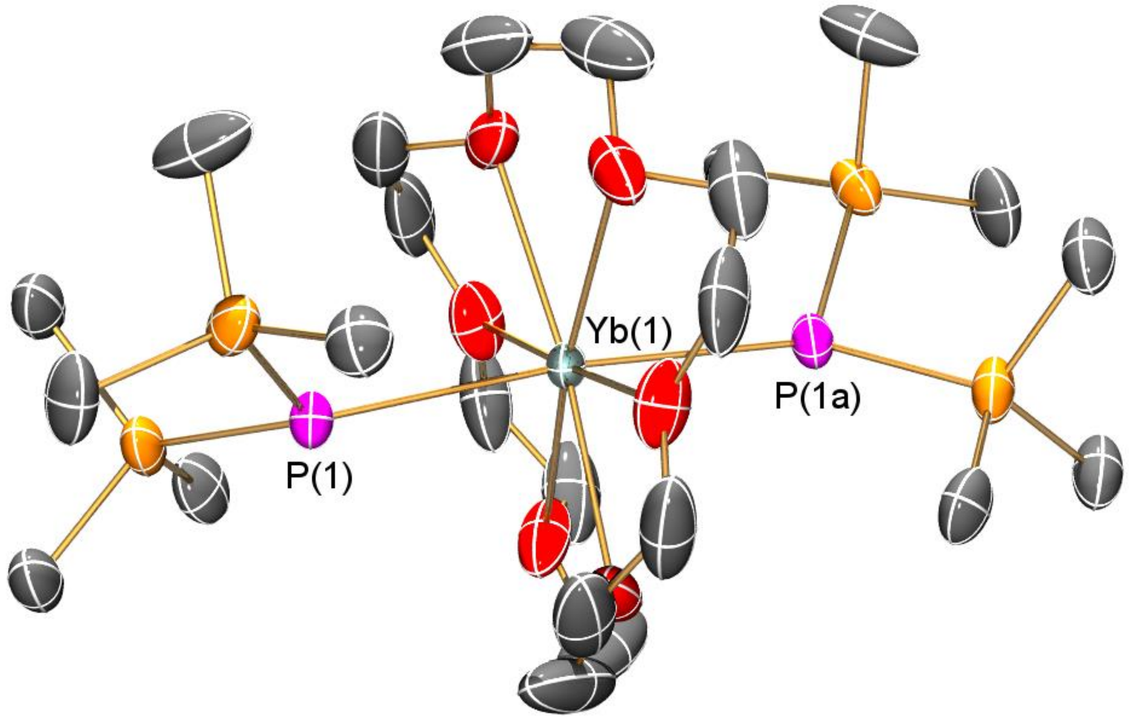

Figure S16. Solid state structure of **4-Yb** with selected atomic labeling; Ln = cyan, P = magenta, Si = yellow, I = pink, K = navy blue, O = red, N = blue, C = gray. Displacement ellipsoids set at 50% probability level, hydrogen atoms omitted for clarity.

4. Powder X-ray Diffraction

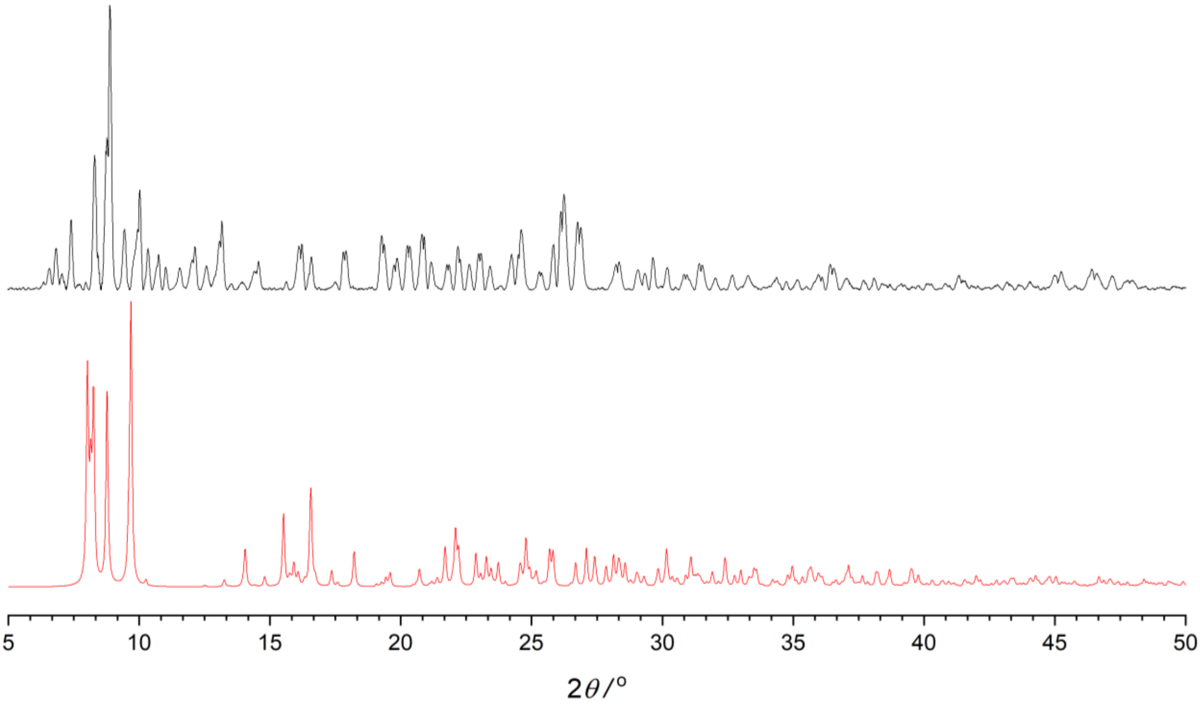

Figure S17. Simulated X-ray diffraction pattern from single crystal X-ray diffraction at 150 K (red) compared to experimental powder XRD pattern at 100 K (black) for **2-Yb** (arbitrary intensities).

5. NMR Spectroscopy

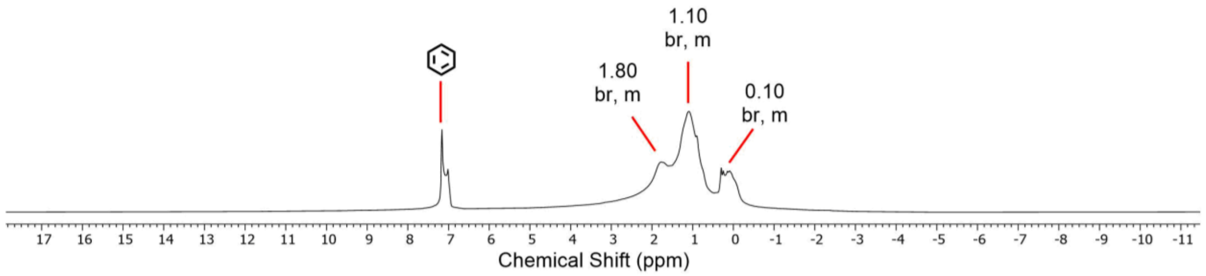

Figure S18 ^{1}H NMR spectrum (400 MHz) of **1-Sm** in C_6D_6. Expansion of spectrum of spectral width -100–100 ppm.

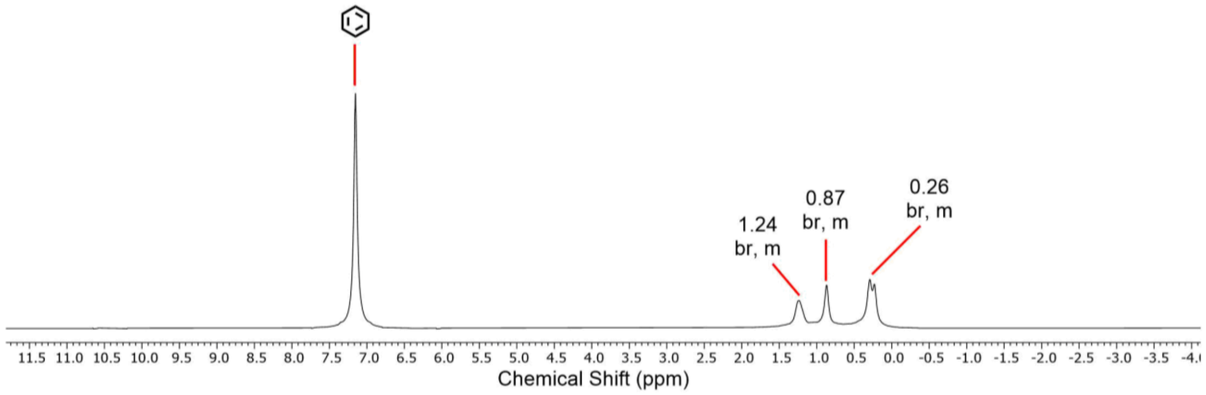

Figure S19. ^{1}H NMR spectrum (400 MHz) of **1-Eu** in C_6D_6. Expansion of spectrum of spectral width -100–100 ppm.

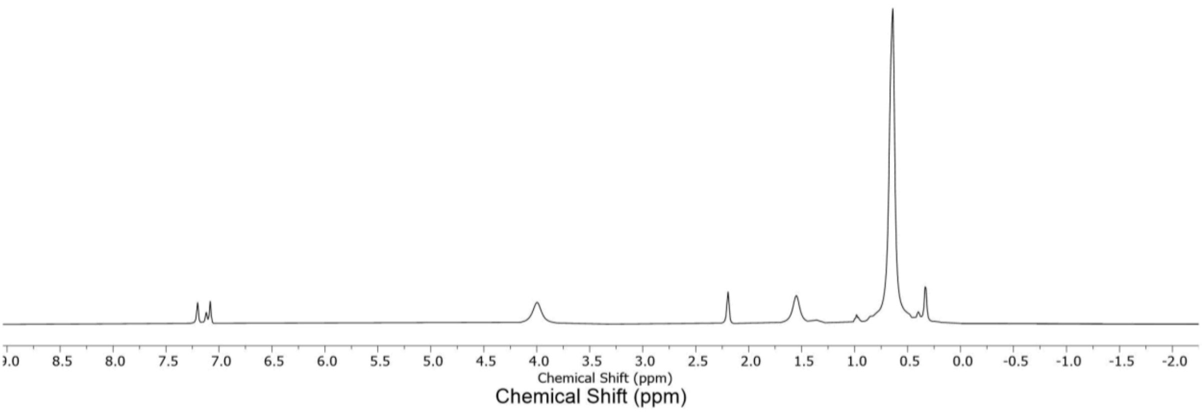

Figure S20. ^{1}H NMR spectrum (400 MHz) of mixture containing **2-Yb** in d_8-toluene.

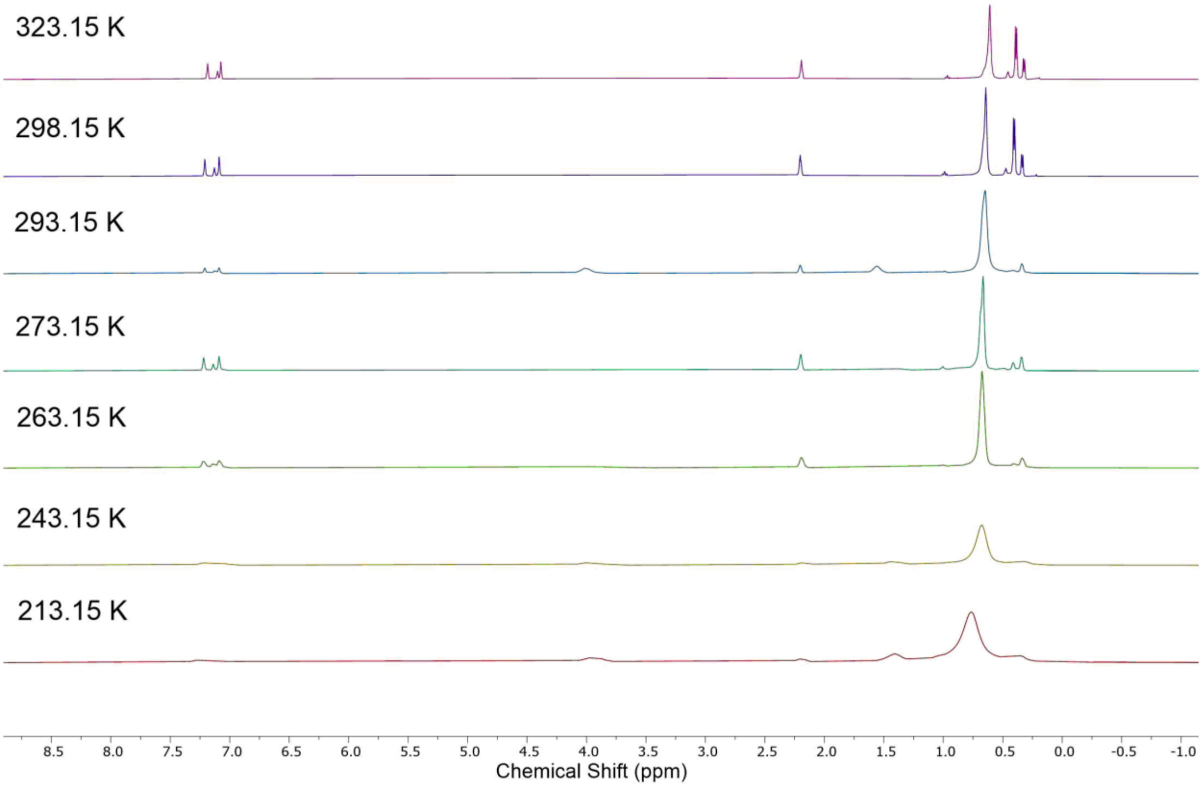

Figure S21. VT ^{1}H NMR spectra (400 MHz) of mixture containing **2-Yb** in d_8-toluene (213.15 to 323.15 K).

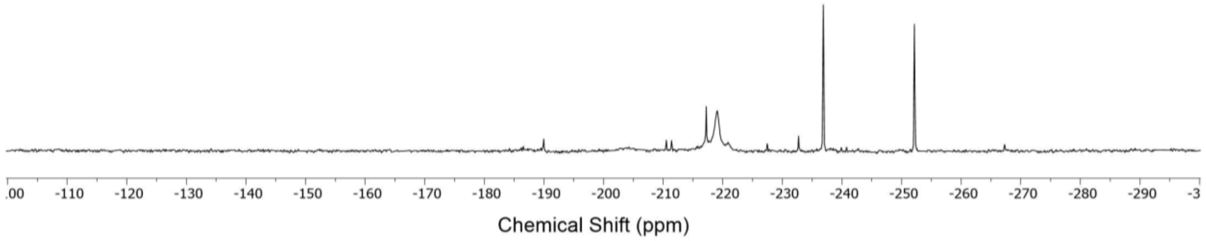

Figure S22. $^{31}P\{^1H\}$ NMR spectrum (162 MHz) of mixture containing **2-Yb** in d_8-toluene.

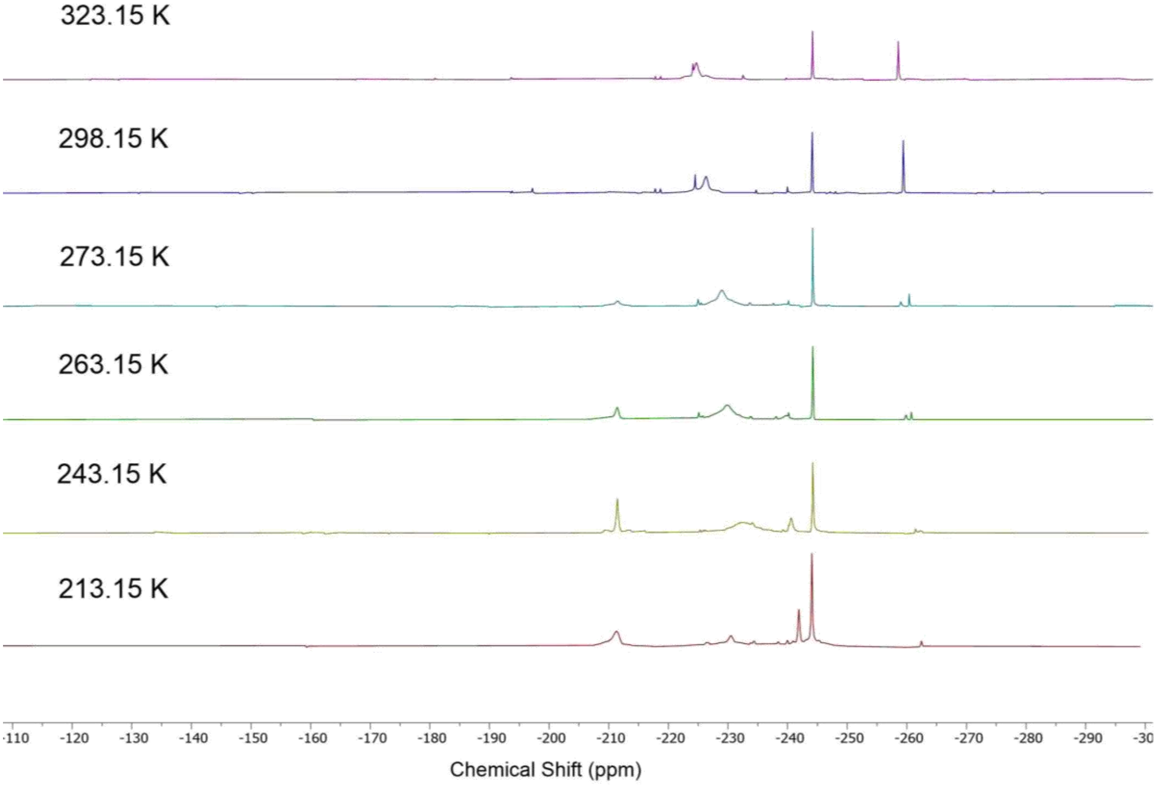

Figure S23. VT $^{31}P\{^1H\}$ NMR spectra (162 MHz) of mixture containing **2-Yb** in d_8-toluene (213.15 to 323.15 K).

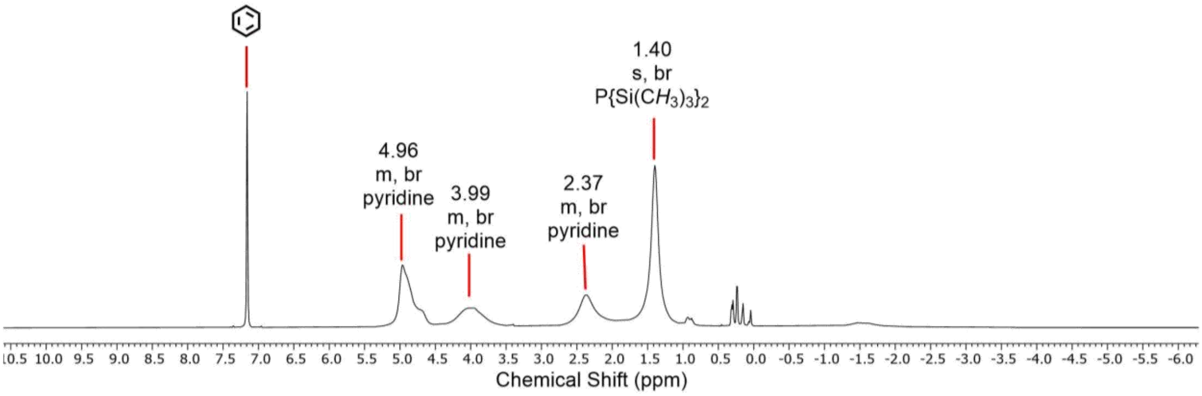

Figure S24. ^{1}H NMR spectrum (400 MHz) of **3-Sm** in C_6D_6 with drops of pyridine. Expansion of spectrum of spectral width -100–100 ppm.

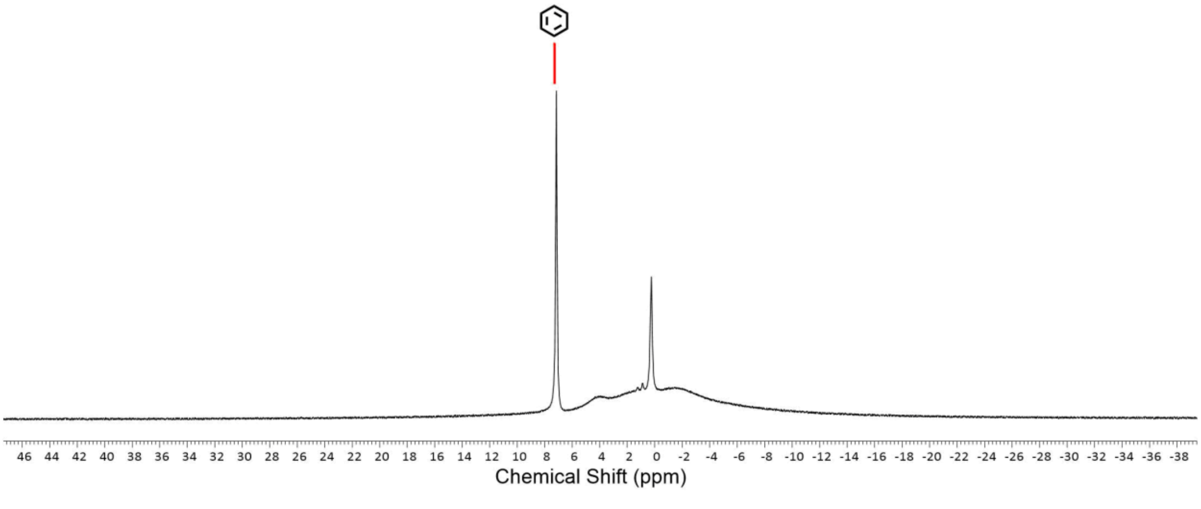

Figure S25. ^{1}H NMR spectrum (400 MHz) of **3-Eu** in C_6D_6 with drops of pyridine. Expansion of spectrum of spectral width -100–100 ppm.

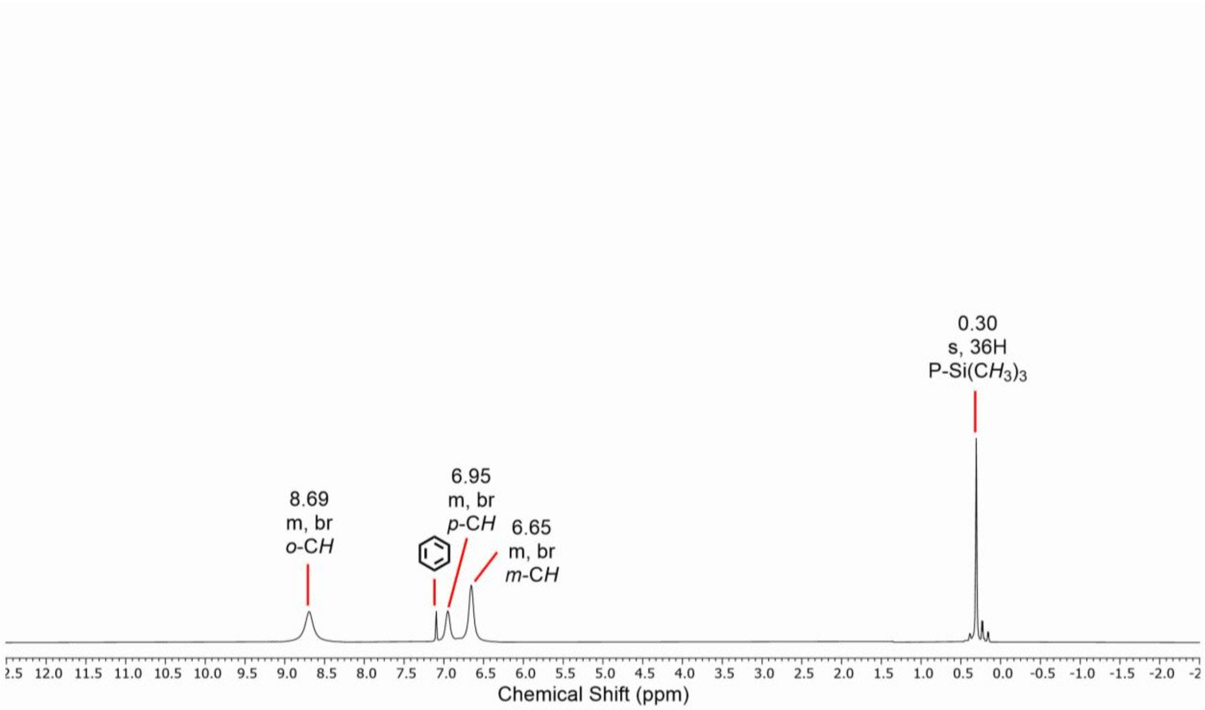

Figure S26. ^{1}H NMR spectrum (400 MHz) of **3-Yb** in C_6D_6 with drops of pyridine.

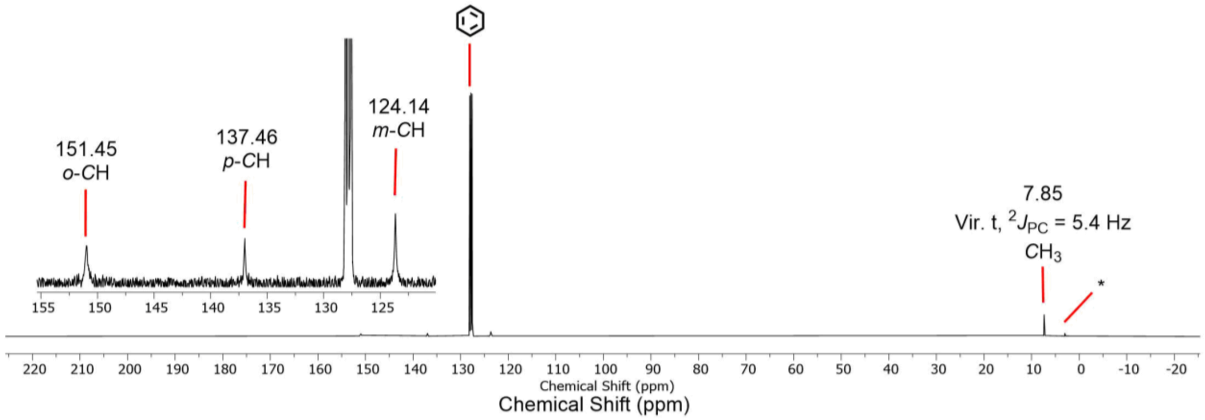

Figure S27. $^{13}C\{^{1}H\}$ NMR spectrum (101 MHz) of **3-Yb** in C_6D_6 with drops of pyridine. * denotes silicon grease.

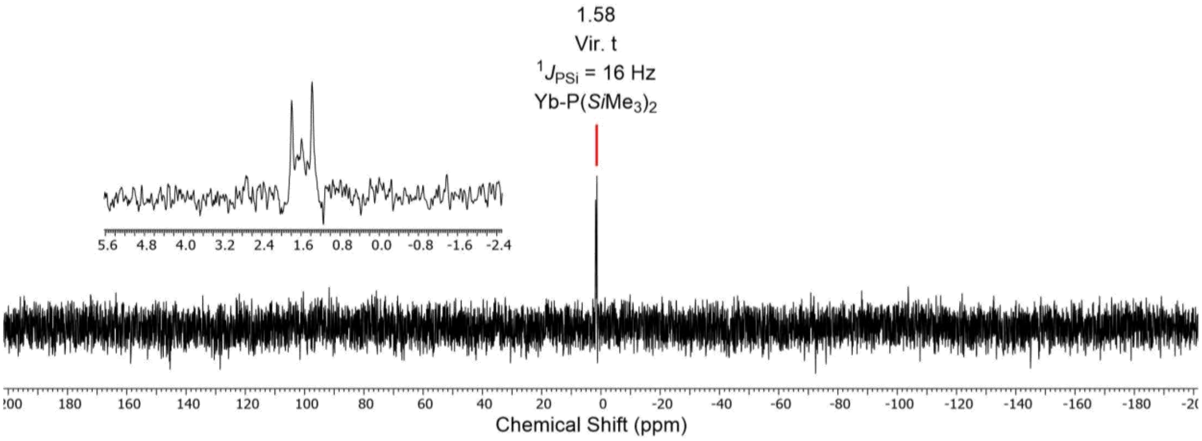

Figure S28. ^{29}Si DEPT90 NMR spectrum (79 MHz) of **3-Yb** in C_6D_6 with drops of pyridine.

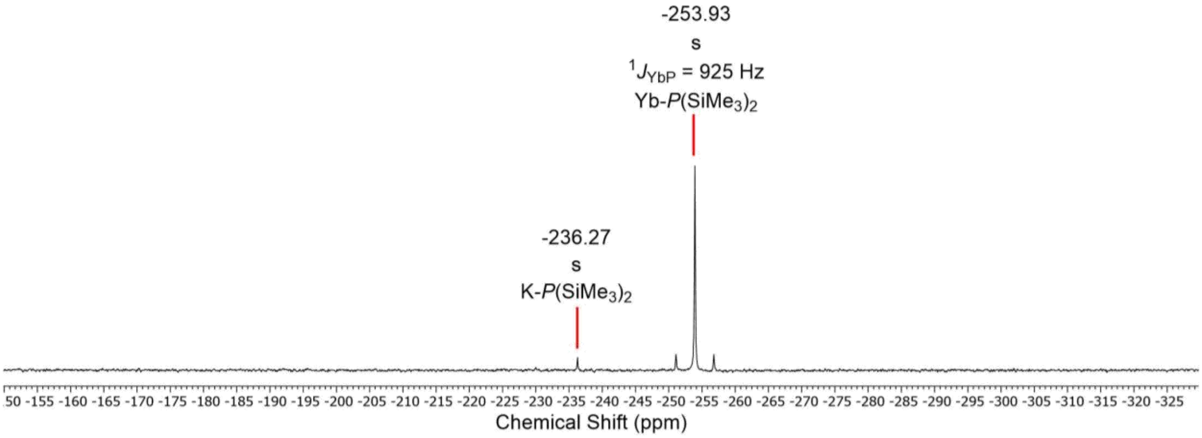

Figure S29. $^{31}P\{^{1}H\}$ NMR spectrum (162 MHz) of **3-Yb** in C_6D_6 with drops of pyridine.

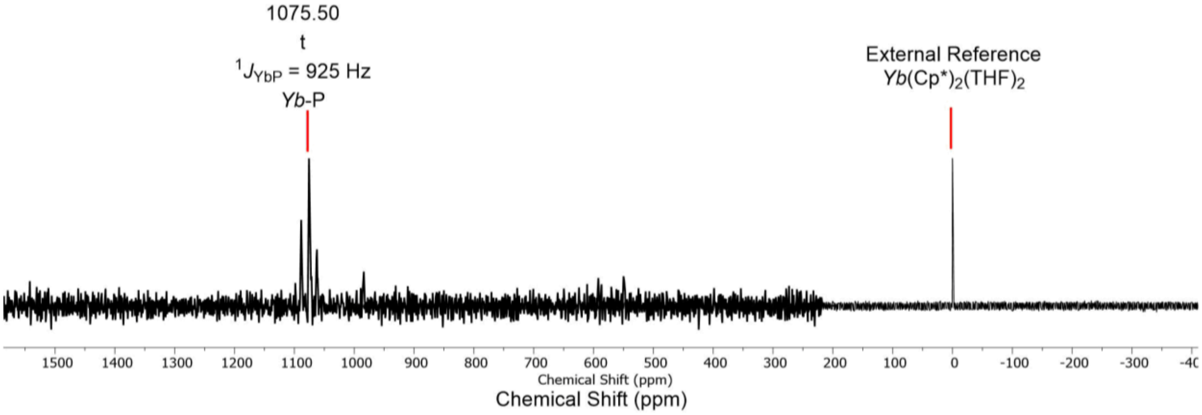

Figure S30. ^{171}Yb{^{1}H} NMR spectrum (71 MHz) of **3-Yb** in C_6D_6 with drops of pyridine. [Yb(Cp*)$_2$(THF)$_2$] reference shown in order to show relative chemical shift.

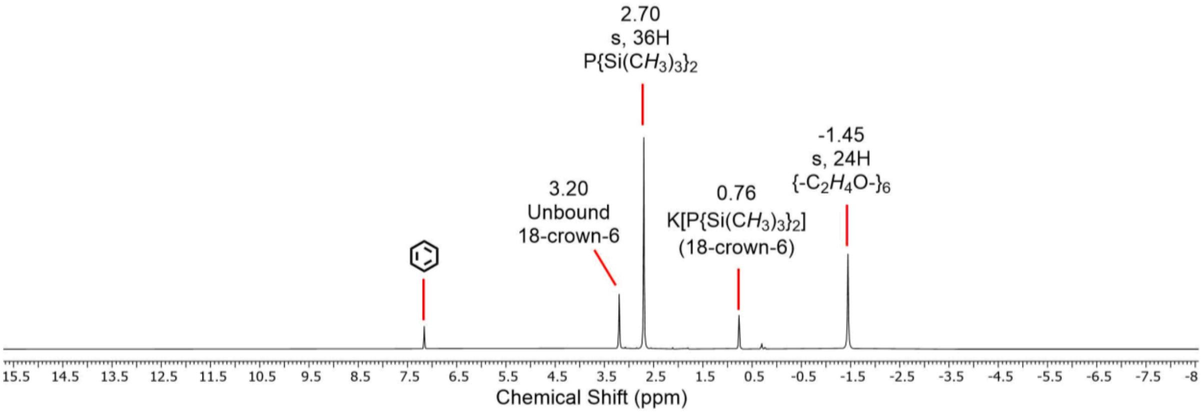

Figure S31. ^{1}H NMR spectrum (400 MHz) of **4-Sm** in C_6D_6. Expansion of spectrum of spectral width -100–100 ppm.

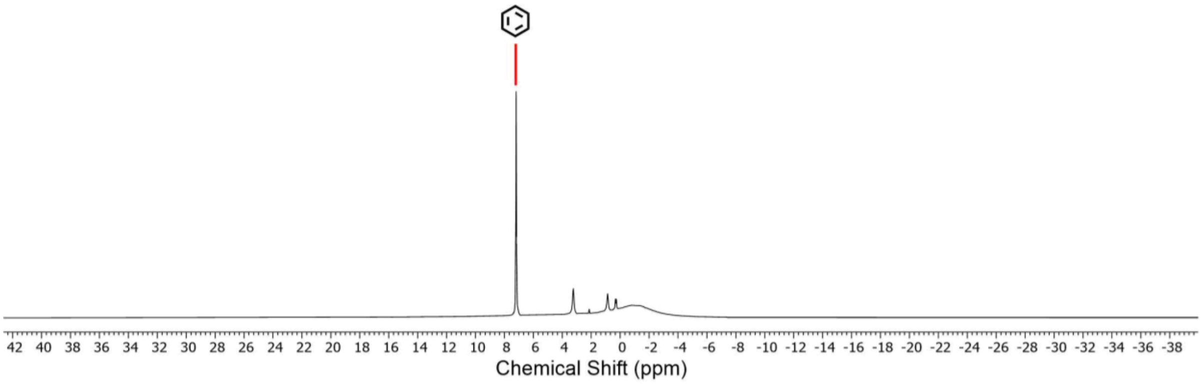

Figure S32. ^{1}H NMR spectrum (400 MHz) of **4-Eu** in C_6D_6. Expansion of spectrum of spectral width -100–100 ppm.

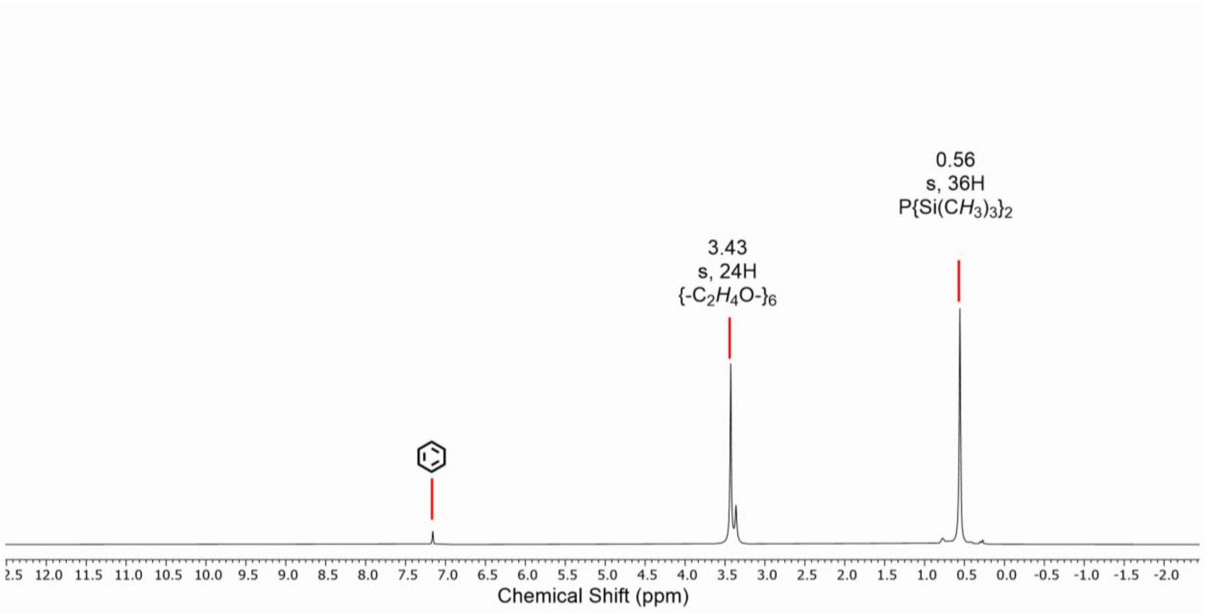

Figure S33. ^{1}H NMR spectrum (400 MHz) of **4-Yb** in C_6D_6.

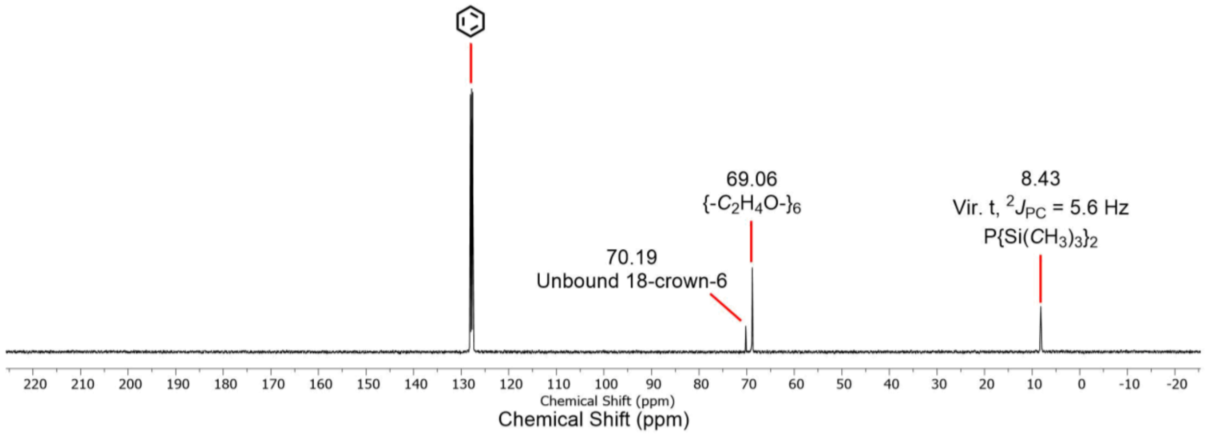

Figure S34. $^{13}C\{^1H\}$ NMR spectrum (101 MHz) of **4-Yb** in C_6D_6.

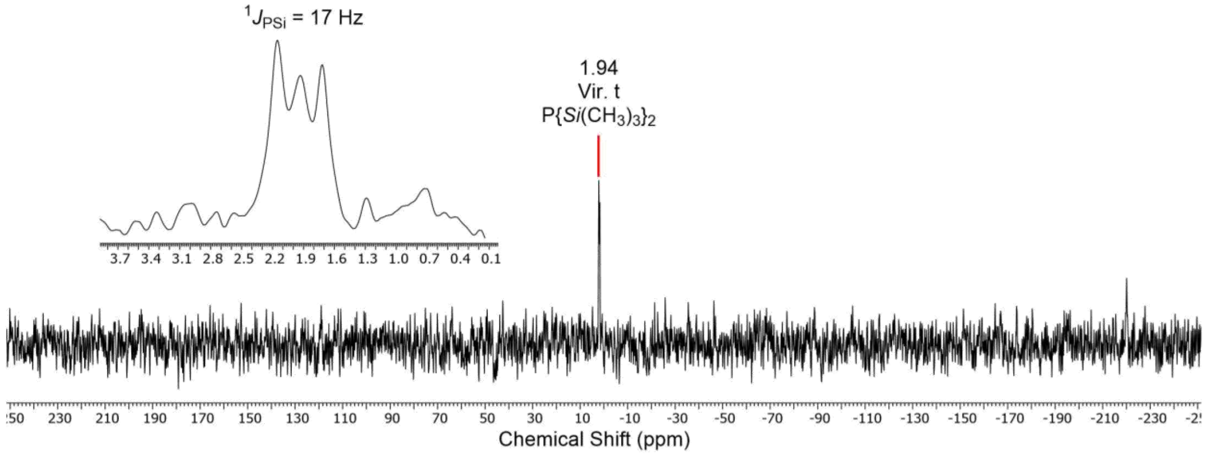

Figure S35. ^{29}Si DEPT90 NMR spectrum (79 MHz) of **4-Yb** in C_6D_6.

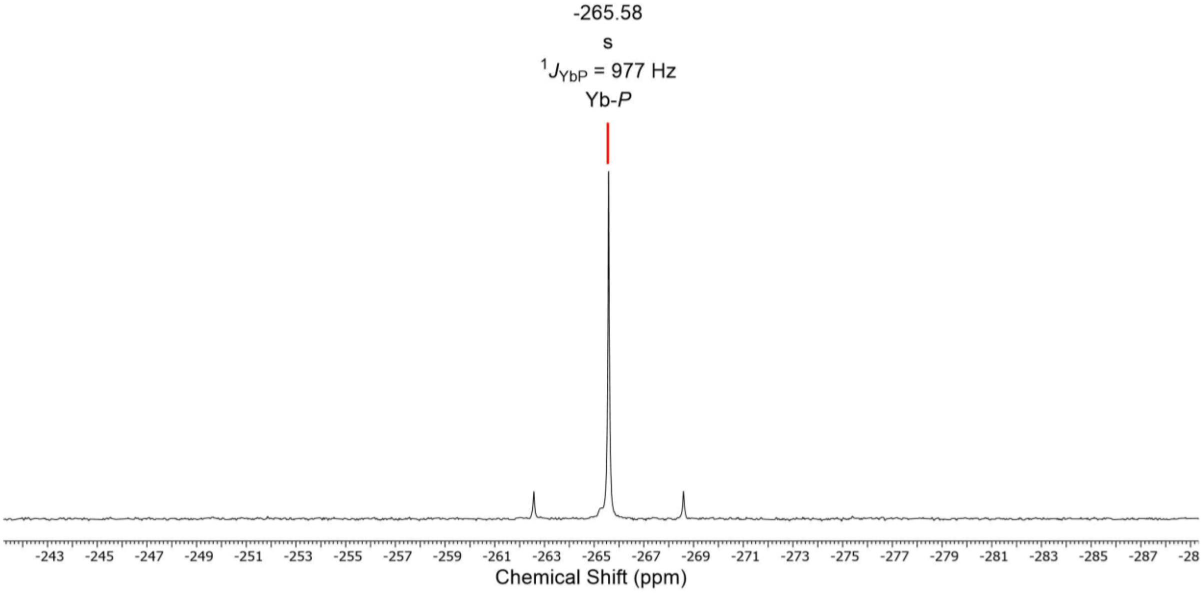

Figure S36. $^{31}P\{^{1}H\}$ NMR spectrum (162 MHz) of **4-Yb** in C_6D_6.

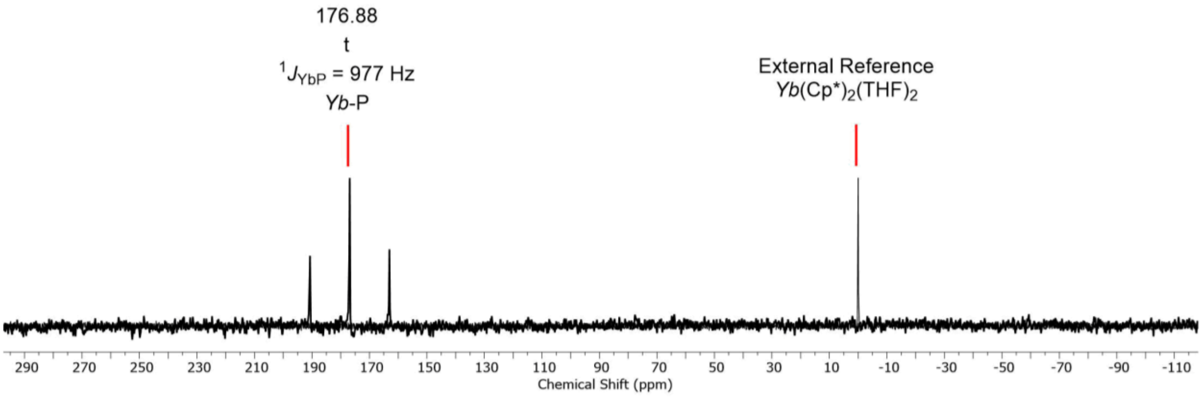

Figure S37. $^{171}Yb\{^{1}H\}$ NMR spectrum (71 MHz) of **4-Yb** in C_6D_6. Yb(Cp*)$_2$(THF)$_2$ reference shown in order to show relative chemical shift.

6. UV-Vis-NIR Spectroscopy

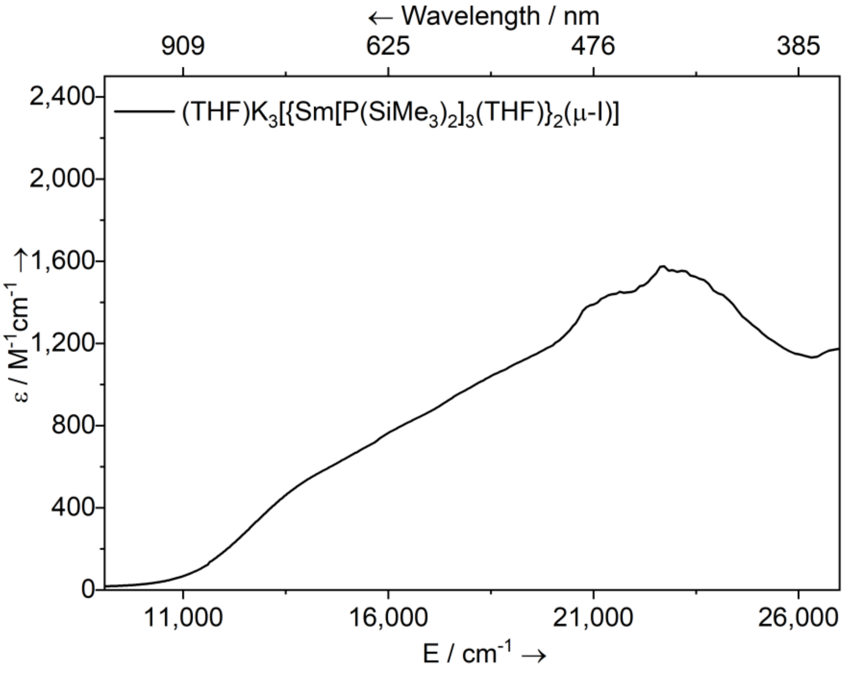

Figure S38. Electronic UV-Vis-NIR absorption spectrum of **1-Sm** in toluene (2 mM) between 9091–27000 cm^{-1} (1100–370 nm).

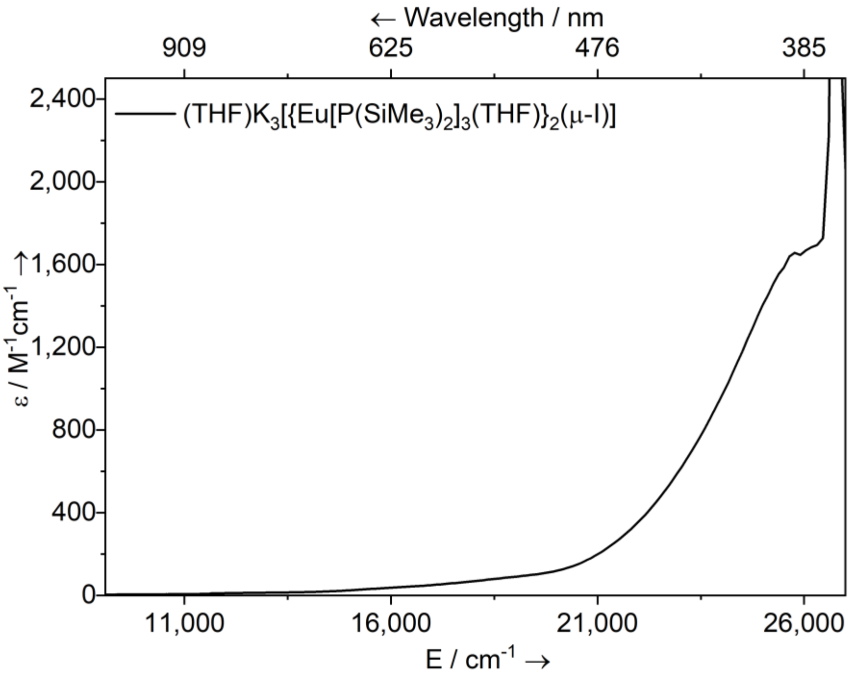

Figure S39. Electronic UV-Vis-NIR absorption spectrum of **1-Eu** in toluene (2 mM) between 9091–27000 cm^{-1} (1100– 370 nm).

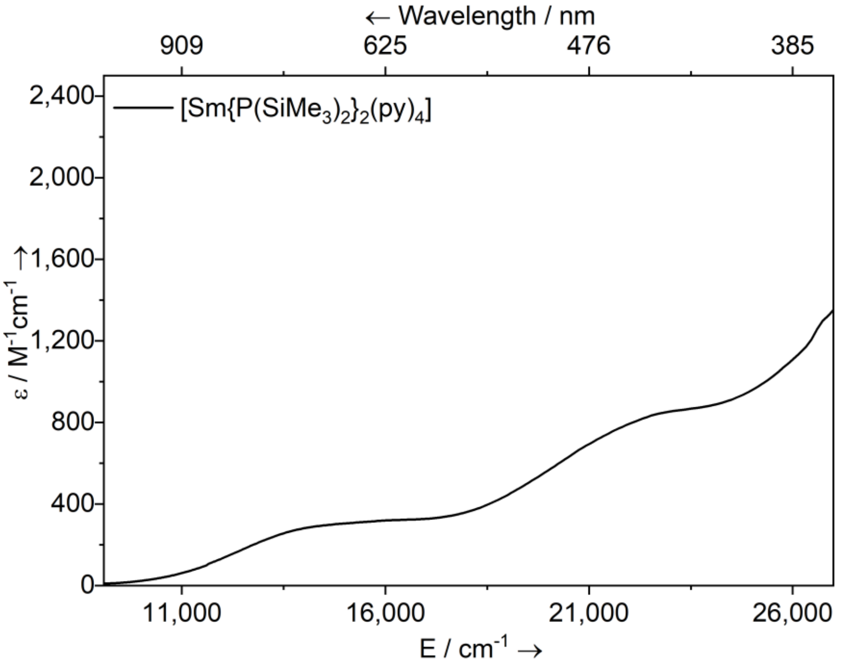

Figure S40. Electronic UV-Vis-NIR absorption spectrum of **3-Sm** in toluene (2 mM) between 9091–27000 cm^{-1} (1100–370 nm).

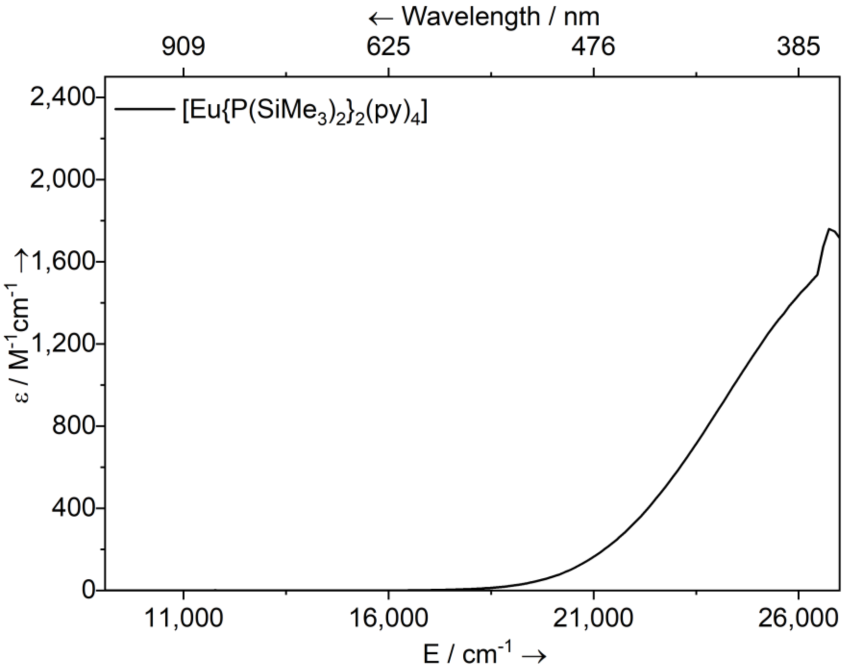

Figure S41. Electronic UV-Vis-NIR absorption spectrum of **3-Eu** in toluene (2 mM) between 9091–27000 cm^{-1} (1100–370 nm).

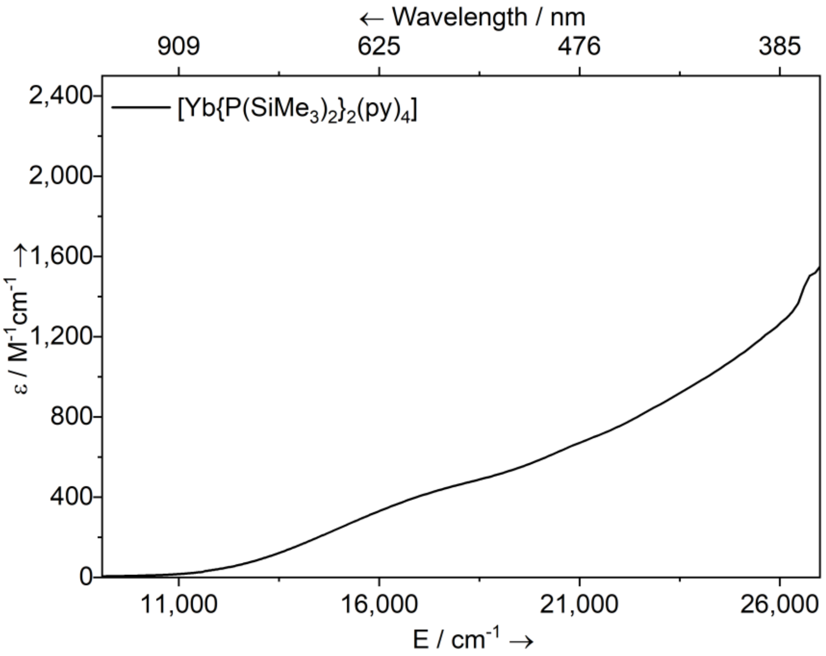

Figure S42. Electronic UV-Vis-NIR absorption spectrum of **3-Yb** in toluene (2 mM) between 9091–27000 cm^{-1} (1100–370 nm).

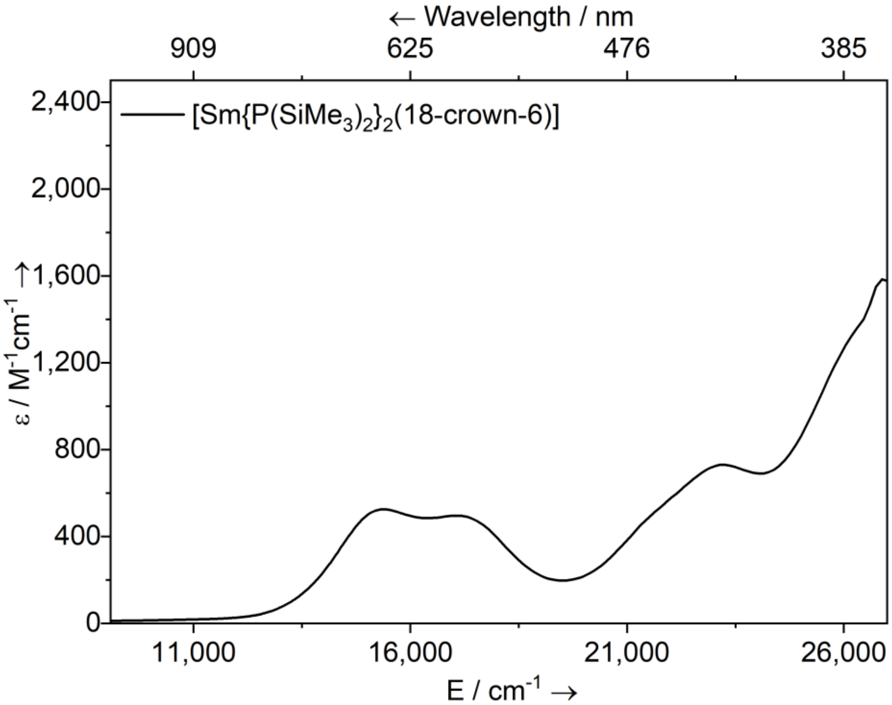

Figure S43. Electronic UV-Vis-NIR absorption spectrum of **4-Sm** in toluene (2 mM) between 9091–27000 cm^{-1} (1100– 370 nm).

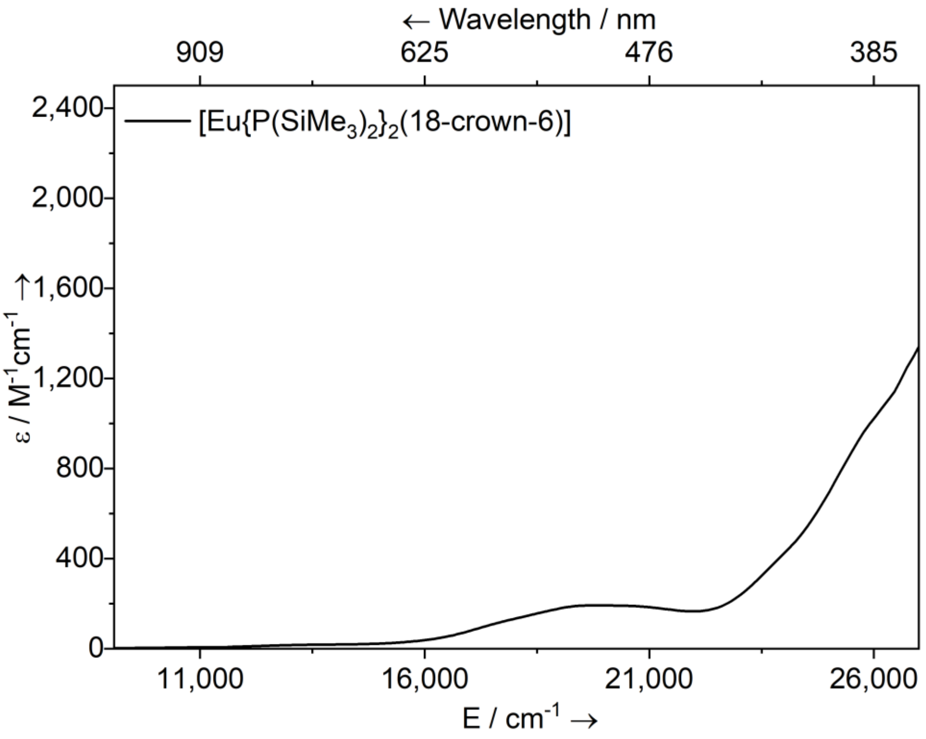

Figure S44. Electronic UV-Vis-NIR absorption spectrum of **4-Eu** in toluene (2 mM) between 9091–27000 cm^{-1} (1100– 370 nm).

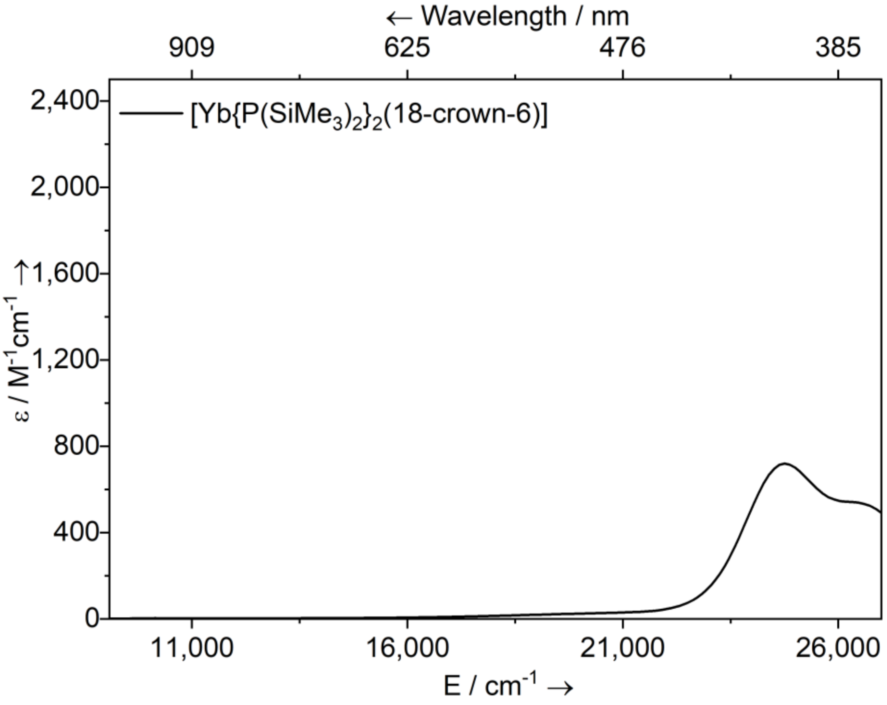

Figure S45. Electronic UV-Vis-NIR absorption spectrum of **4-Yb** in toluene (2 mM) between 9091–27000 cm^{-1} (1100– 370 nm).

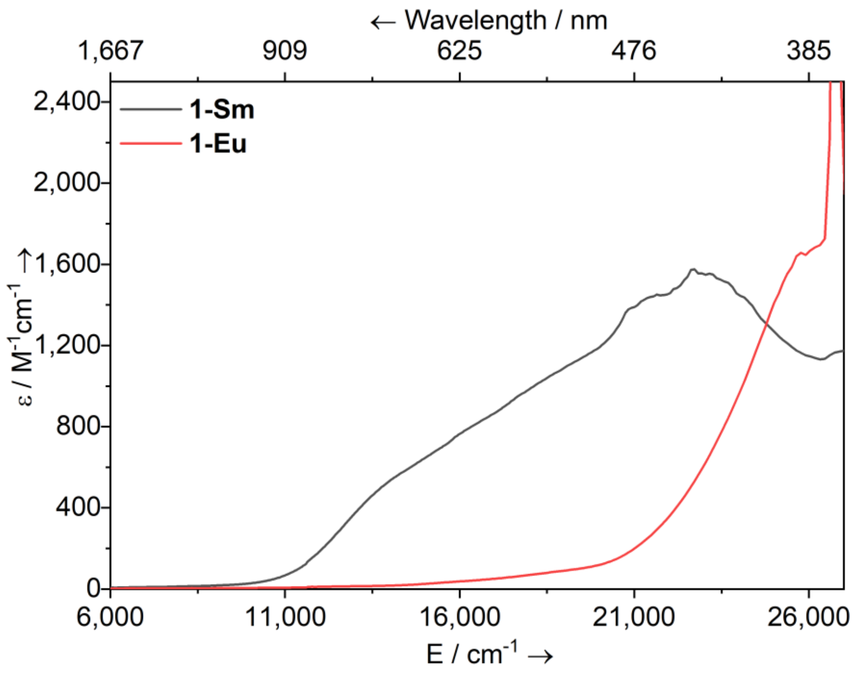

Figure S46 Overlaid electronic UV-Vis-NIR absorption spectra of **1-Ln** in toluene (2 mM) between 6000–27000 cm^{-1} (1100– 370 nm).

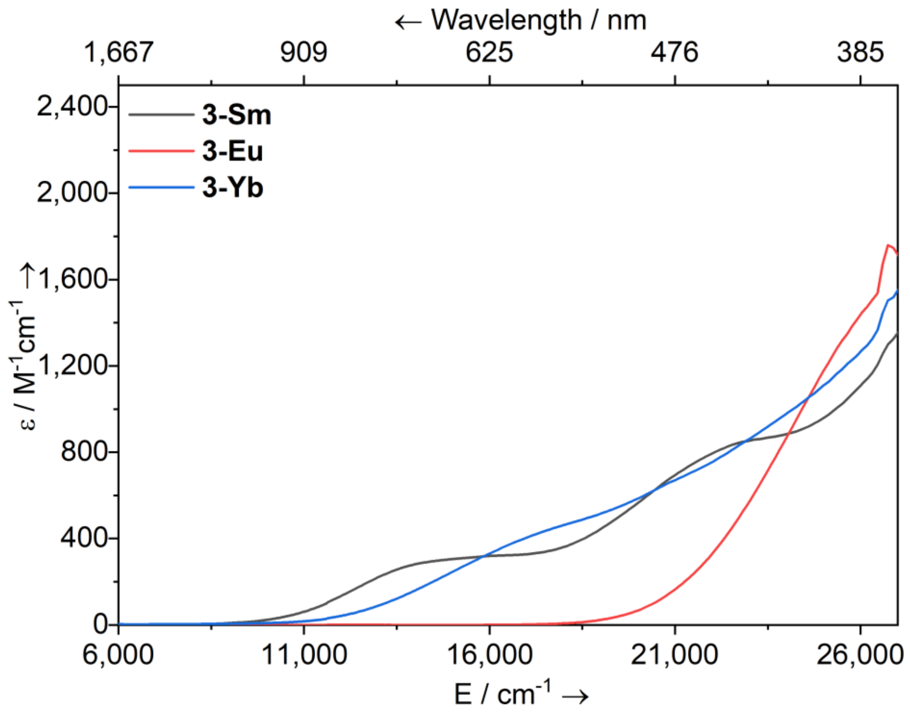

Figure S47. Overlaid electronic UV-Vis-NIR absorption spectra of **3-Ln** in toluene (2 mM) between 9091–27000 cm^{-1} (1100– 370 nm).

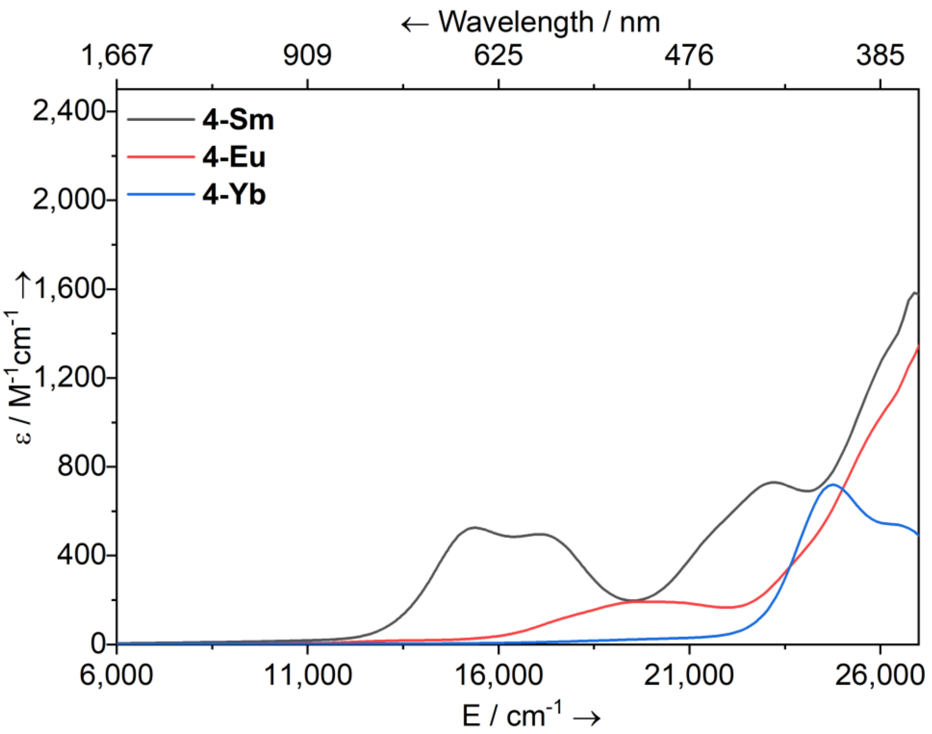

Figure S48. Overlaid electronic UV-Vis-NIR absorption spectra of **4-Ln** in toluene (2 mM) between 9091–27000 cm^{-1} (1100– 370 nm).

7. Photoluminescence Properties: 1-Eu, 3-Eu, 3-Yb, 4-Eu, 4-Yb

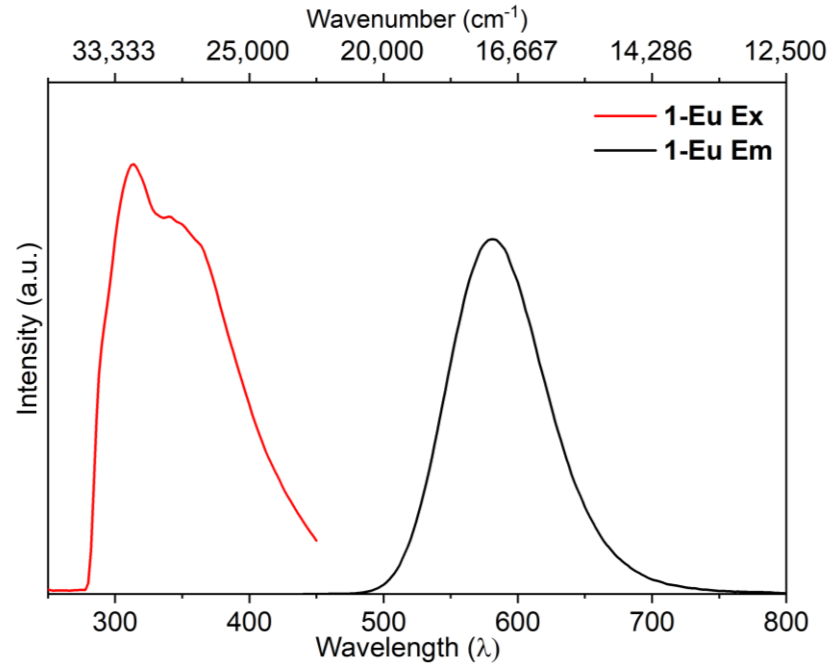

Figure S49. Emission spectrum (**Em**), black trace and excitation spectrum (**Ex**), red trace of complex **1-Eu** in toluene (0.508 mM). Excited at 315 nm (Ex), observed at 579 nm (Em).

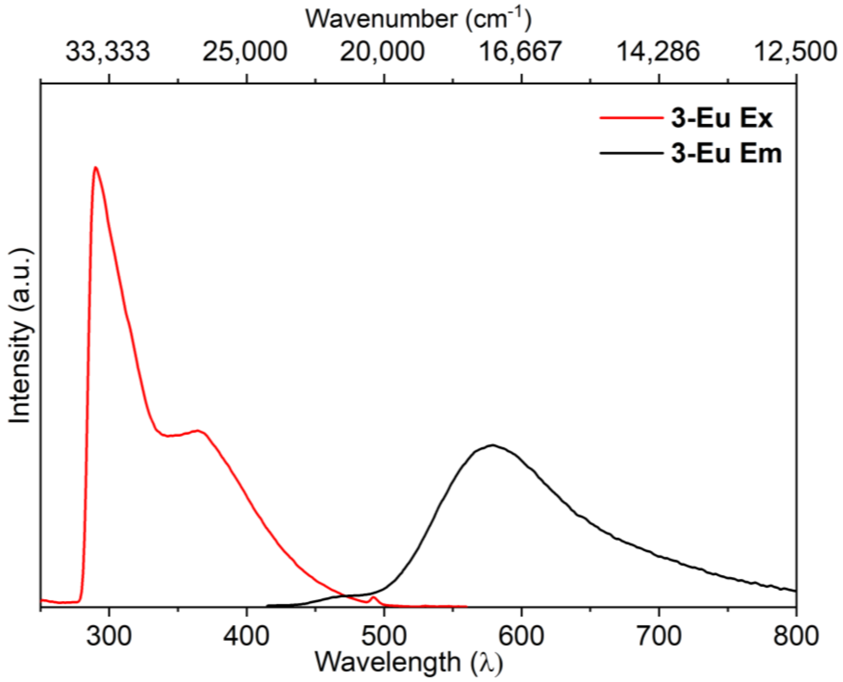

Figure S50. Emission spectrum (**Em**), black trace and excitation spectrum (**Ex**), red trace of complex **3-Eu** in toluene (0.111 mM). Excited at 290 nm (Ex), observed at 579 nm (Em).

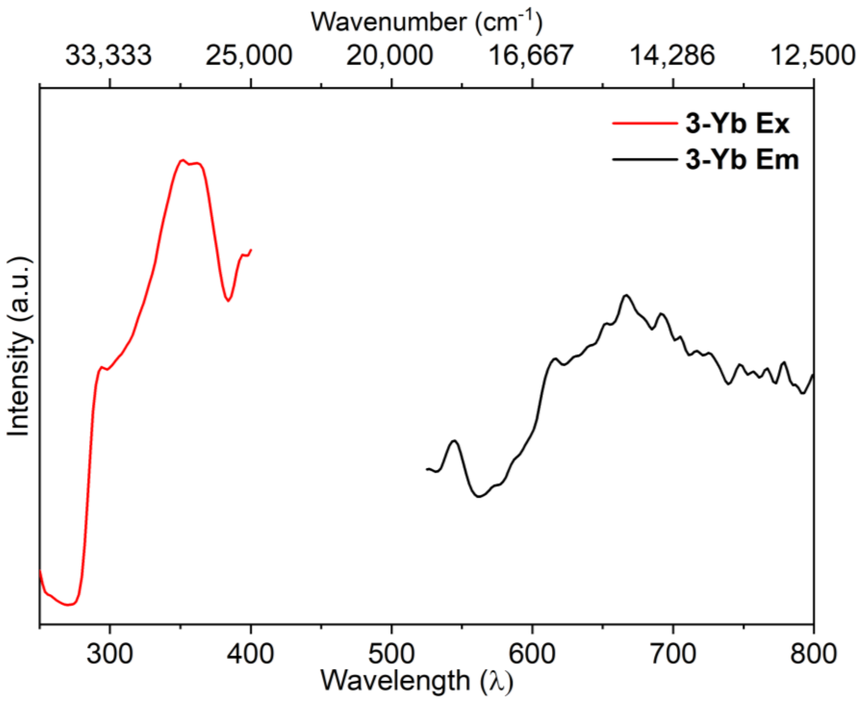

Figure S51. Emission spectrum (**Em**), black trace and excitation spectrum (**Ex**), red trace of complex **3-Yb** in toluene (0.177 mM). Excited at 350 nm (Ex), observed at 665 nm (Em).

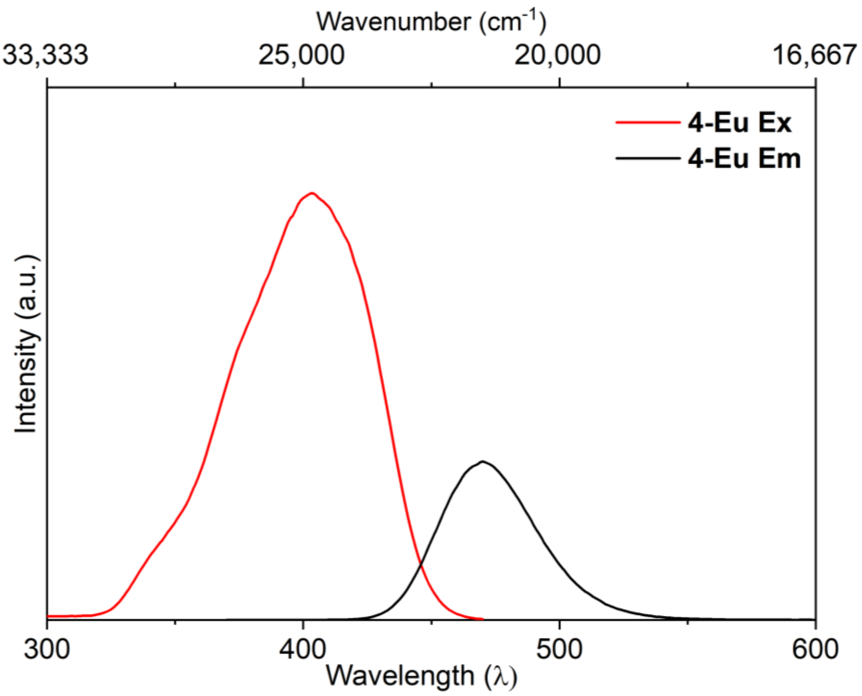

Figure S52. Emission spectrum (**Em**), black trace and excitation spectrum (**Ex**), red trace of complex **4-Eu** in toluene (1.6 mM). Excited at 405 nm (Ex), observed at 475 nm (Em).

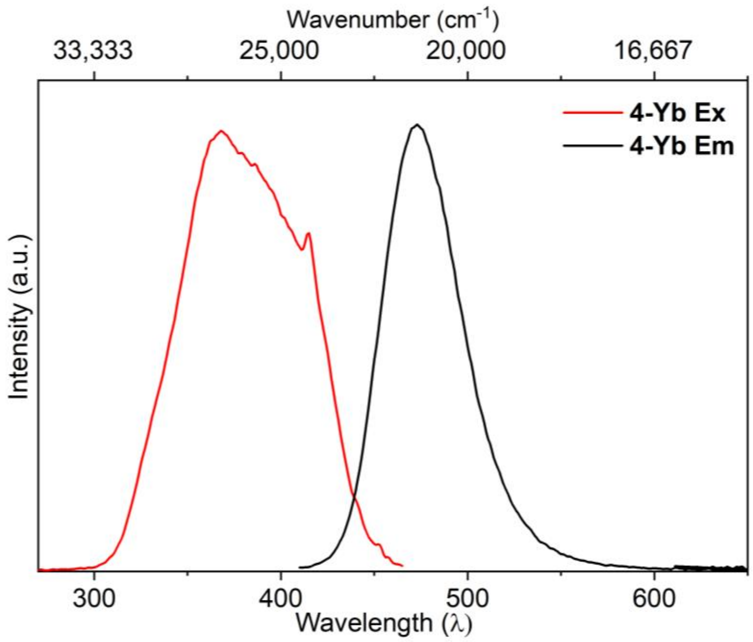

Figure S53. Emission spectrum (**Em**), black trace and excitation spectrum (**Ex**), red trace of complex **4-Yb** in toluene (1.58 mM). Excited at 370 nm (Ex), observed at 475 nm (Em).

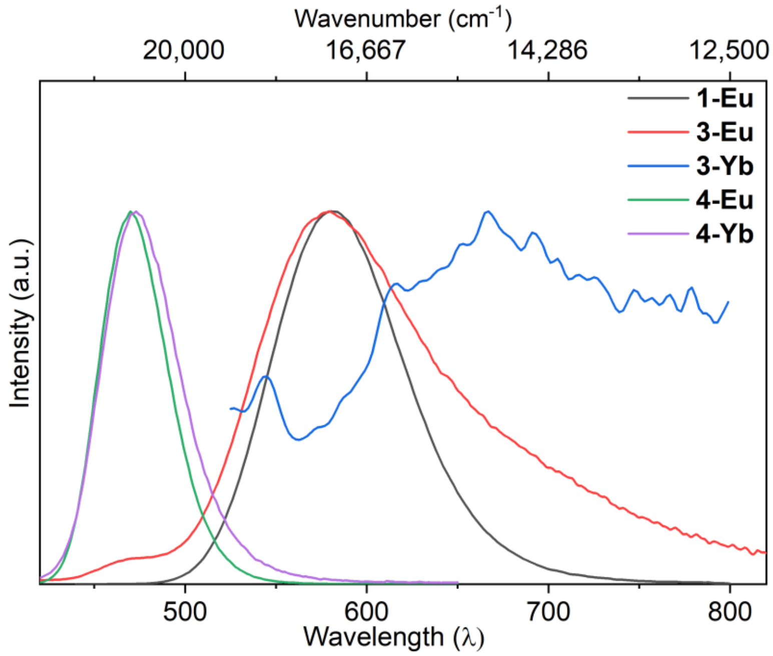

Figure S54. Overlaid normalized emission spectra (**Em**) of complexes **1-Eu**, **3-Eu**, **3-Yb**, **4-Eu** and **4-Yb** in toluene.

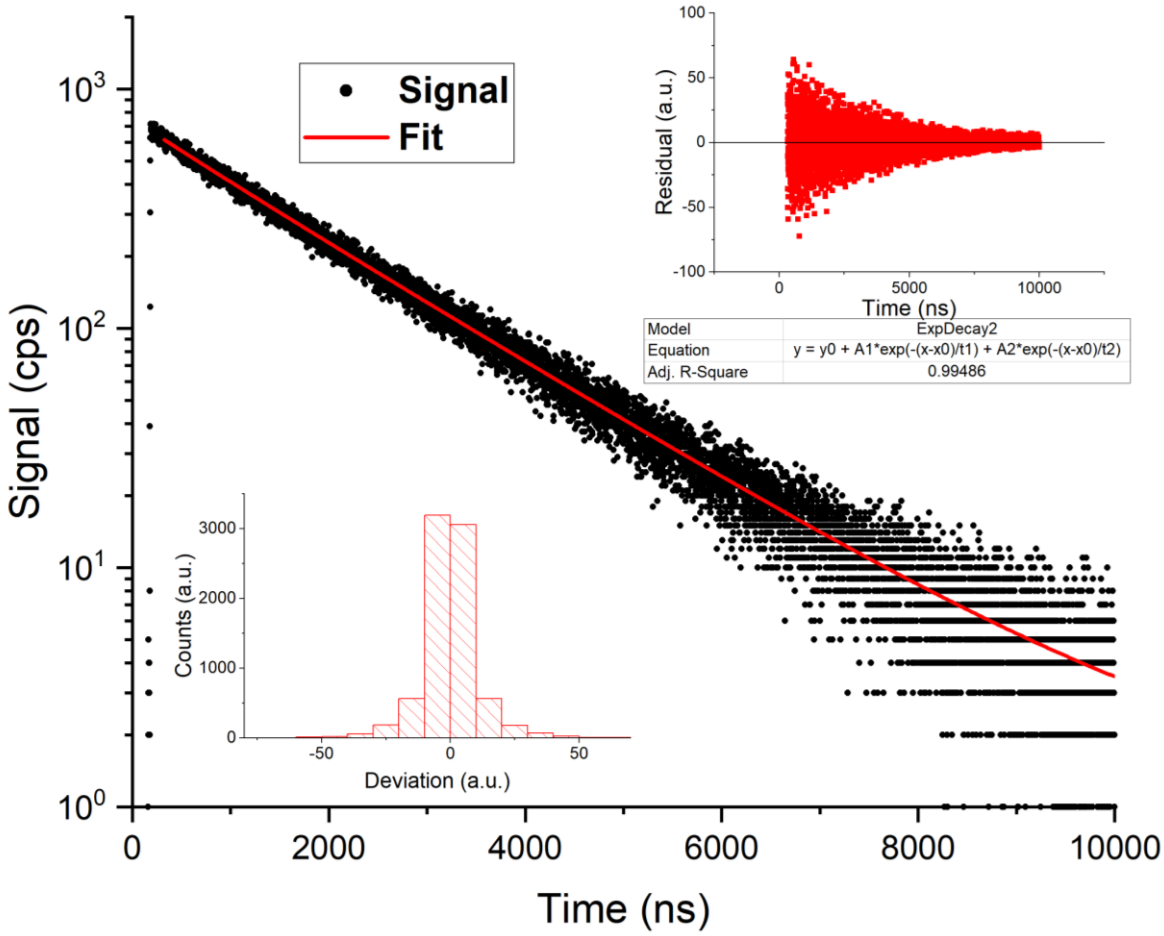

Figure S55. Luminescence decay profile (black) of the emission signal in **1-Eu** with corresponding bi-exponential decay fit (red) (λ_{ex} = 375 nm, λ_{em} = 579 nm T = 293 K). Inset: associated residuals of the fit showing both normal distribution (bottom left) and error variance (top right).

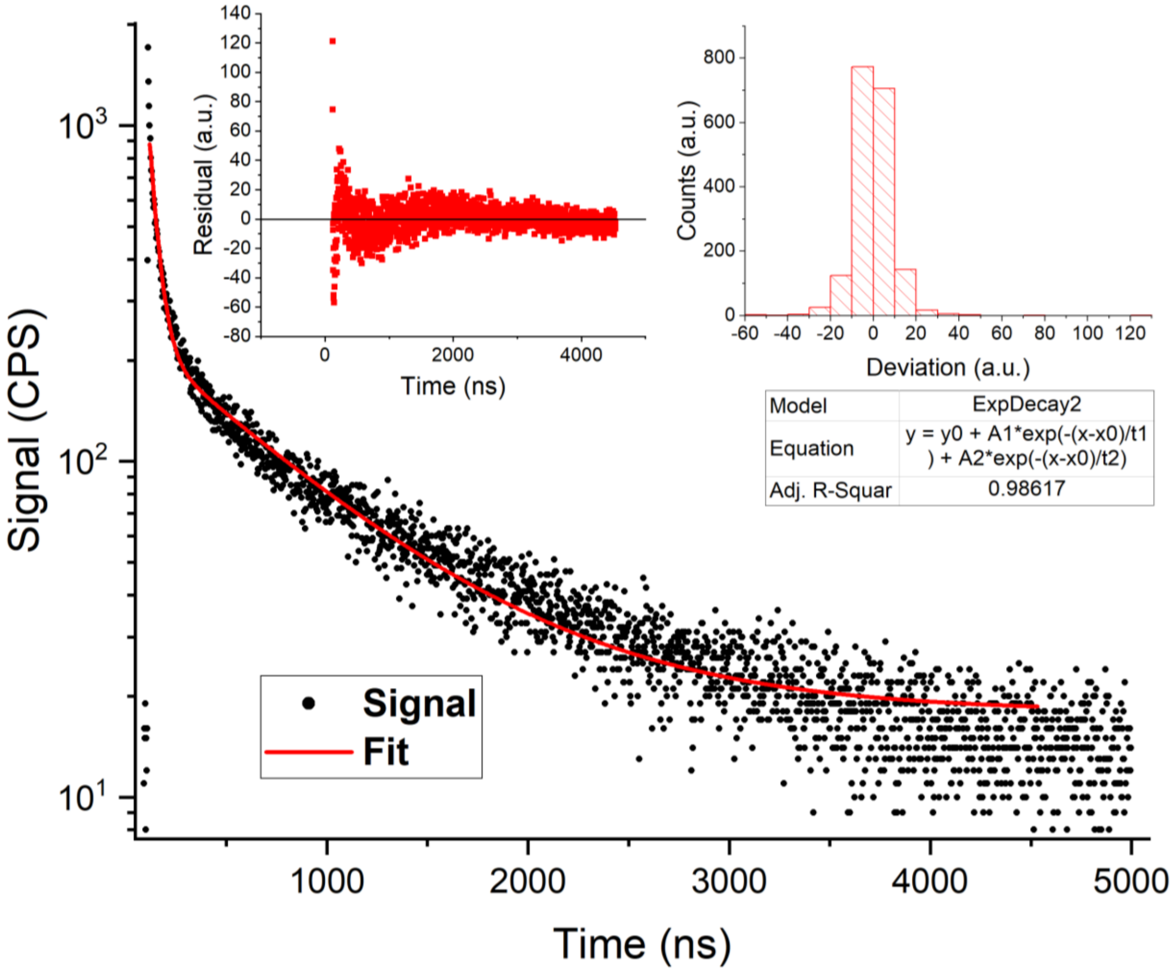

Figure S56. Luminescence decay profile (black) of the emission signal in **3-Eu** with corresponding bi-exponential decay fit (red) (λ_{ex} = 375 nm, λ_{em} = 579 nm T = 293 K). Inset: associated residuals of the fit showing both normal distribution (top right) and error variance (top left).

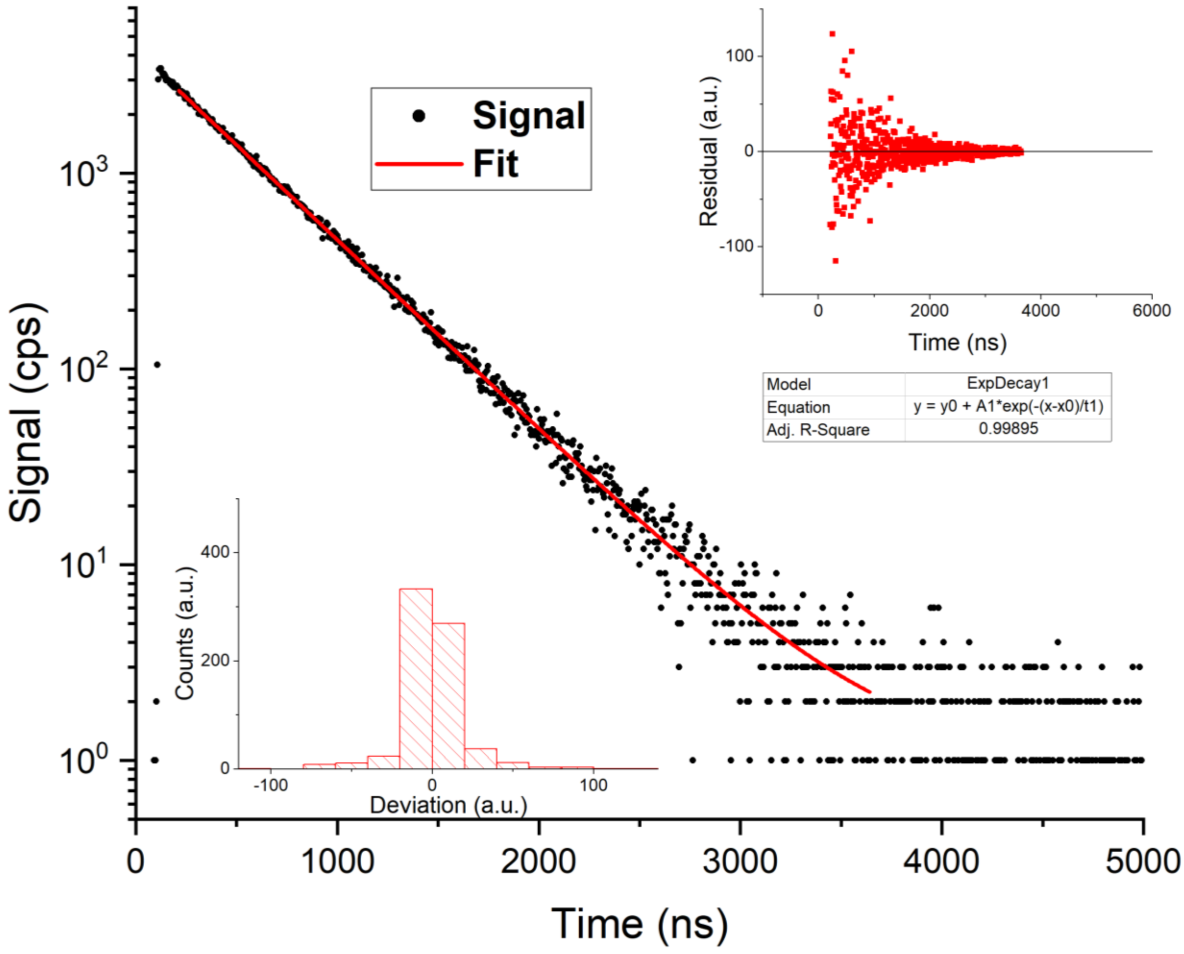

Figure S57. Luminescence decay profile (black) of the emission signal in **4-Eu** with corresponding mono-exponential decay fit (red) (λ_{ex} = 375 nm, λ_{em} = 475 nm T = 293 K). Inset: associated residuals of the fit showing both normal distribution (top right) and error variance (top left).

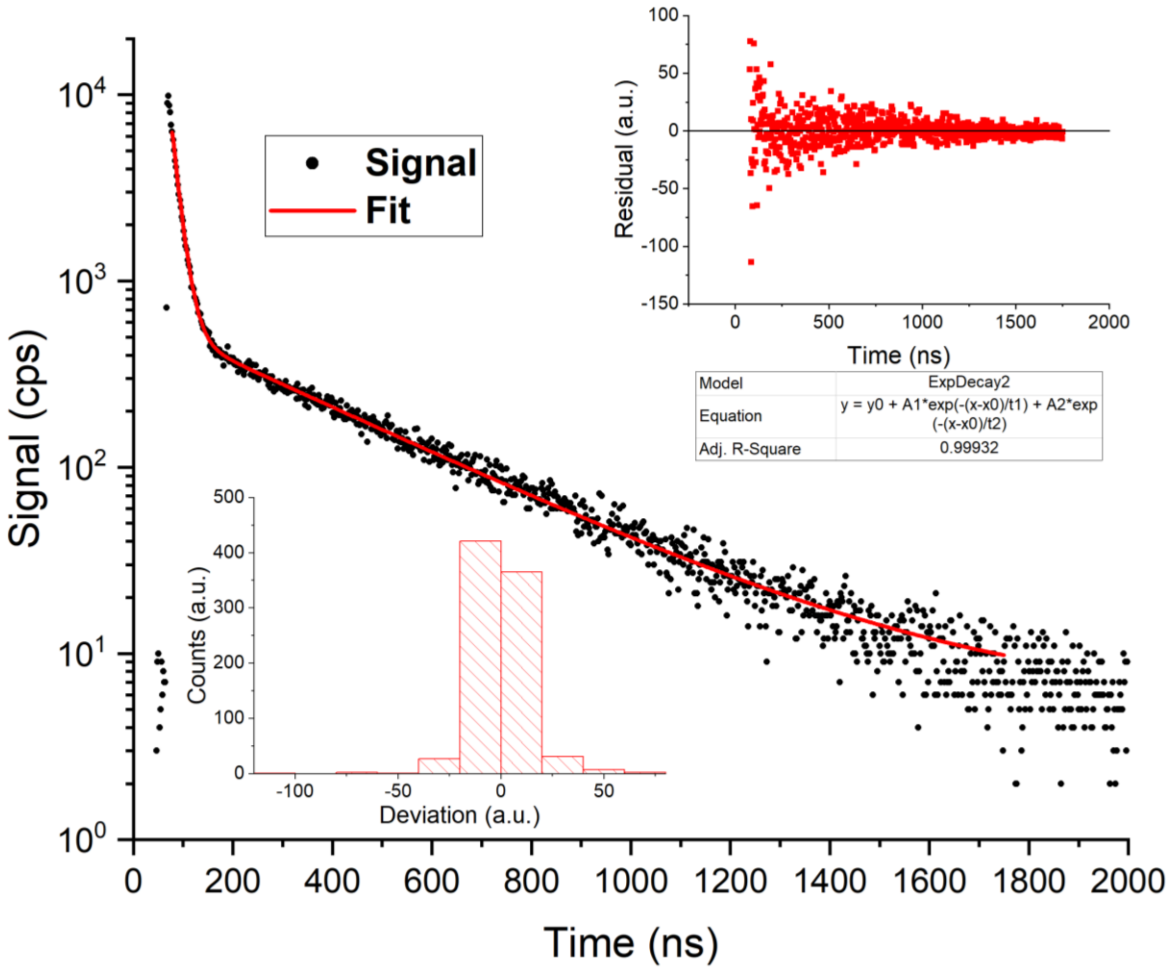

Figure S58. Luminescence decay profile (black) of the emission signal in **4-Yb** with corresponding bi-exponential decay fit (red) (λ_{ex} = 375 nm, λ_{em} = 475 nm T = 293 K). Inset: associated residuals of the fit showing both normal distribution (top right) and error variance (top left).

8. SQUID Magnetometry: 1-Sm, 1-Eu, 3-Sm, 3-Eu, 4-Sm, 4-Eu

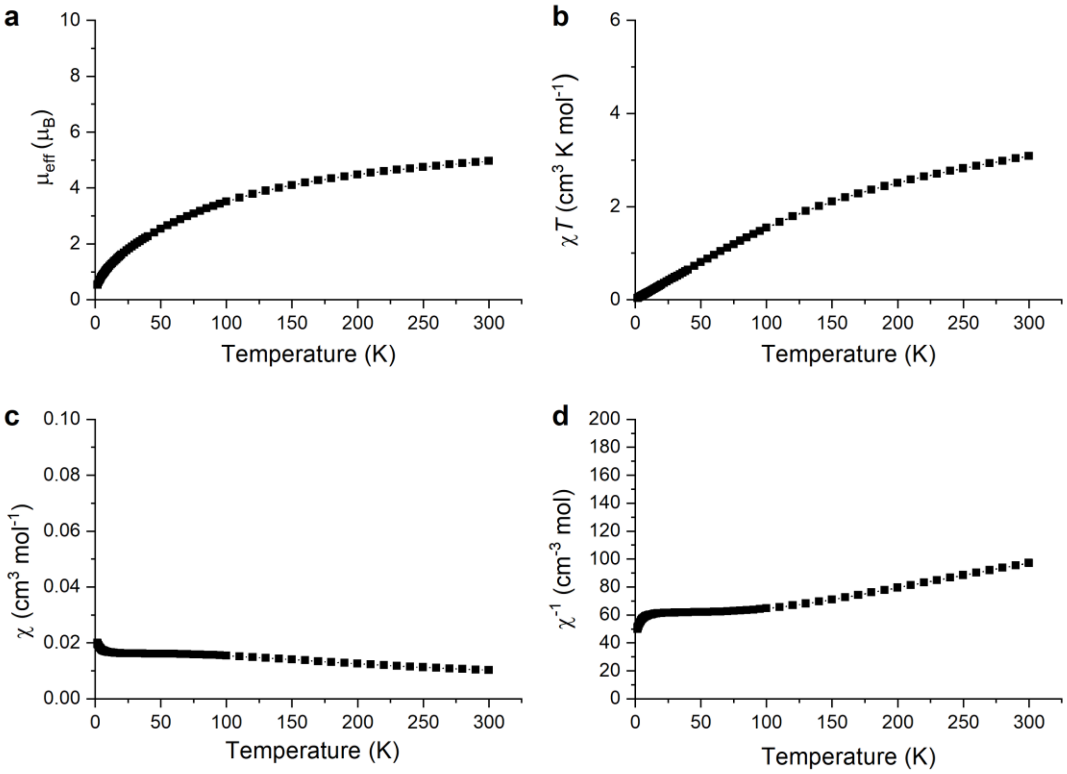

Figure S59. Variable-temperature SQUID magnetic data for powdered **1-Sm** in a 0.5 T applied magnetic field, presented as: **a** – μ_{eff} *vs*. T; **b** – χT *vs*. T; **c** – χ *vs*. T; **d** – χ^{-1} *vs*. T.

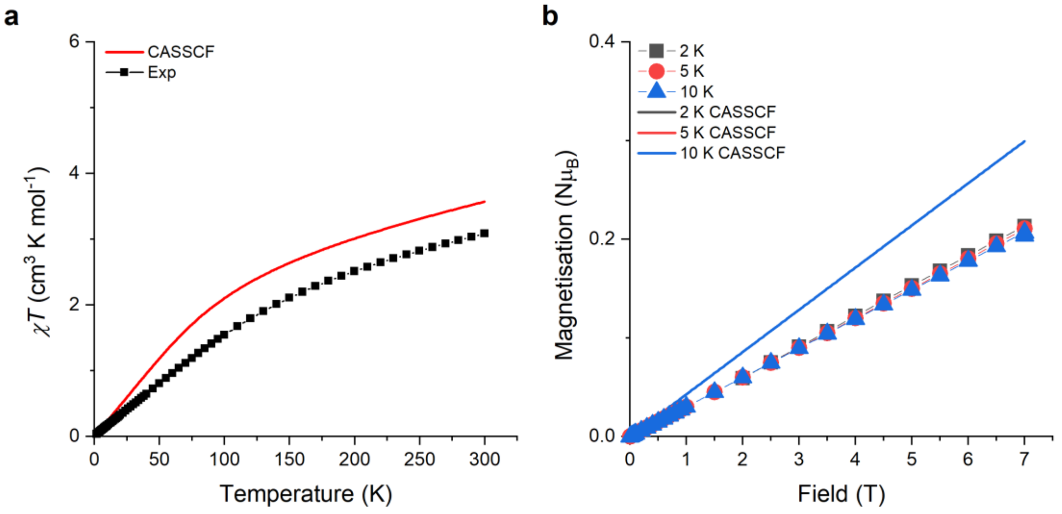

Figure S60. a – Magnetic susceptibility plot and **b** – Magnetization *vs*. field plot for **1-Sm**. Solid lines show CASSCF results.

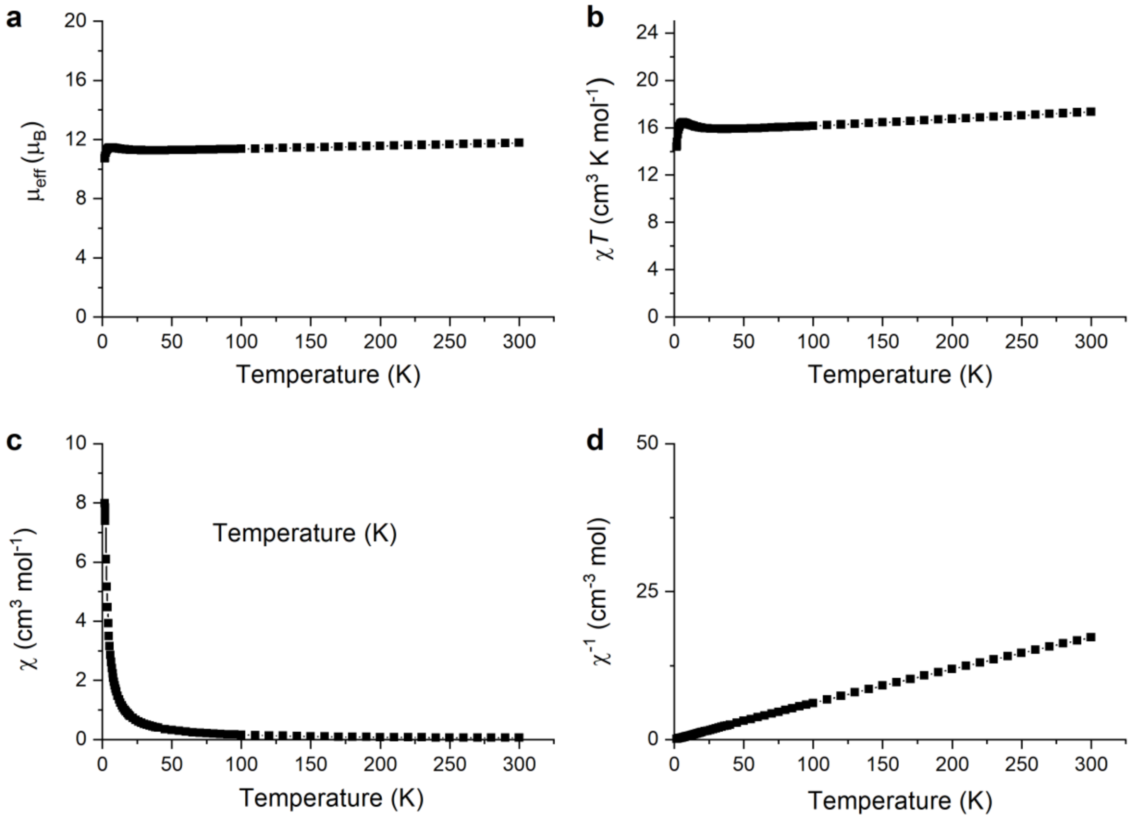

Figure S61. Variable-temperature SQUID magnetic data for powdered **1-Eu** in a 0.1 T applied magnetic field, presented as: **a** – μ_{eff} *vs*. T; **b** – χT *vs*. T; **c** – χ *vs*. T; **d** – χ^{-1} *vs*. T.

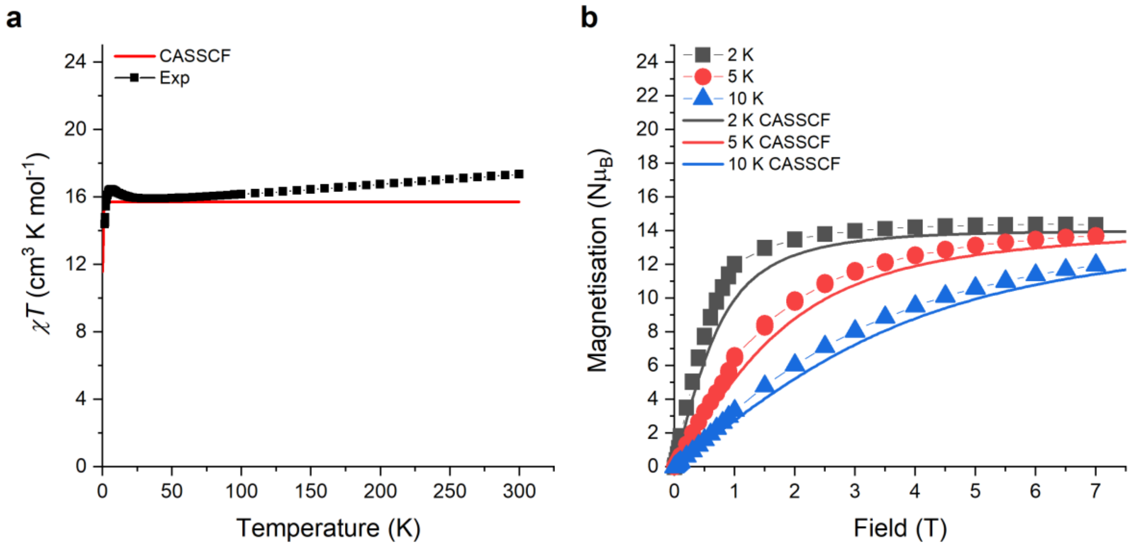

Figure S62. a – Magnetic susceptibility plot and **b** – Magnetization *vs*. field plot for **1-Eu**. Solid lines show CASSCF results. CASSCF results for magnetization are ongoing.

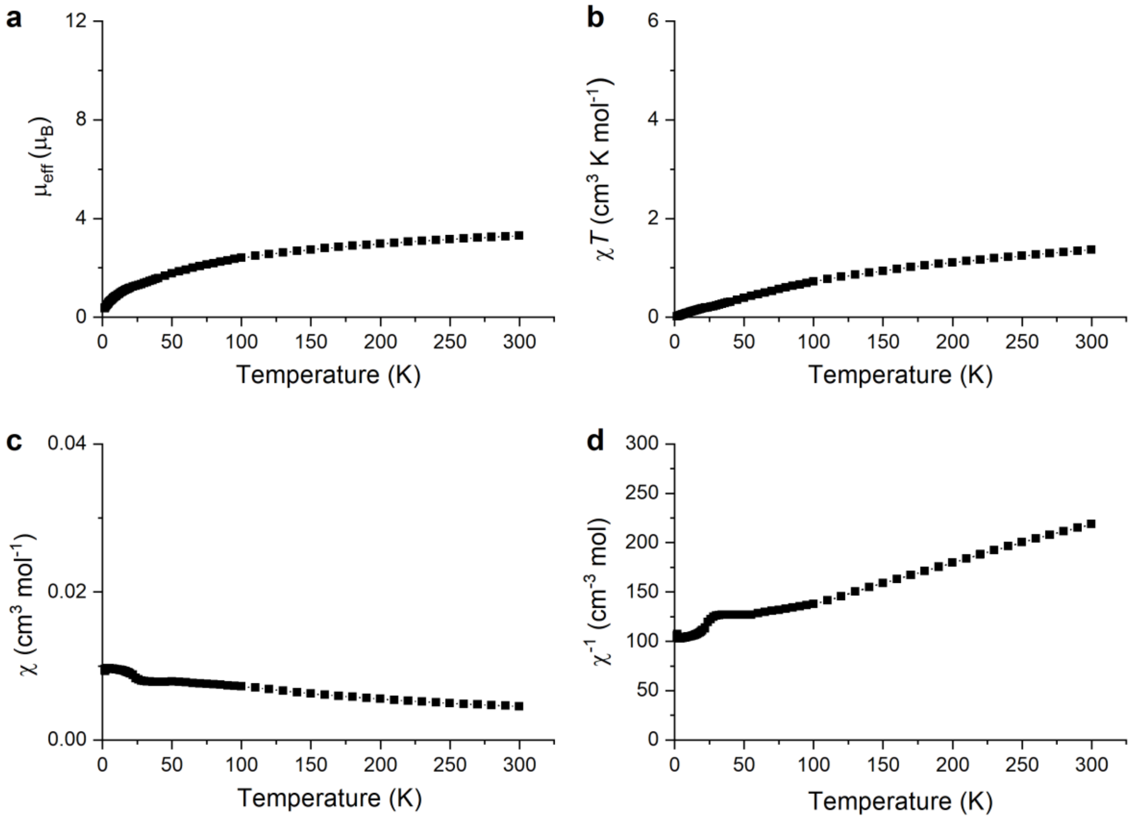

Figure S63. Variable-temperature SQUID magnetic data for powdered **3-Sm** in a 0.1 T applied magnetic field, presented as: **a** – μ_{eff} *vs*. T; **b** – χT *vs*. T; **c** – χ *vs*. T; **d** – χ^{-1} *vs*. T.

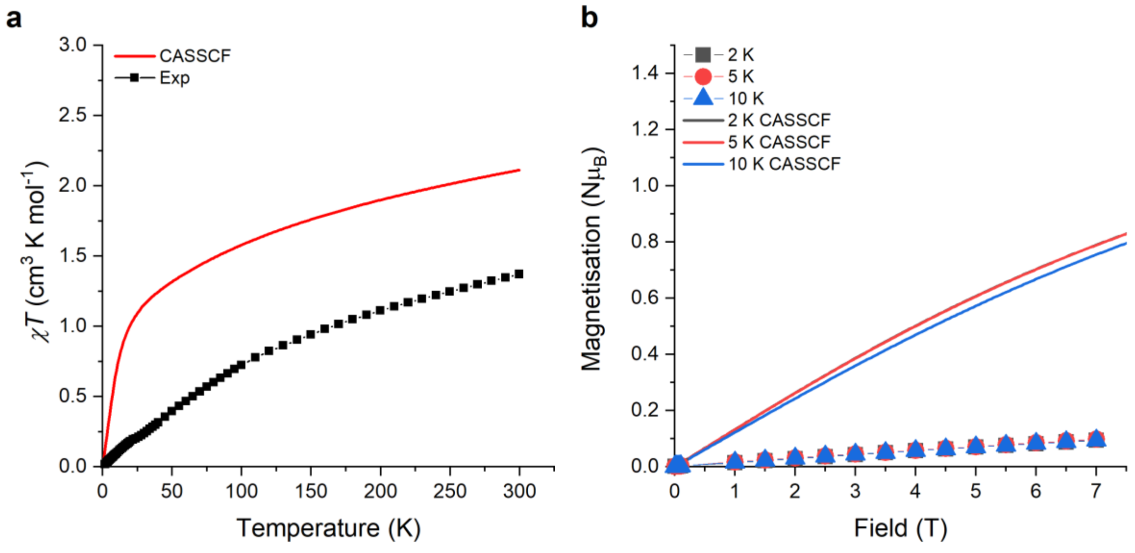

Figure S64. a – Magnetic susceptibility plot and **b** – Magnetization *vs*. field plot for **3-Sm**. Solid lines show CASSCF results.

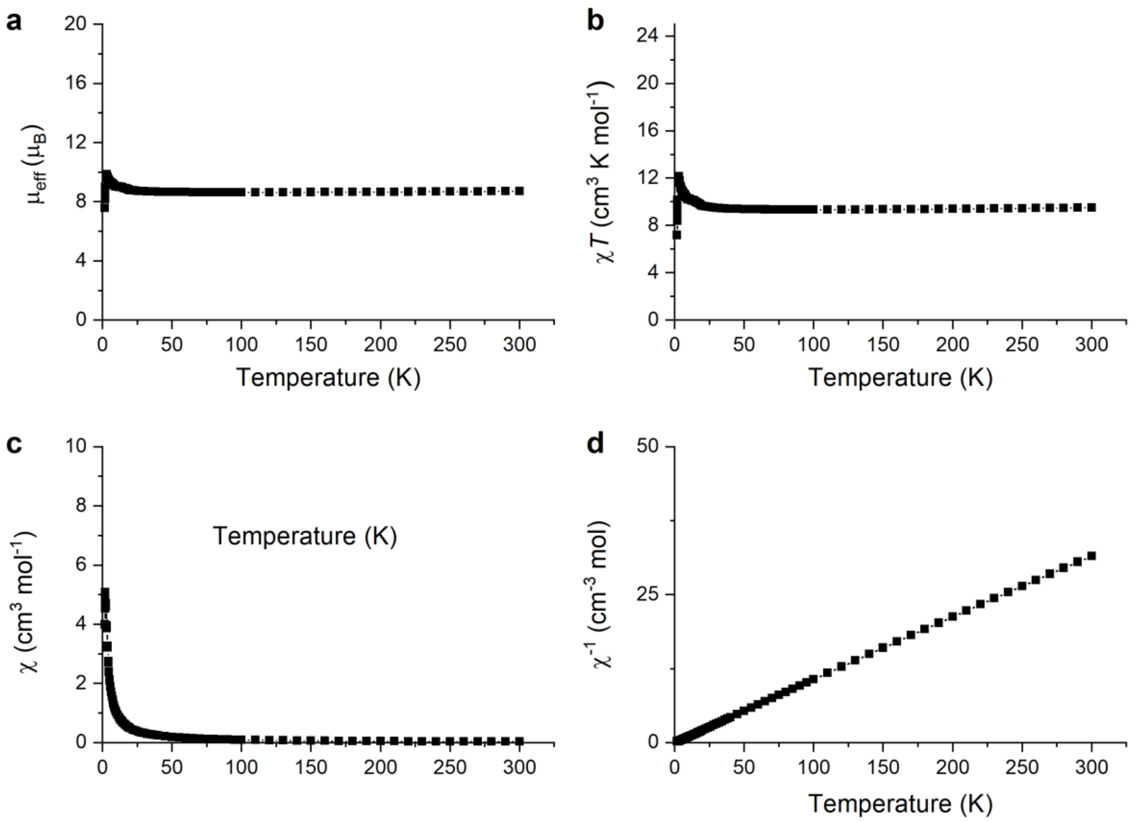

Figure S65. Variable-temperature SQUID magnetic data for powdered **3-Eu** in a 0.1 T applied magnetic field, presented as: **a** – μ_{eff} *vs*. T; **b** – χT *vs*. T; **c** – χ *vs*. T; **d** – χ^{-1} *vs*. T.

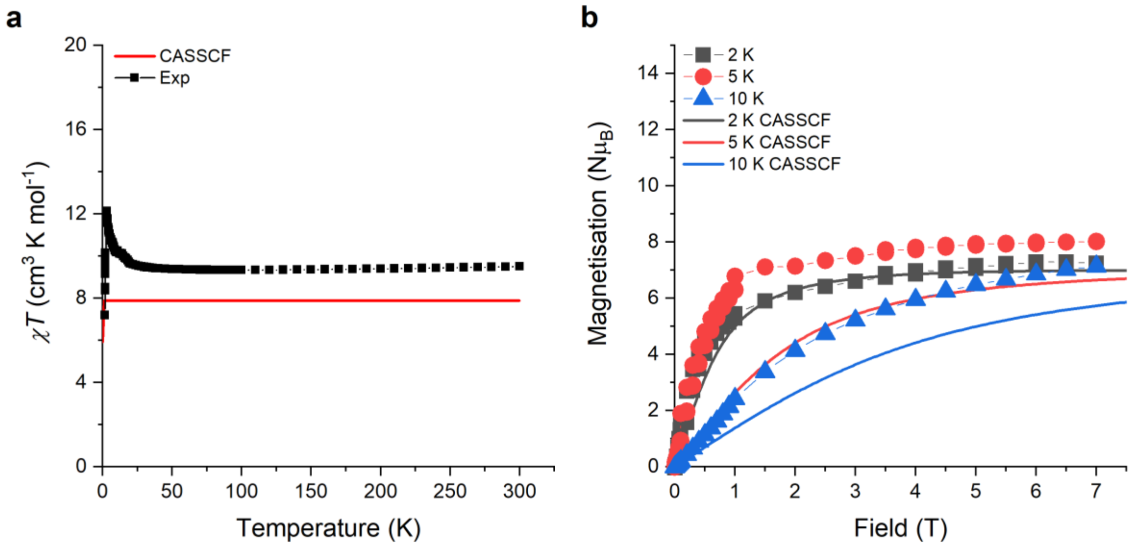

Figure S66. a – Magnetic susceptibility plot and **b** – Magnetization *vs*. field plot for **3-Eu**. Solid lines show CASSCF results.

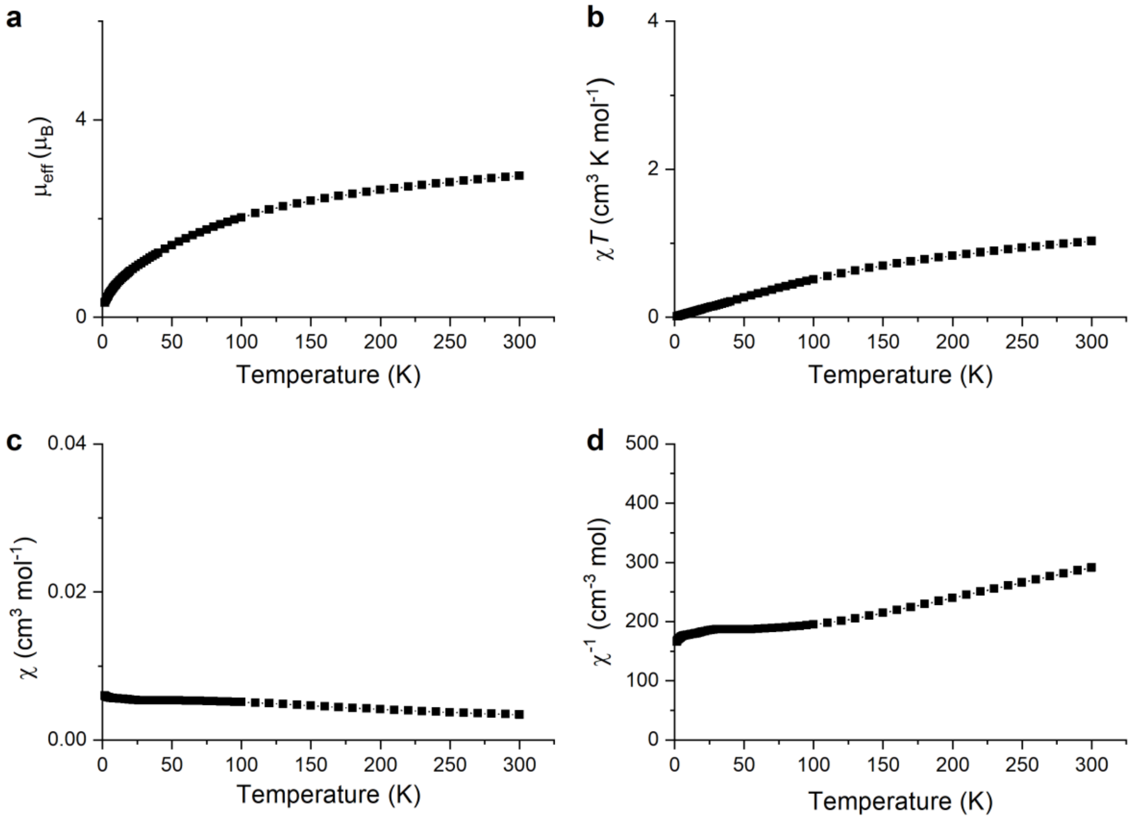

Figure S67. Variable-temperature SQUID magnetic data for powdered **4-Sm** in a 0.1 T applied magnetic field, presented as: **a** – μ_{eff} *vs*. T; **b** – χT *vs*. T; **c** – χ *vs*. T; **d** – χ^{-1} *vs*. T.

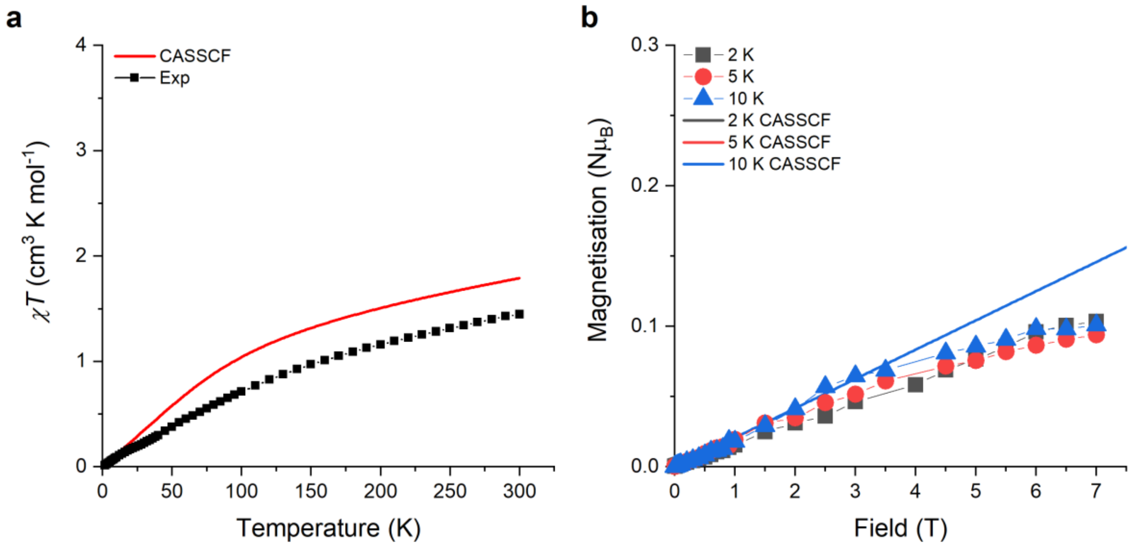

Figure S68. a – Magnetic susceptibility plot and **b** – Magnetization *vs*. field plot for **4-Sm**. Solid lines show CASSCF results.

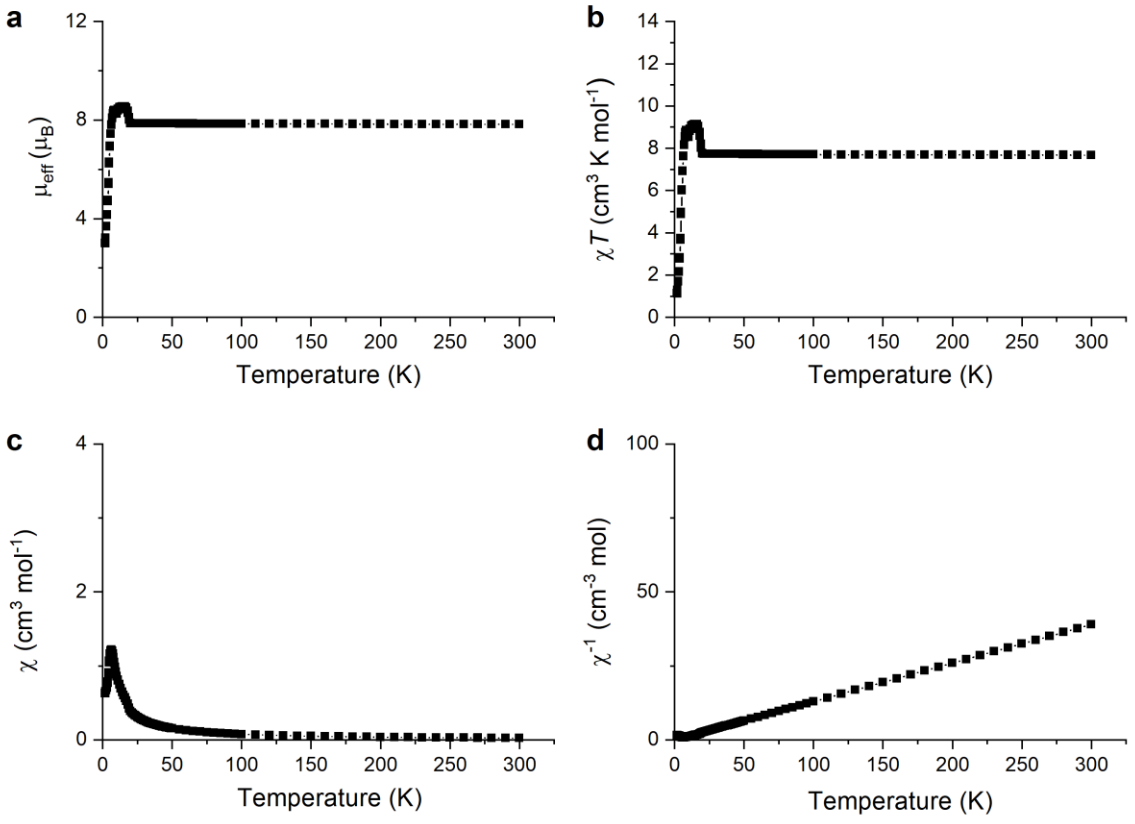

Figure S69. Variable-temperature SQUID magnetic data for powdered **4-Eu** in a 0.1 T applied magnetic field, presented as: **a** – μ_{eff} *vs*. T; **b** – χT *vs*. T; **c** – χ *vs*. T; **d** – χ^{-1} *vs*. T.

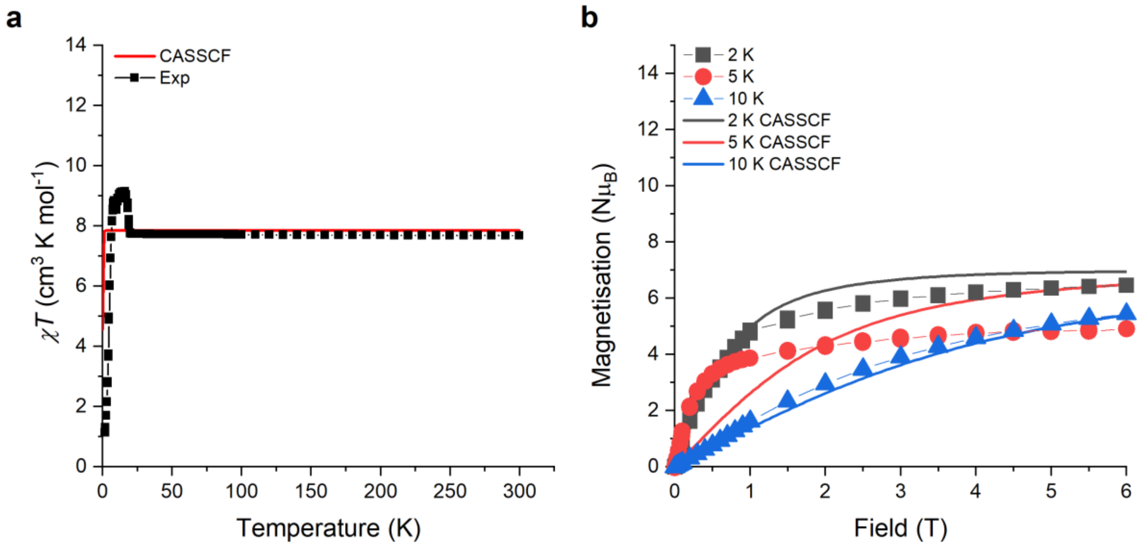

Figure S70. a – Magnetic susceptibility plot and **b** – Magnetization *vs*. field plot for **4-Eu**. Solid lines show CASSCF results.

9. EPR Spectroscopy: 1-Eu, 3-Eu, 4-Eu

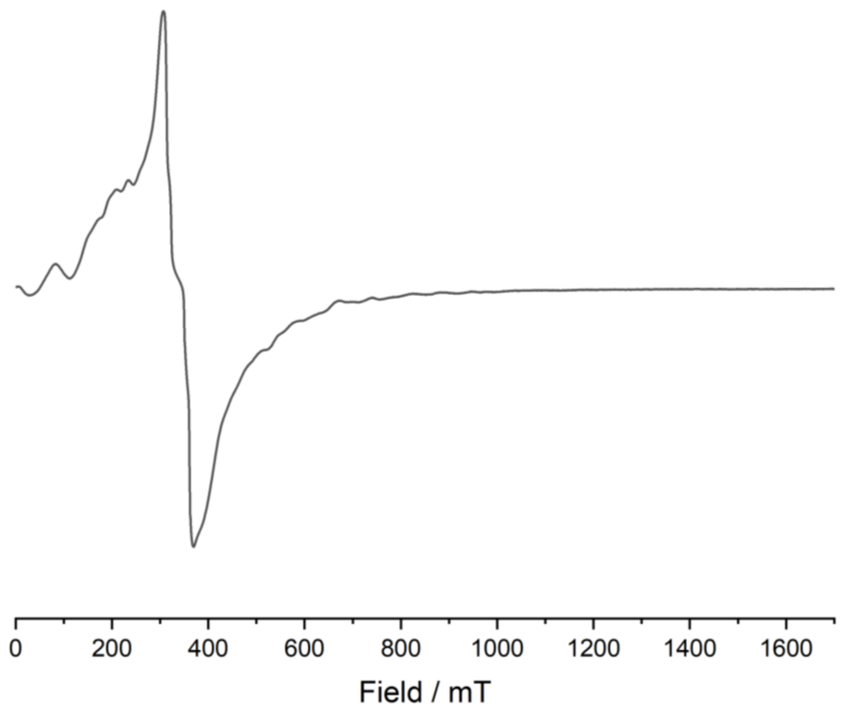

Figure S71. Powder X-band CW EPR spectrum of **1-Eu** measured at 5 K.

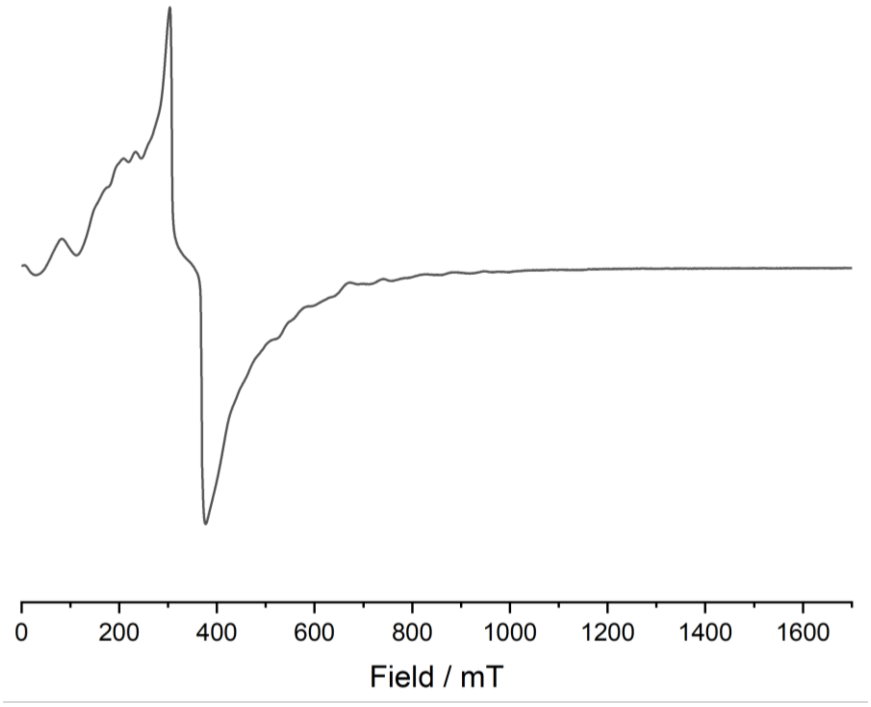

Figure S72. Powder X-band CW EPR spectrum of **1-Eu** measured at 10 K.

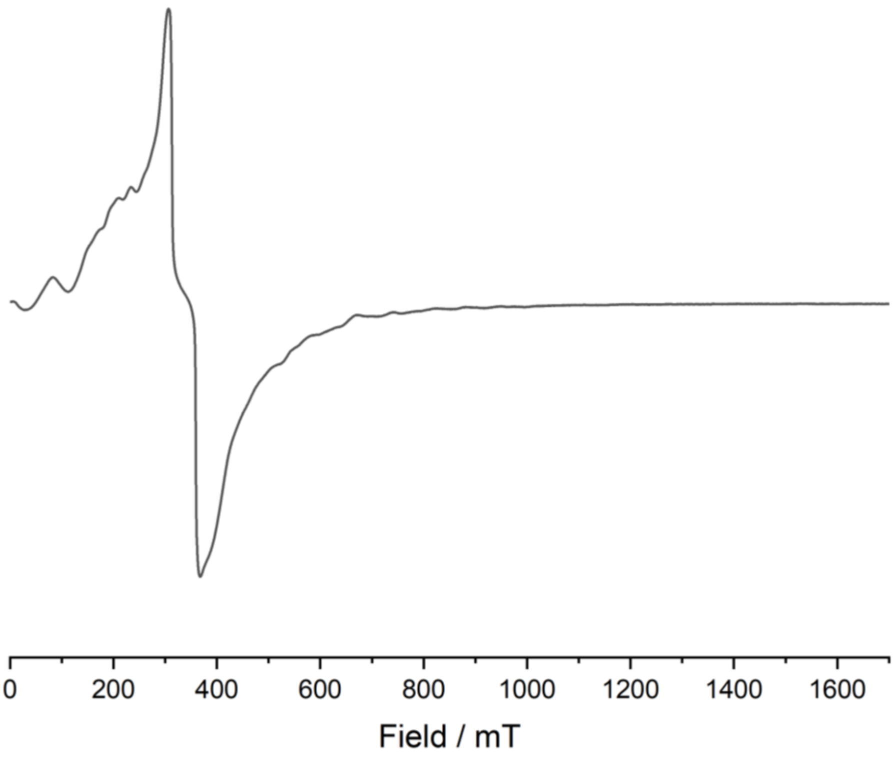

Figure S73. Powder X-band CW EPR spectrum of **1-Eu** measured at 20 K.

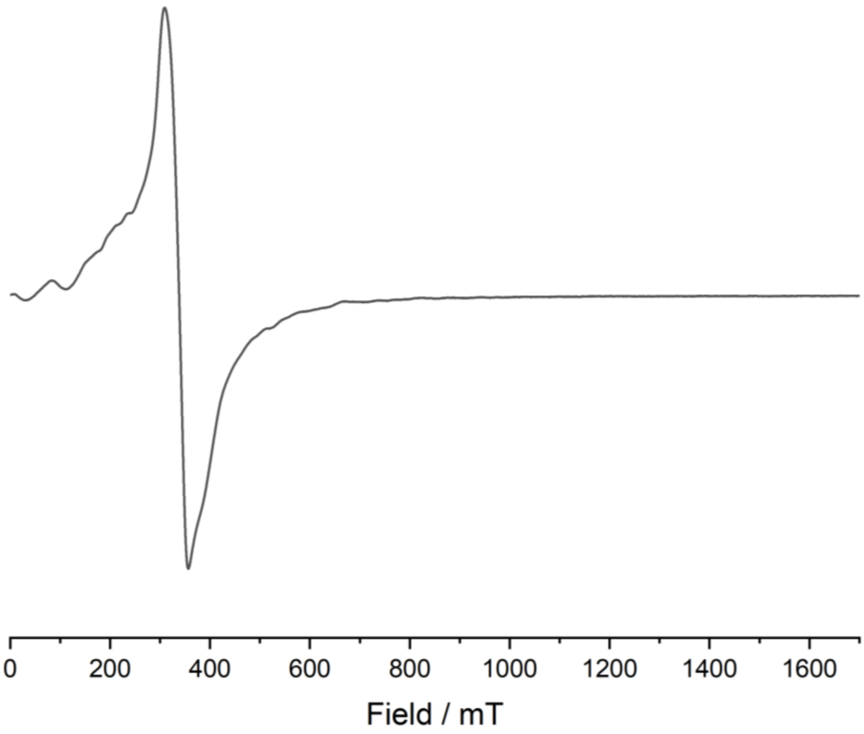

Figure S74. Powder X-band CW EPR spectrum of **1-Eu** measured at 50 K.

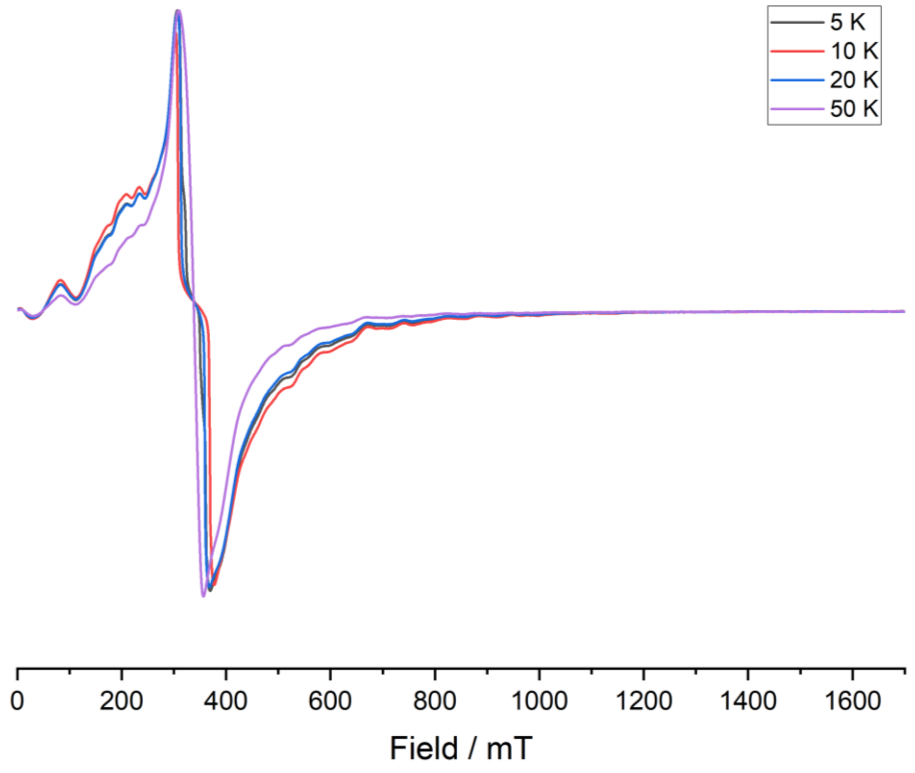

Figure S75. Overlaid powder X-band CW EPR spectra of **1-Eu** measured between 5-50 K.

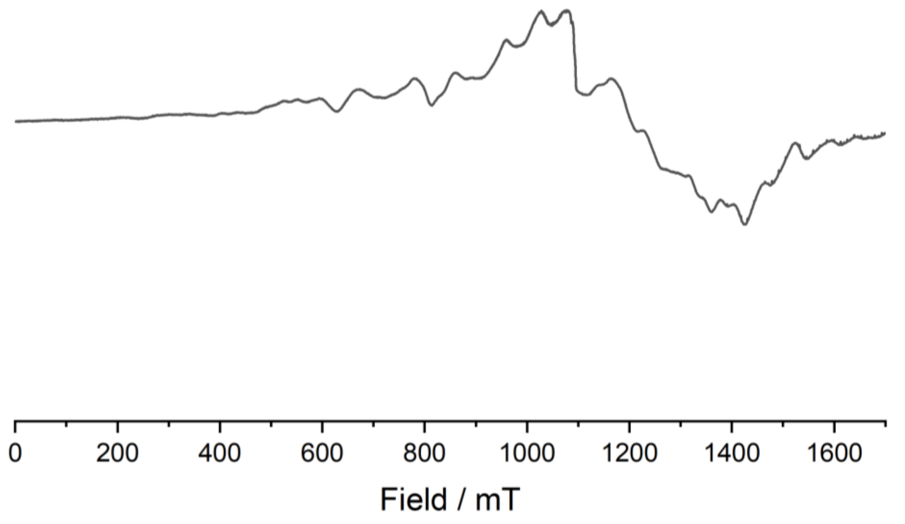

Figure S76. Powder Q-band CW EPR spectrum of **1-Eu** measured at 5 K.

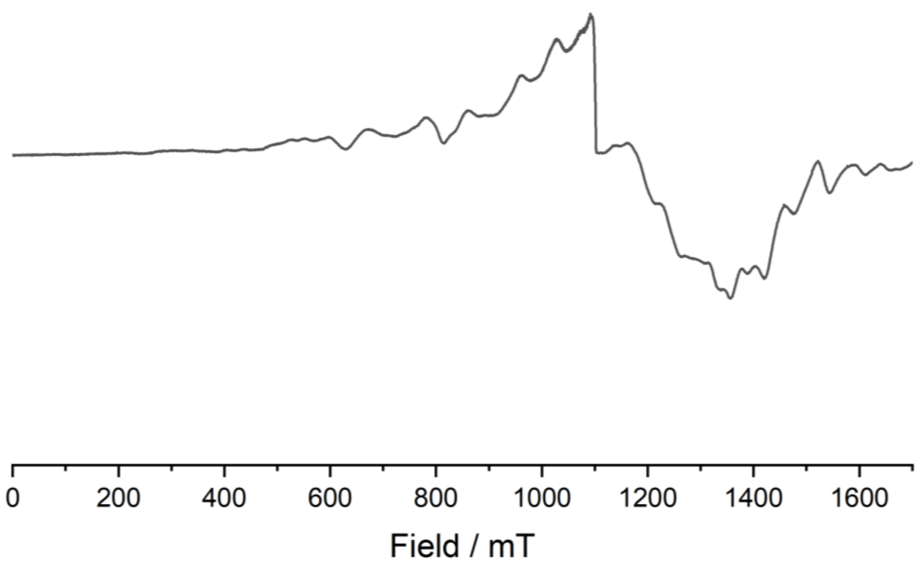

Figure S77. Powder Q-band CW EPR spectrum of **1-Eu** measured at 10 K.

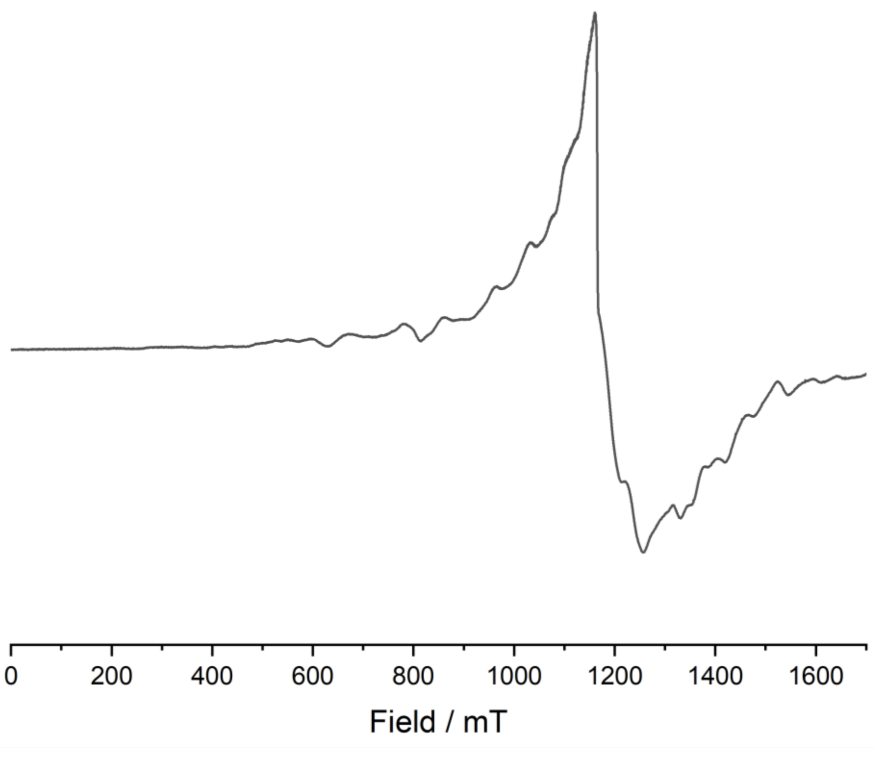

Figure S78. Powder Q-band CW EPR spectrum of **1-Eu** measured at 20 K.

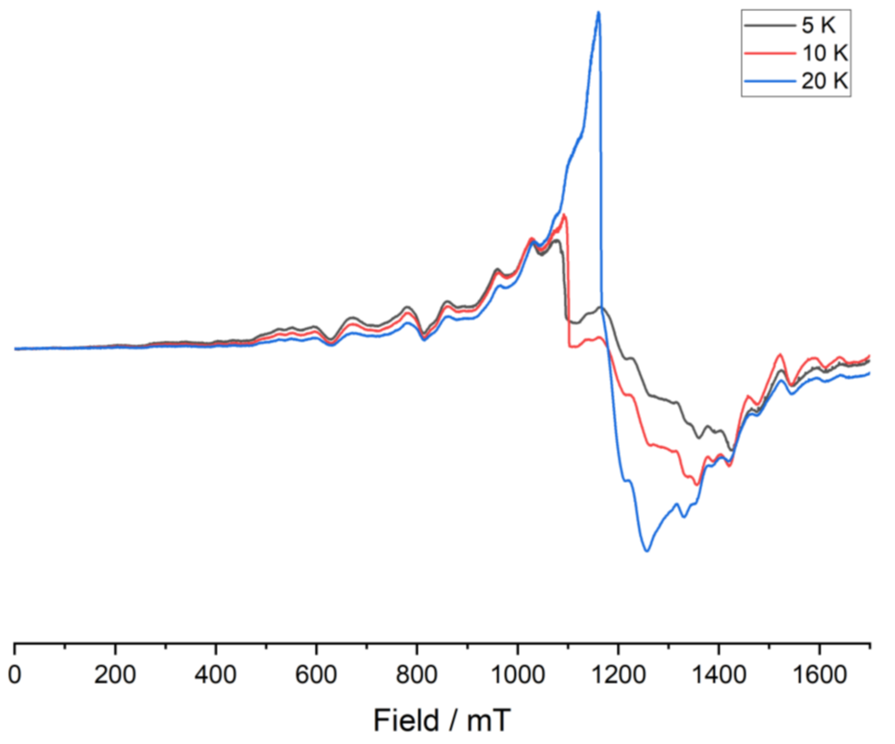

Figure S79. Overlaid powder Q-band CW EPR spectra of **1-Eu** measured between 5-20 K.

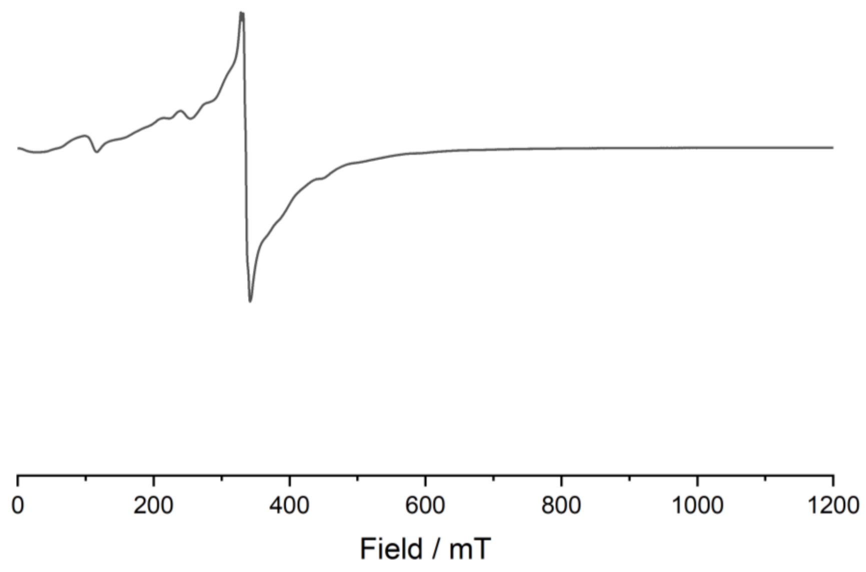

Figure S80. Frozen solution (10 mM) X-band CW EPR spectrum of **1-Eu** (9:1, toluene:hexane) measured at 5 K.

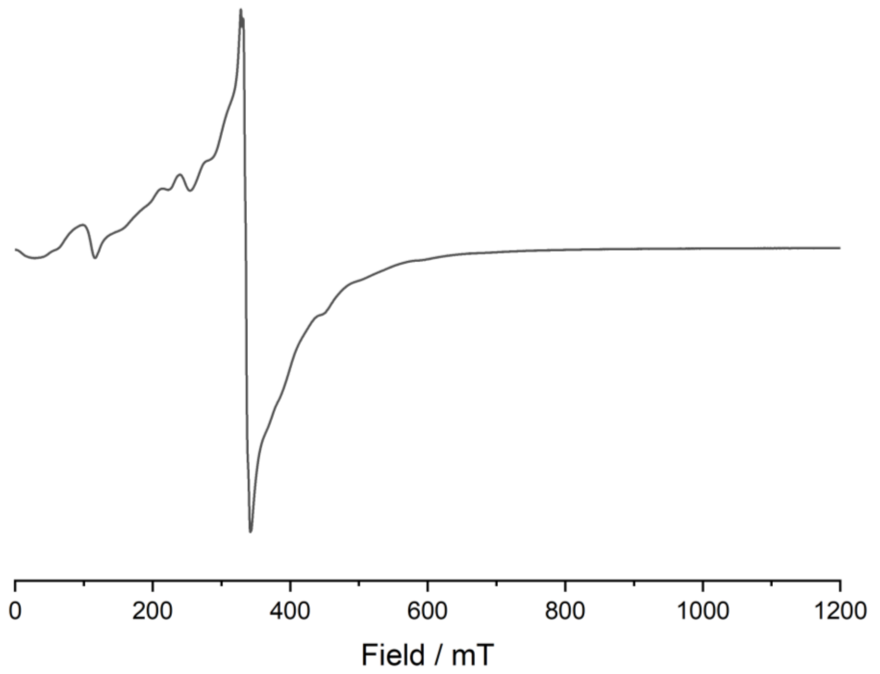

Figure S81. Frozen solution X-band (10 mM) CW EPR spectrum of **1-Eu** (9:1, toluene:hexane) measured at 10 K.

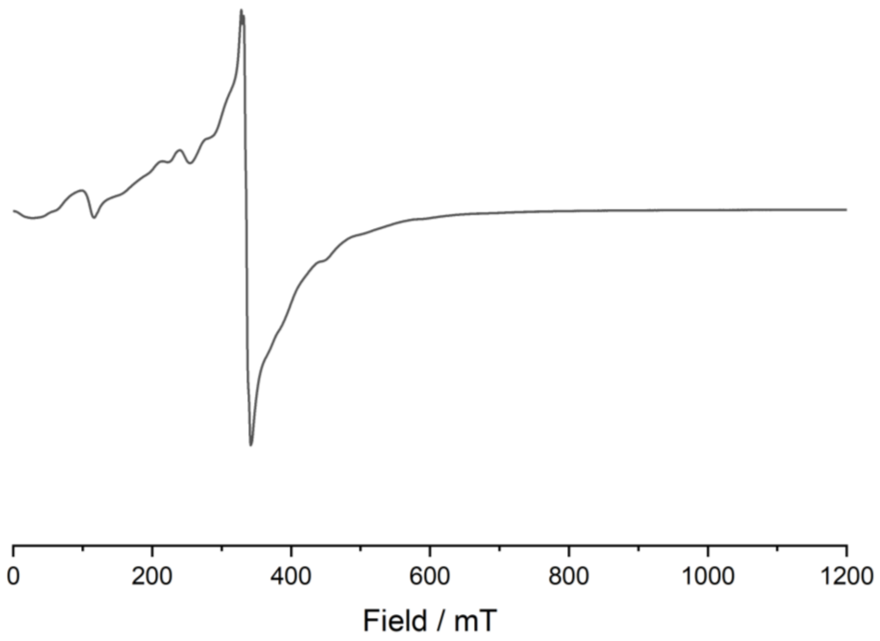

Figure S82. Frozen solution X-band (10 mM) CW EPR spectrum of **1-Eu** (9:1, toluene:hexane) measured at 20 K.

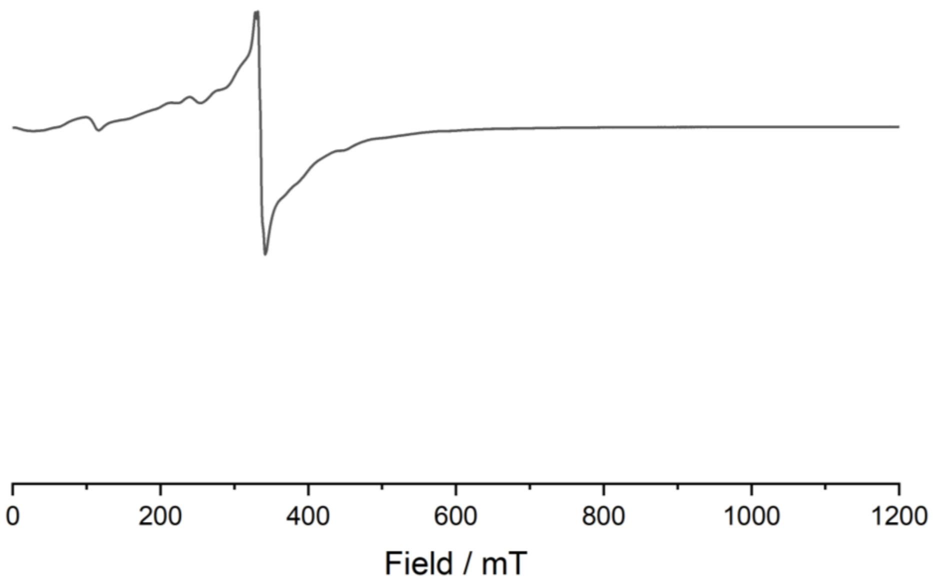

Figure S83. Frozen solution (10 mM) X-band CW EPR spectrum of **1-Eu** (9:1, toluene:hexane) measured at 50 K.

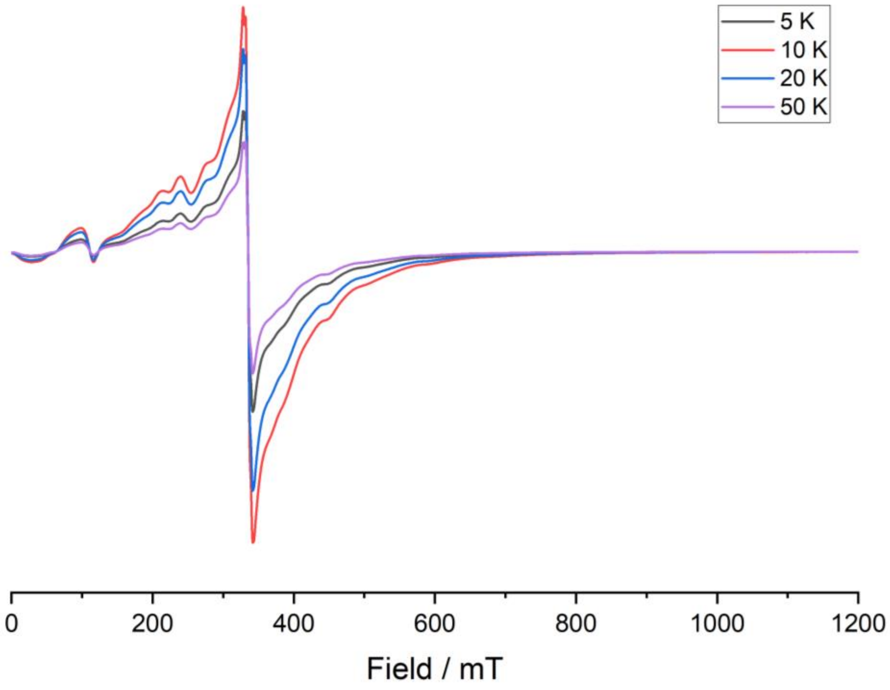

Figure S84. Overlaid frozen solution (10 mM) X-band CW EPR spectra of **1-Eu** measured between 5-50 K.

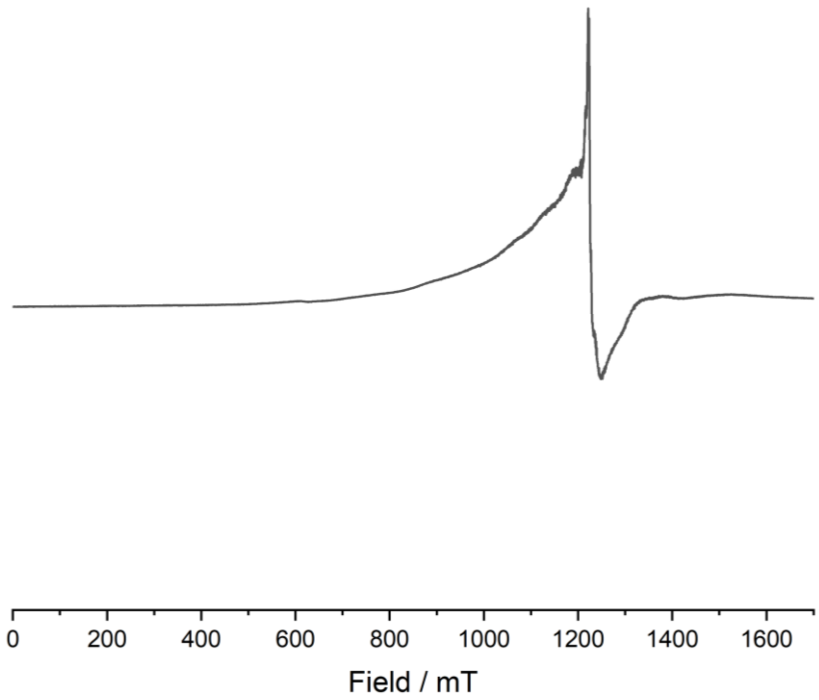

Figure S85. Frozen solution (10 mM) Q-band CW EPR spectrum of **1-Eu** (9:1, toluene:hexane) measured at 5 K.

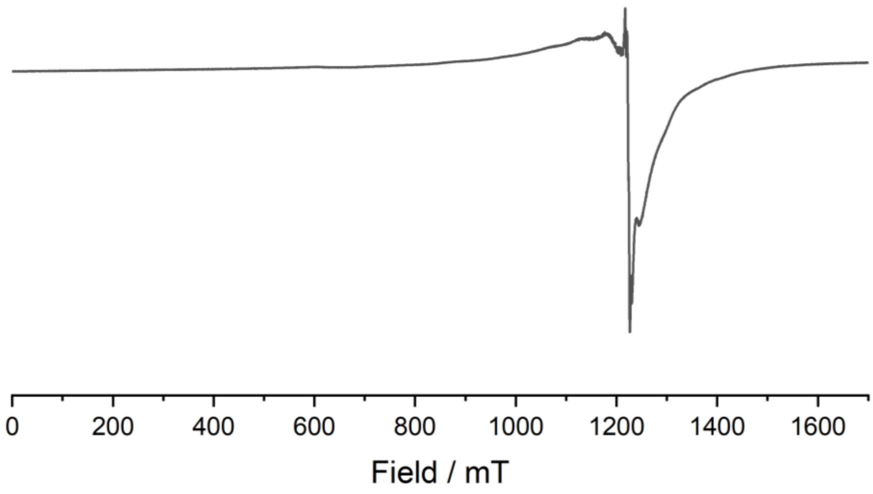

Figure S86. Frozen solution (10 mM) Q-band CW EPR spectrum of **1-Eu** (9:1, toluene:hexane) measured at 10 K.

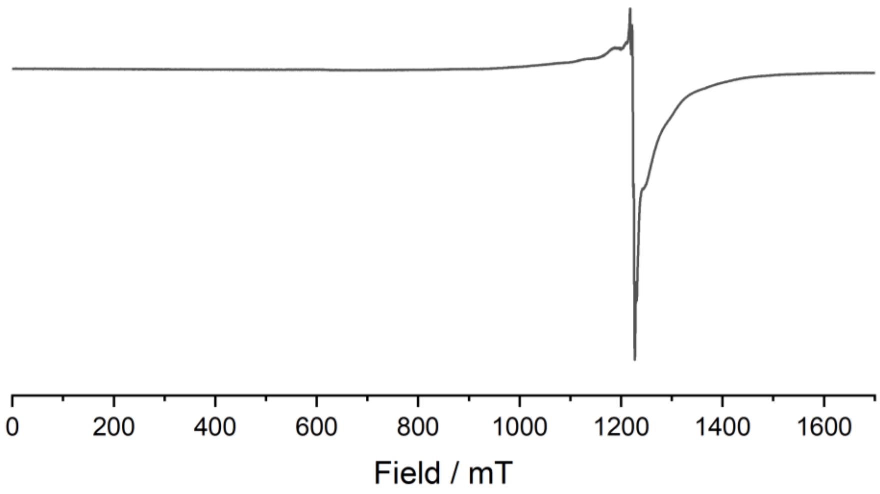

Figure S87. Frozen solution (10 mM) Q-band CW EPR spectrum of **1-Eu** (9:1, toluene:hexane) measured at 20 K.

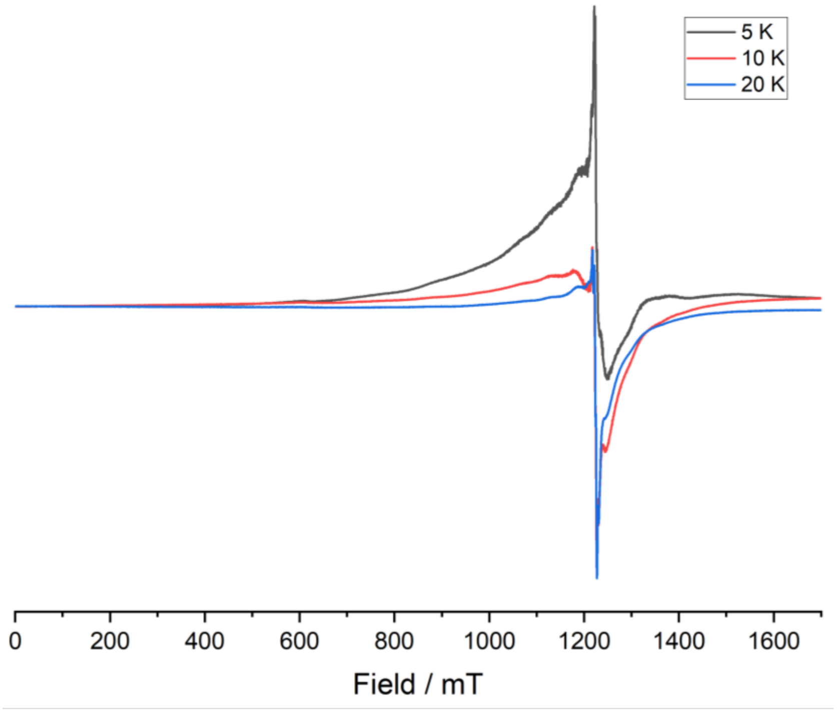

Figure S88. Overlaid frozen solution (10 mM) Q-band CW EPR spectra of **1-Eu** measured between 5-20 K.

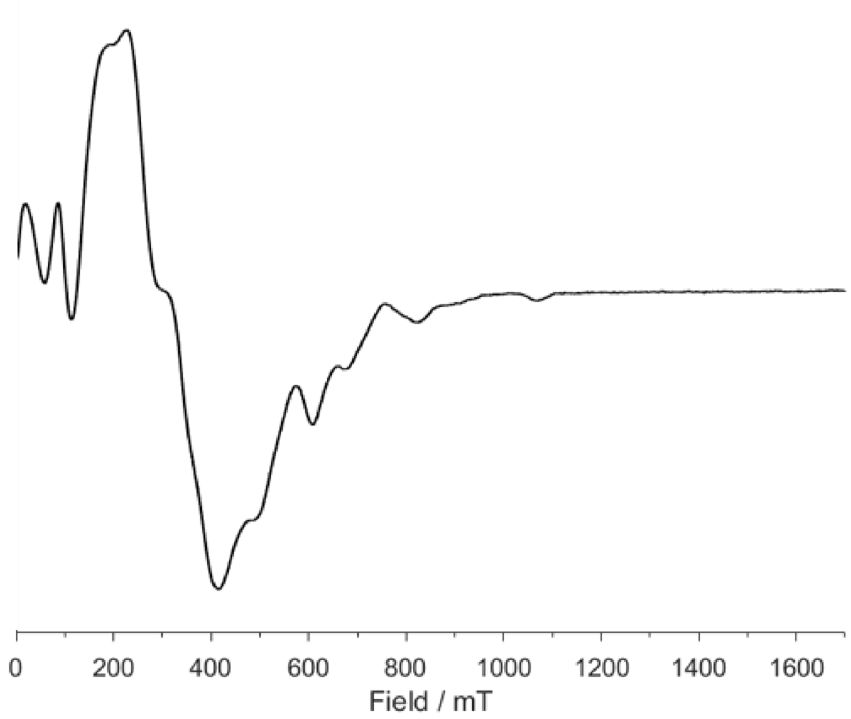

Figure S89. Powder X-band CW EPR spectrum of **3-Eu** measured at 5 K.

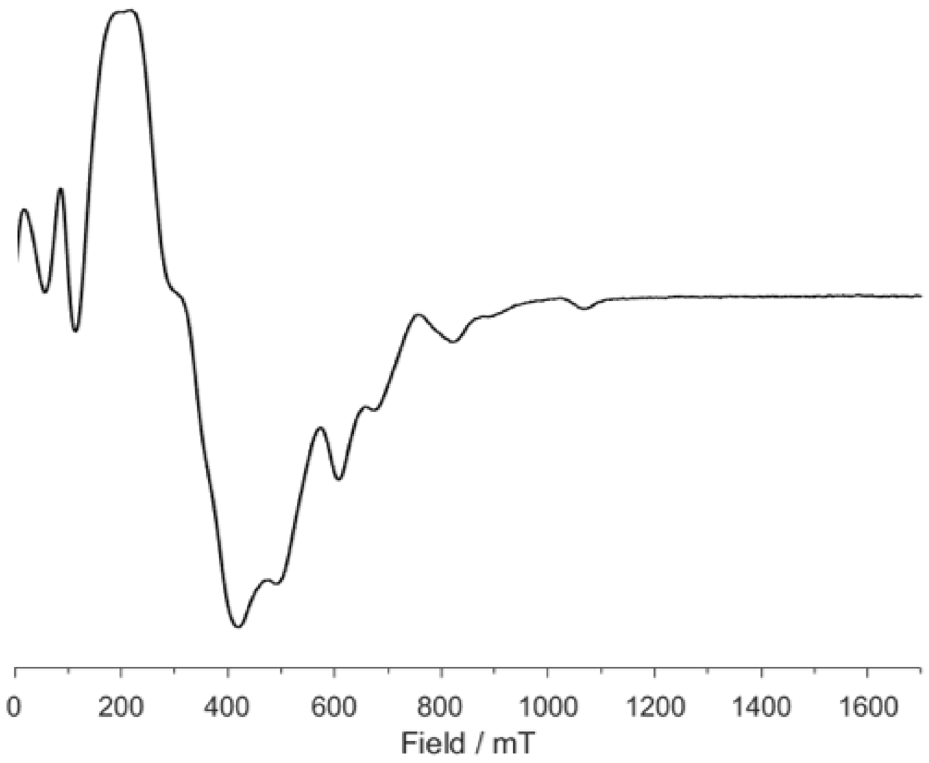

Figure S90. Powder X-band CW EPR spectrum of **3-Eu** measured at 10 K.

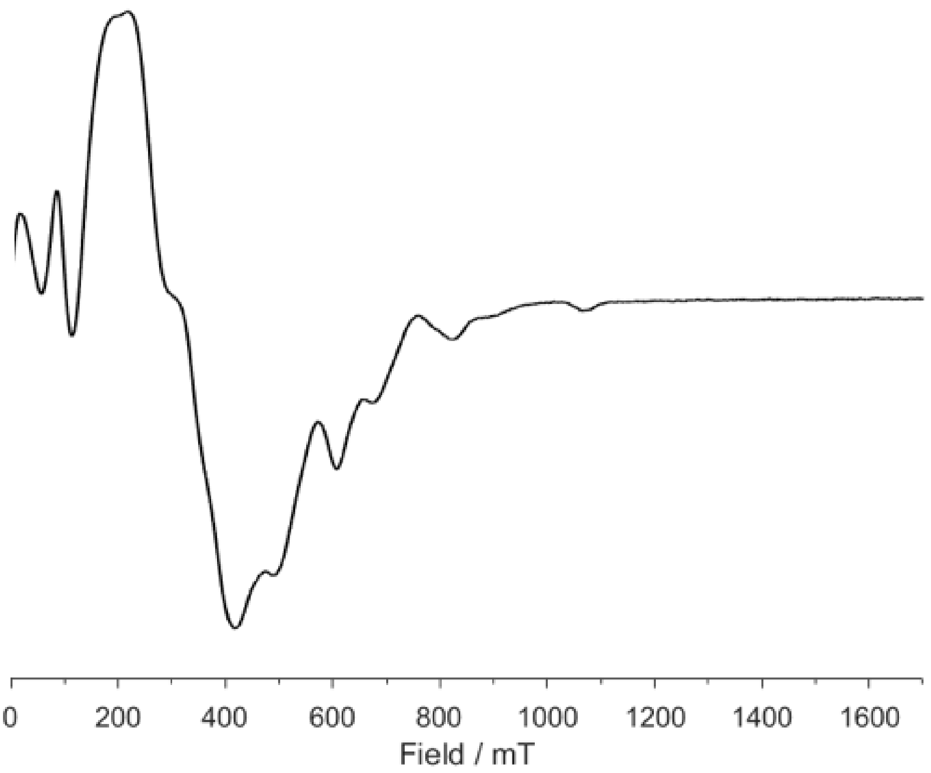

Figure S91. Powder X-band CW EPR spectrum of **3-Eu** measured at 20 K.

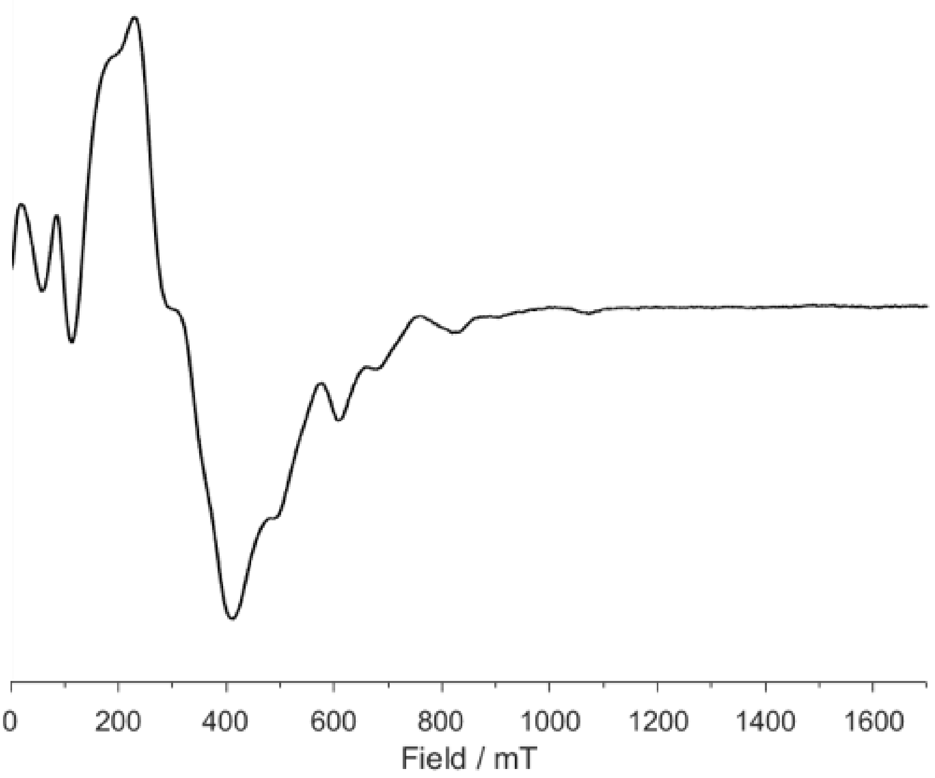

Figure S92. Powder X-band CW EPR spectrum of **3-Eu** measured at 50 K.

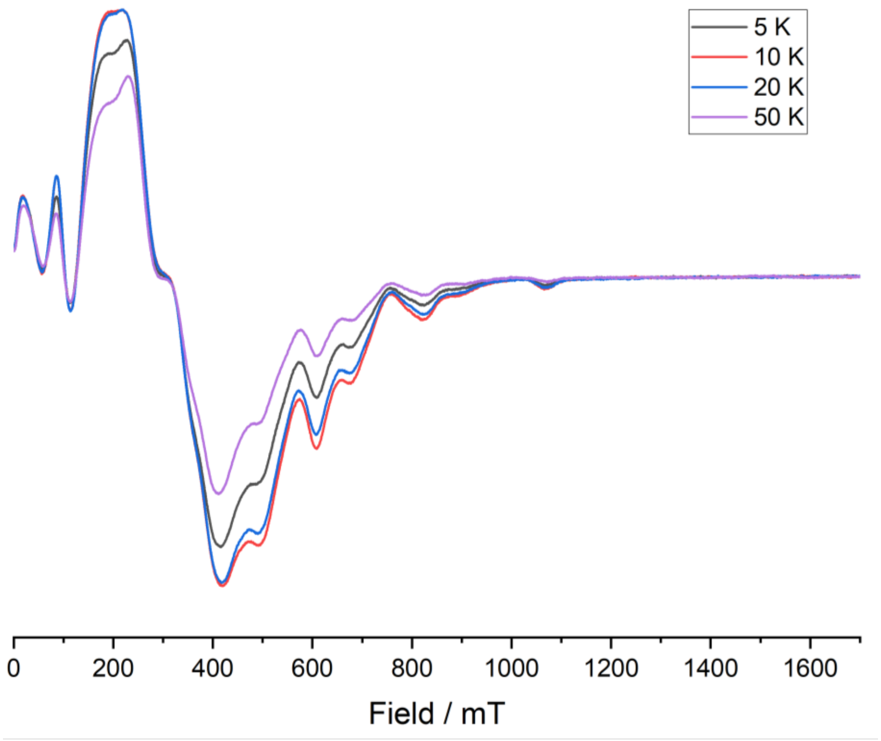

Figure S93. Overlaid powder X-band CW EPR spectra of **3-Eu** measured between 5-50 K.

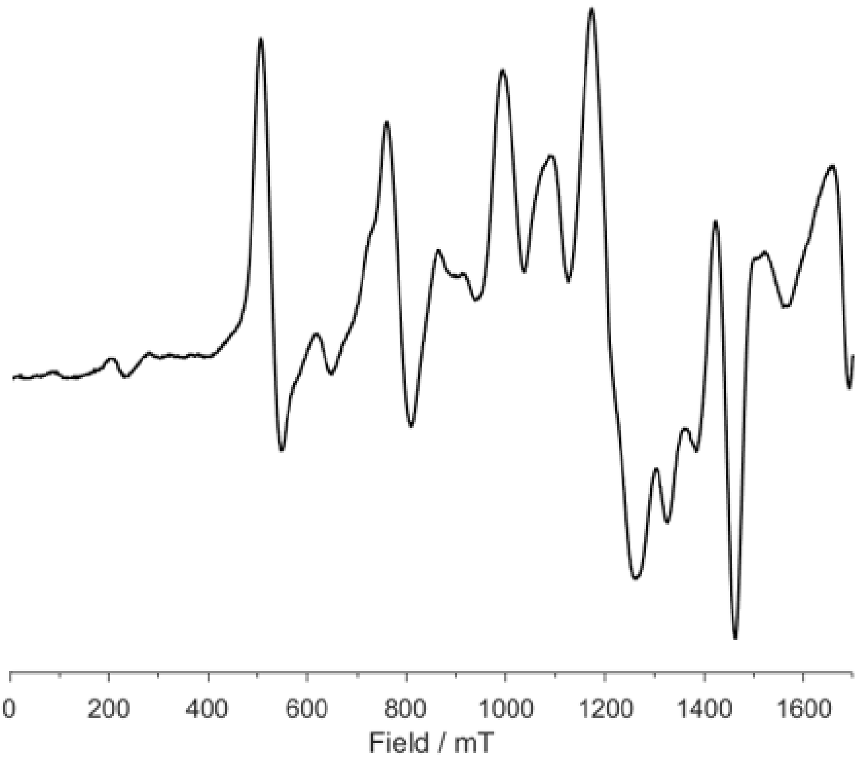

Figure S94. Powder Q-band CW EPR spectrum of **3-Eu** measured at 5 K.

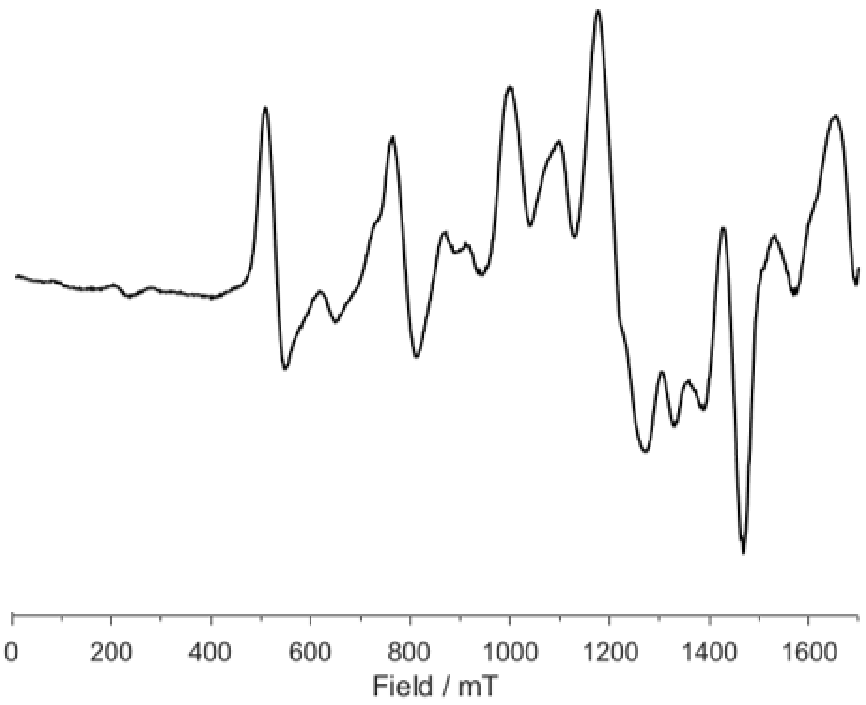

Figure S95. Powder Q-band CW EPR spectrum of **3-Eu** measured at 10 K.

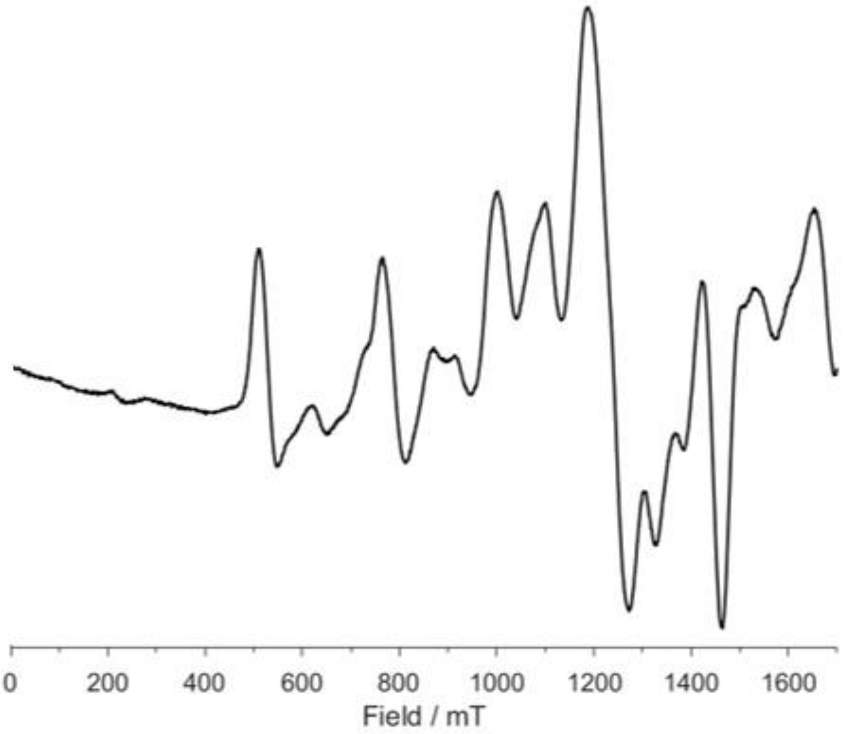

Figure S96. Powder Q-band CW EPR spectrum of **3-Eu** measured at 20 K.

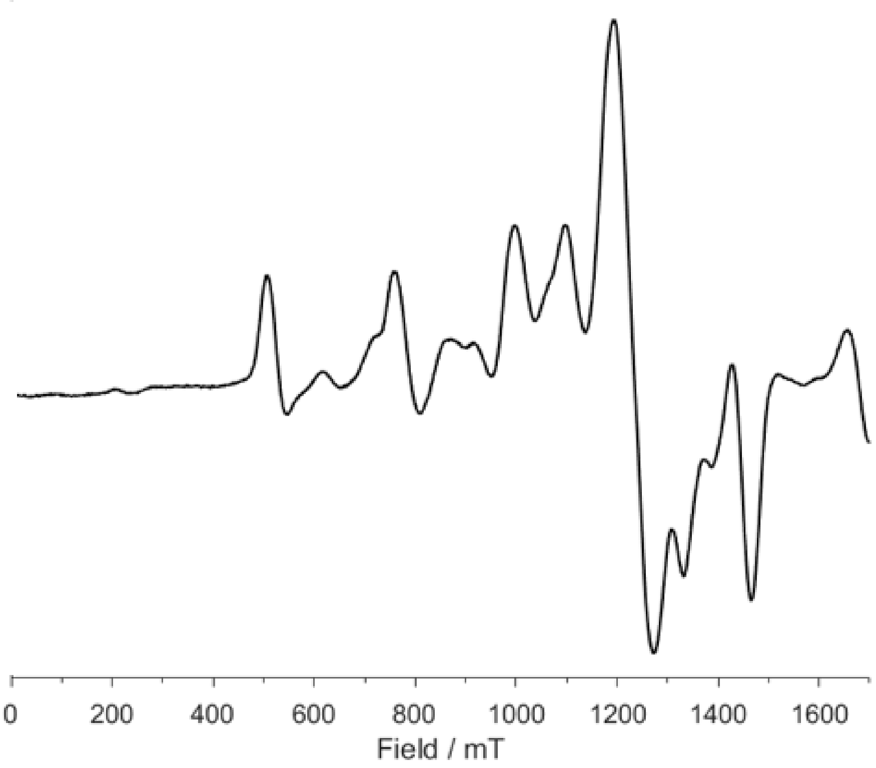

Figure S97. Powder Q-band CW EPR spectrum of **3-Eu** measured at 50 K.

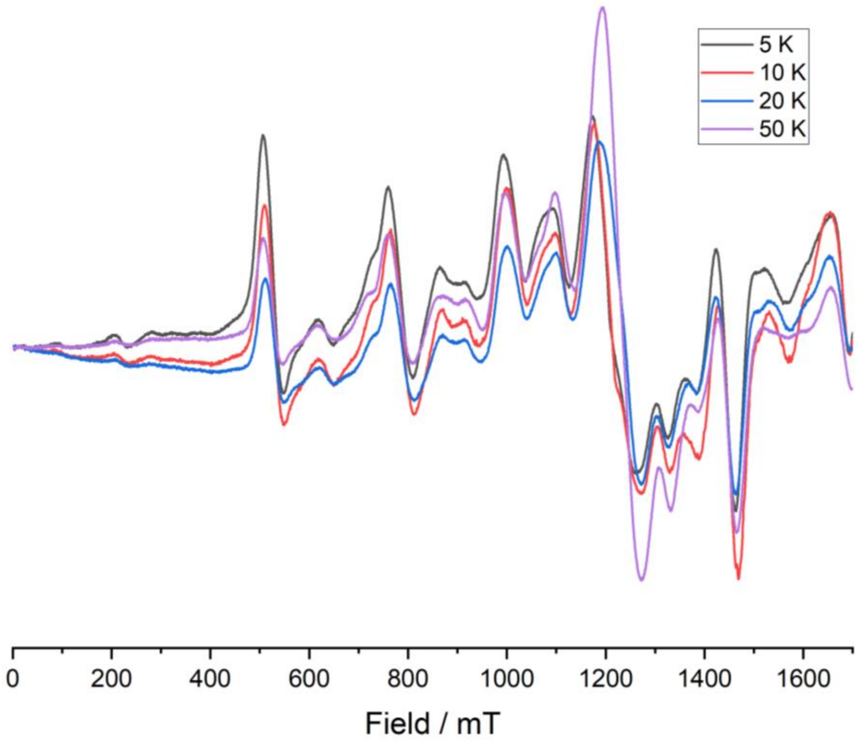

Figure S98. Overlaid powder Q-band CW EPR spectra of **3-Eu** measured between 5-50 K.

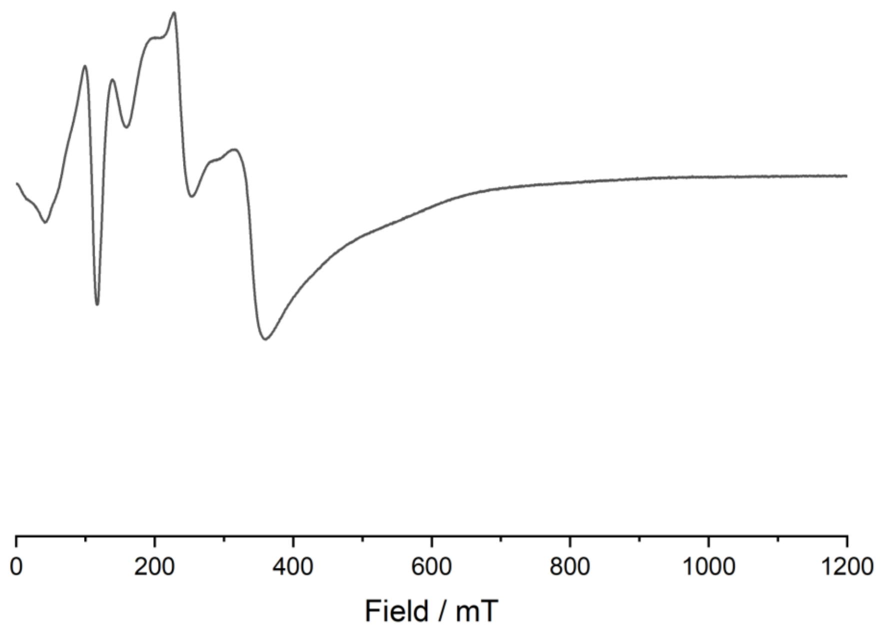

Figure S99. Frozen solution (10 mM) X-band CW EPR spectrum of **3-Eu** (9:1, toluene:hexane) measured at 5 K.

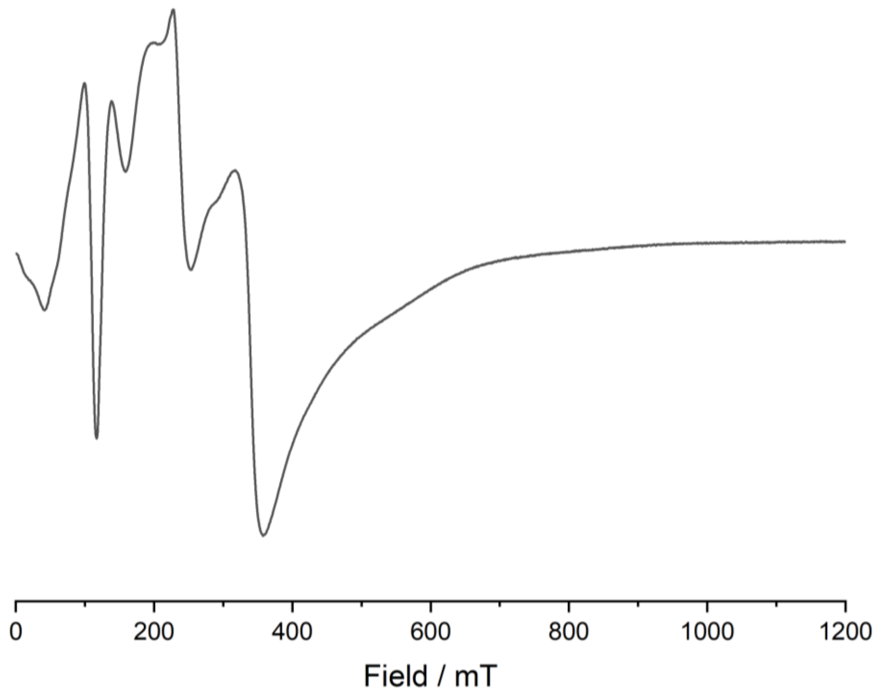

Figure S100. Frozen solution (10 mM) X-band CW EPR spectrum of **3-Eu** (9:1, toluene:hexane) measured at 10 K.

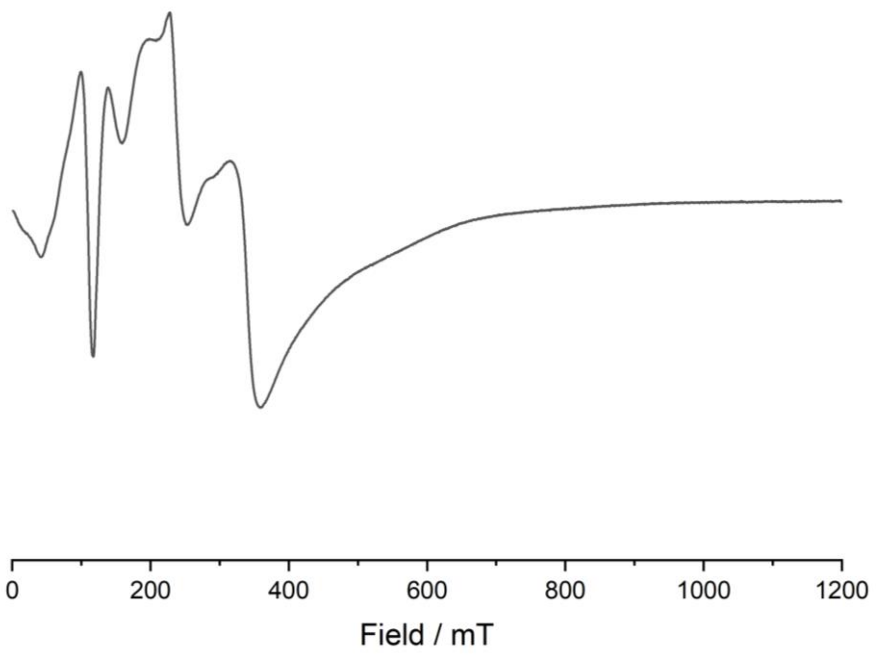

Figure S101. Frozen solution (10 mM) X-band CW EPR spectrum of **3-Eu** (9:1, toluene:hexane) measured at 20 K.

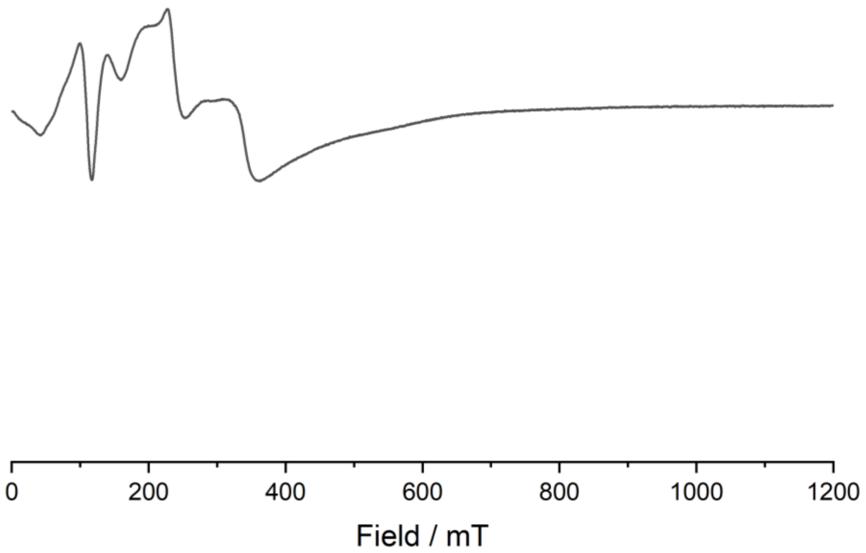

Figure S102. Frozen solution (10 mM) X-band CW EPR spectrum of **3-Eu** (9:1, toluene:hexane) measured at 50 K.

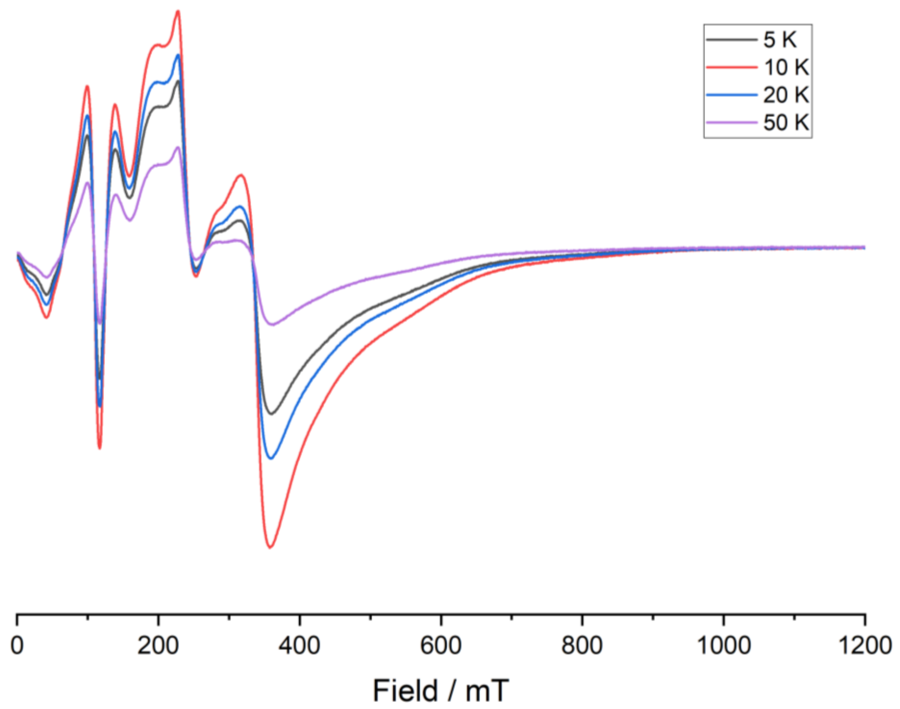

Figure S103. Overlaid frozen solution (10 mM) X-band CW EPR spectra of **3-Eu** measured between 5-50 K.

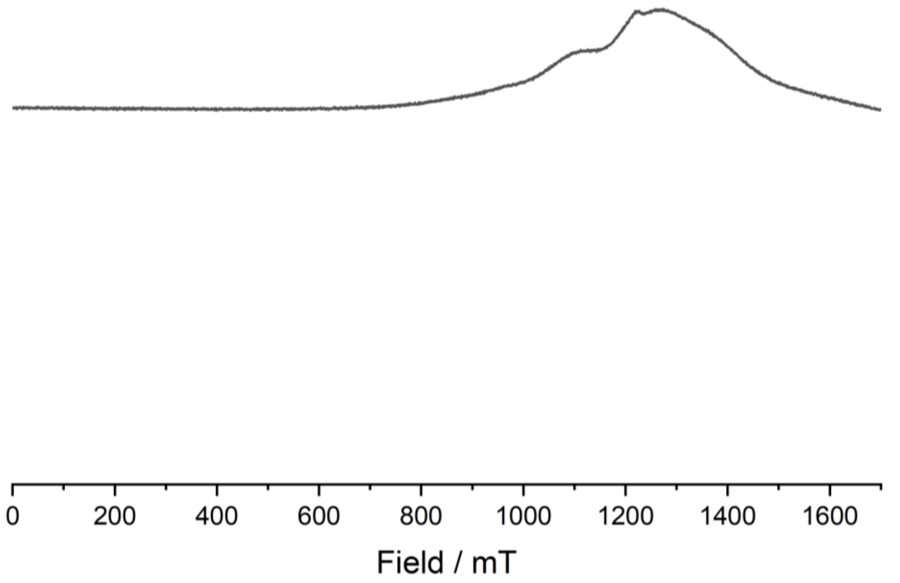

Figure S104. Frozen solution (10 mM) Q-band CW EPR spectrum of **3-Eu** (9:1, toluene:hexane) measured at 5 K.

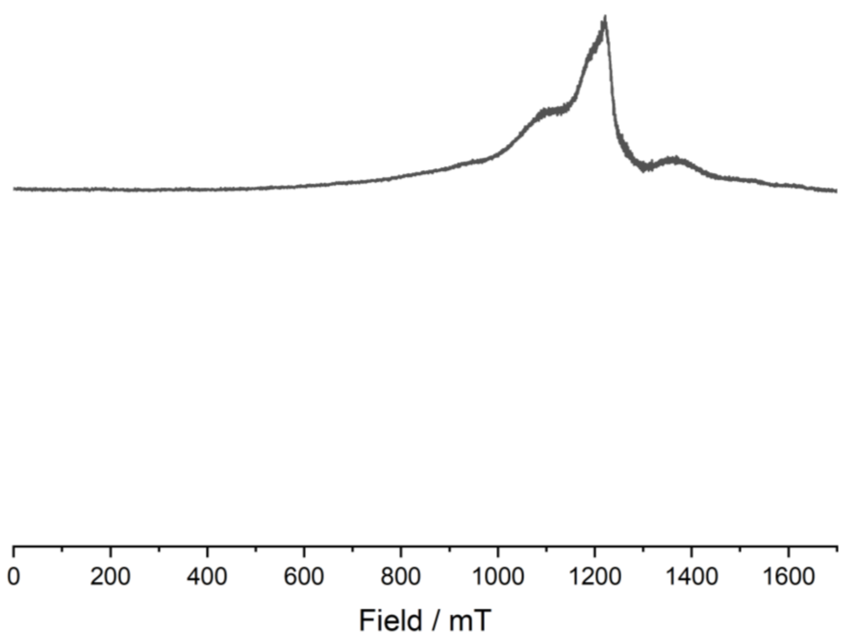

Figure S105. Frozen solution (10 mM) Q-band CW EPR spectrum of **3-Eu** (9:1, toluene:hexane) measured at 10 K.

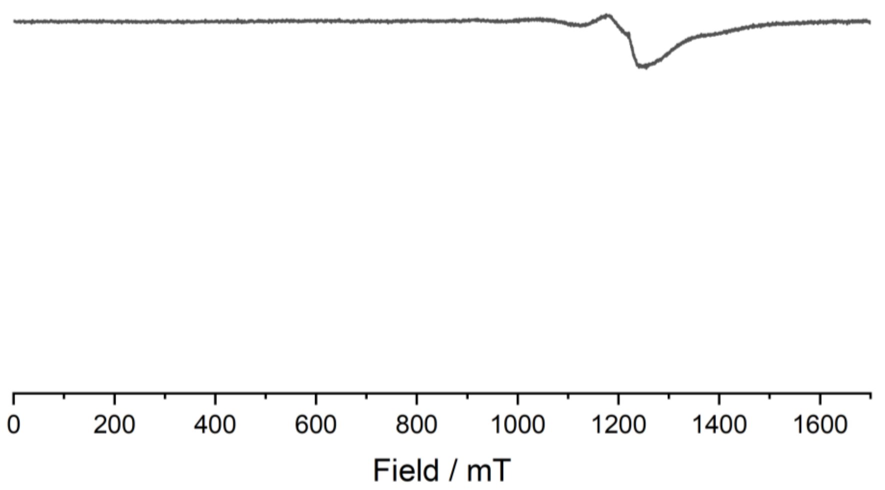

Figure S106. Frozen solution (10 mM) Q-band CW EPR spectrum of **3-Eu** (9:1, toluene:hexane) measured at 20 K.

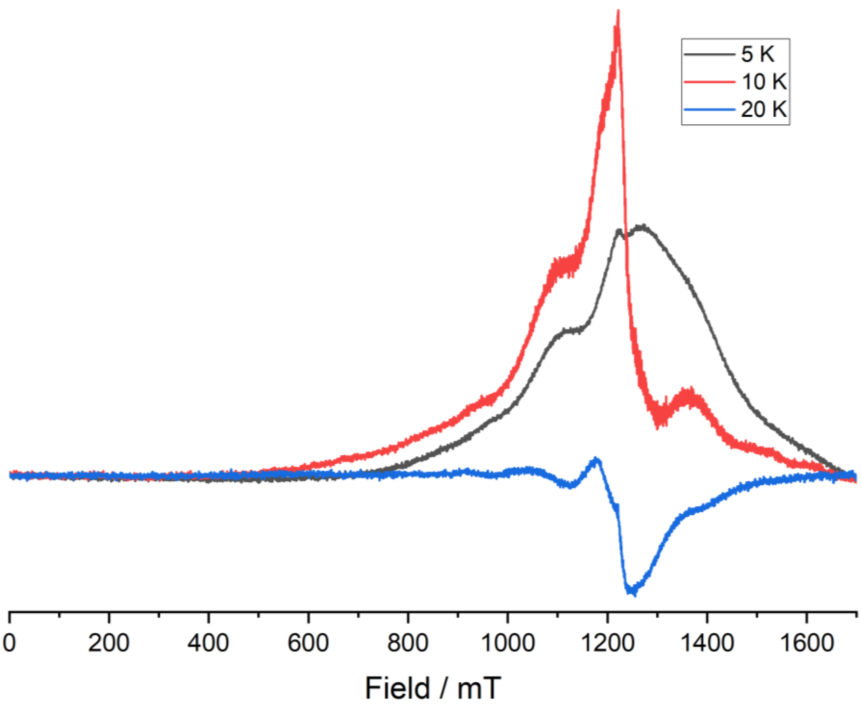

Figure S107. Overlaid frozen solution (10 mM) Q-band CW EPR spectra of **3-Eu** measured between 5-20 K.

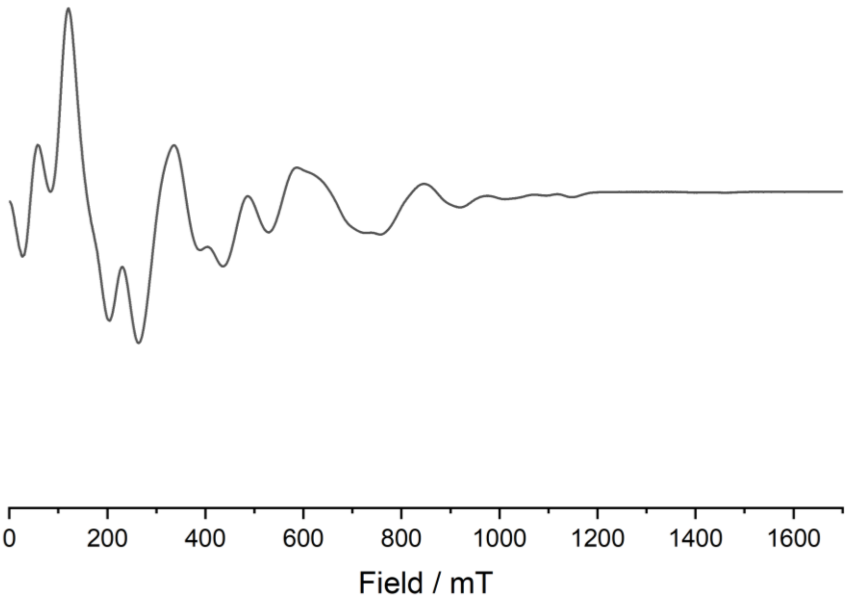

Figure S108. Powder X-band CW EPR spectrum of **4-Eu** measured at 5 K.

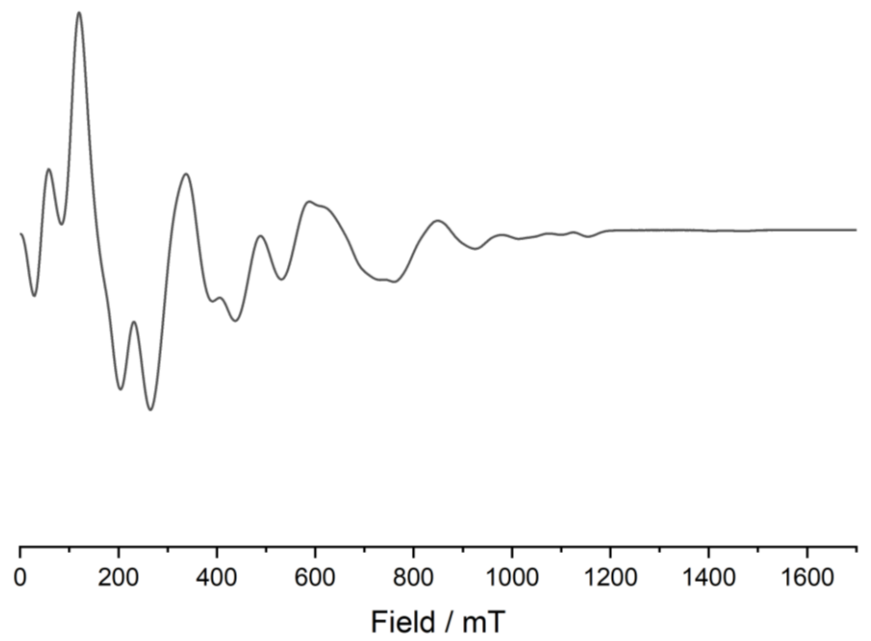

Figure S109. Powder X-band CW EPR spectrum of **4-Eu** measured at 10 K.

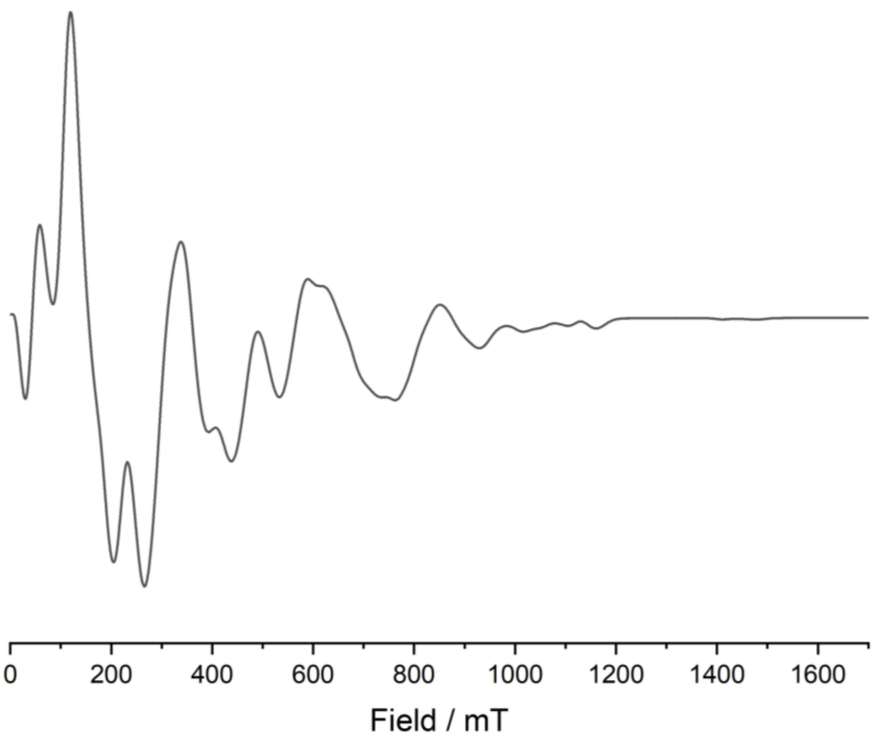

Figure S110. Powder X-band CW EPR spectrum of **4-Eu** measured at 20 K.

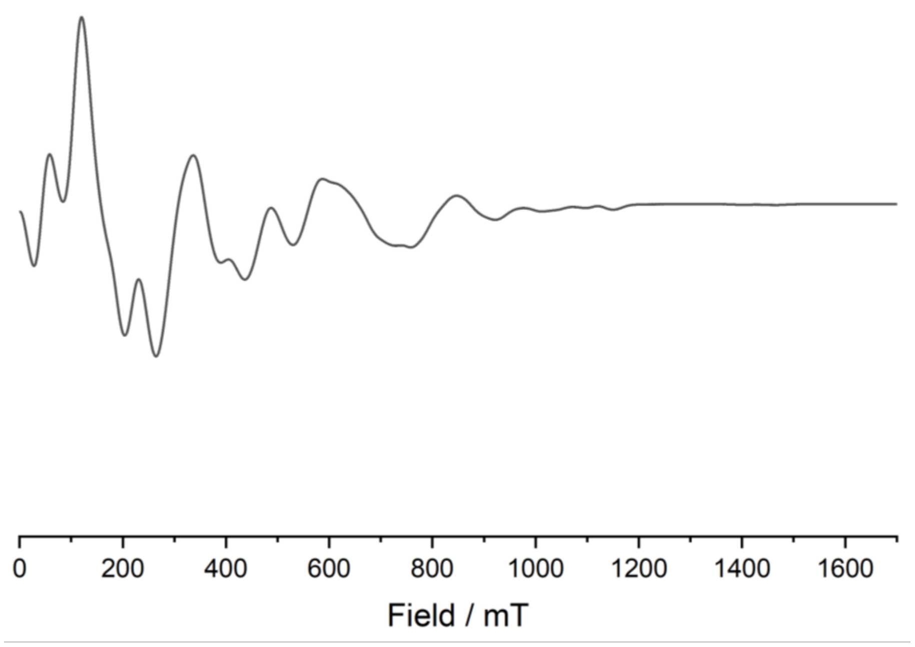

Figure S111. Powder X-band CW EPR spectrum of **4-Eu** measured at 50 K.

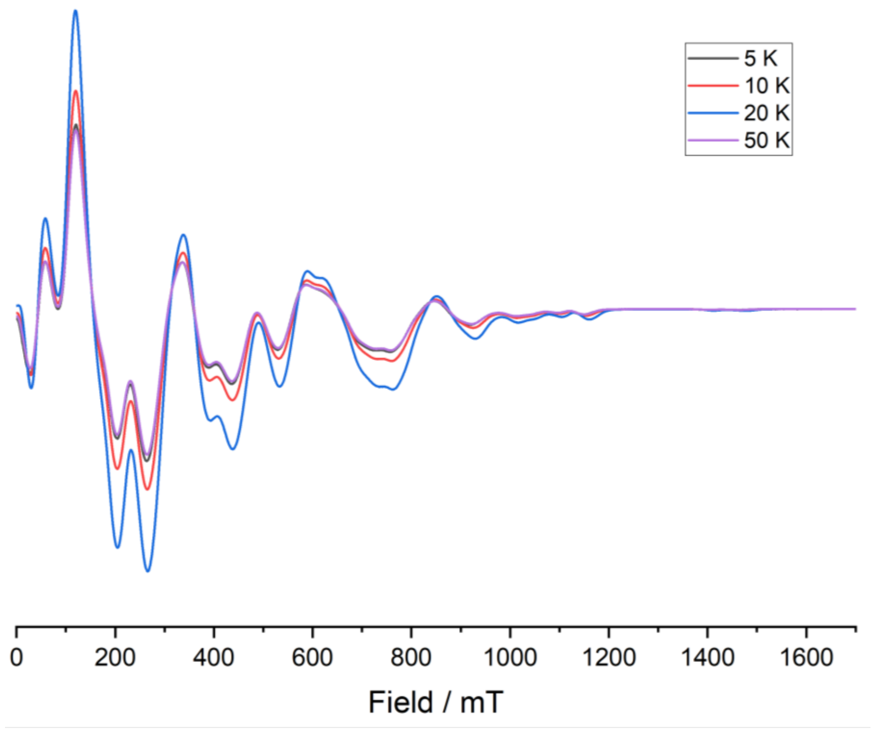

Figure S112. Overlaid powder X-band CW EPR spectra of **4-Eu** measured between 5-50 K.

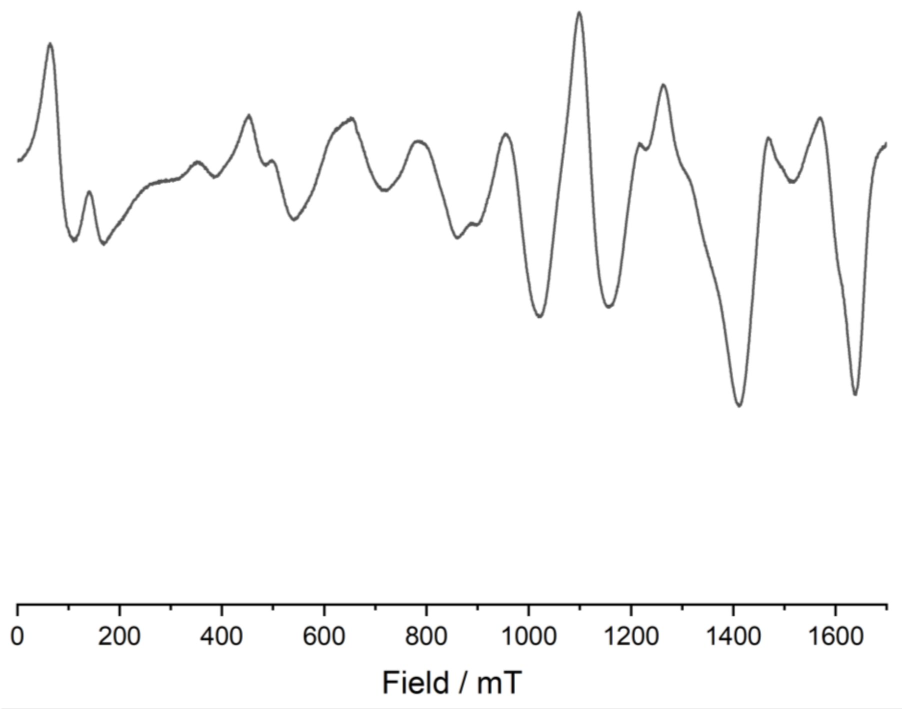

Figure S113. Powder Q-band CW EPR spectrum of **4-Eu** measured at 5 K.

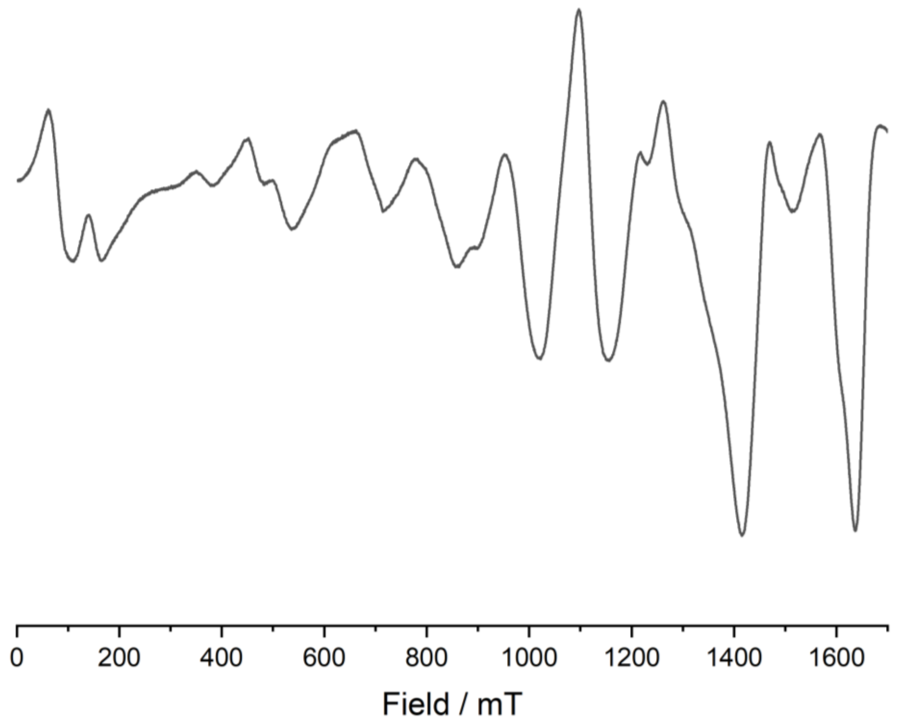

Figure S114. Powder Q-band CW EPR spectrum of **4-Eu** measured at 10 K.

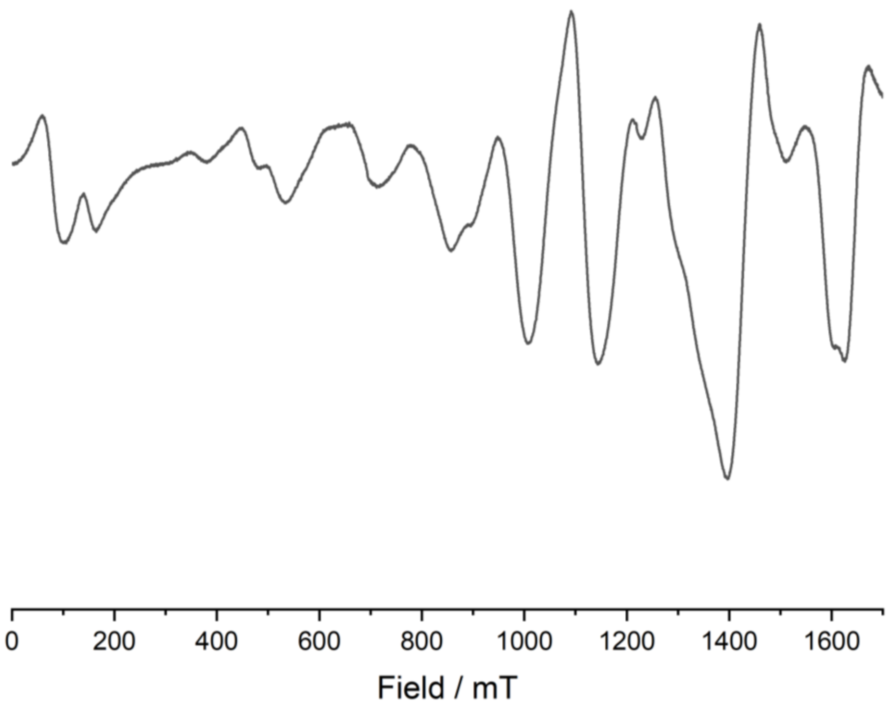

Figure S115. Powder Q-band CW EPR spectrum of **4-Eu** measured at 20 K.

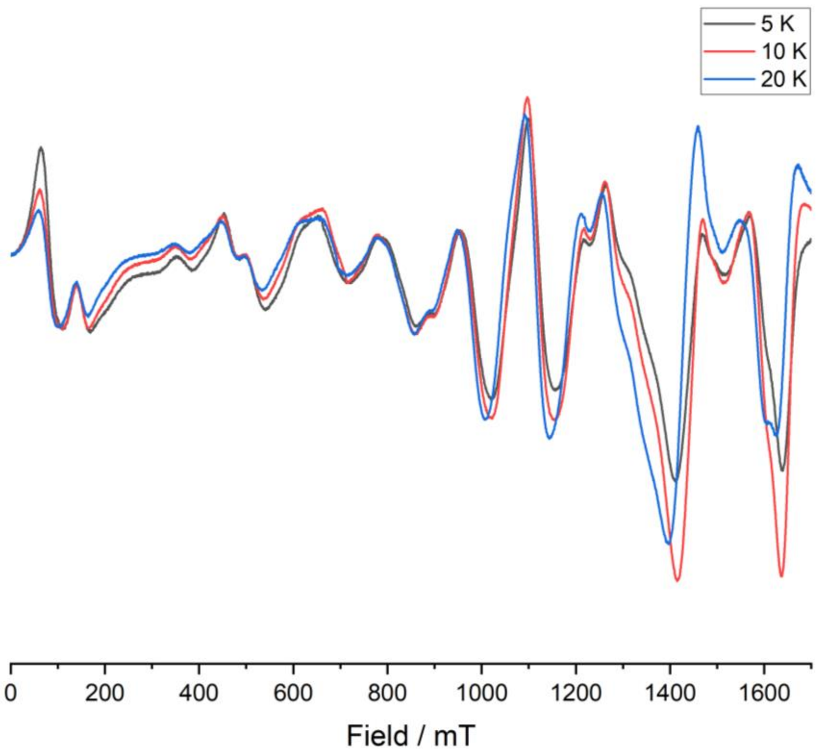

Figure S116. Overlaid powder Q-band CW EPR spectra of **4-Eu** measured between 5-20 K.

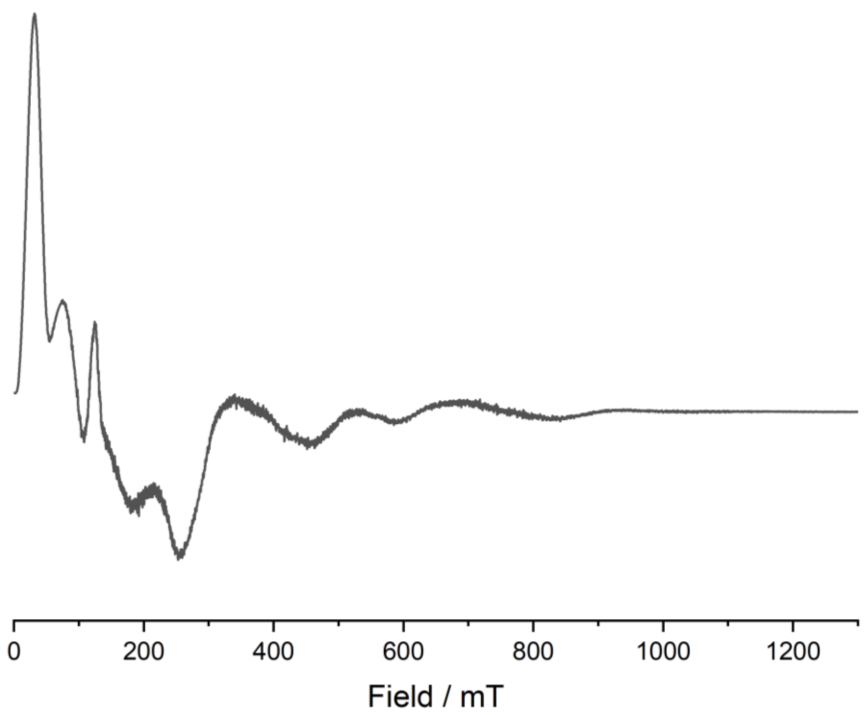

Figure S117. Frozen solution (10 mM) X-band CW EPR spectrum of **4-Eu** (9:1, toluene:hexane) measured at 5 K.

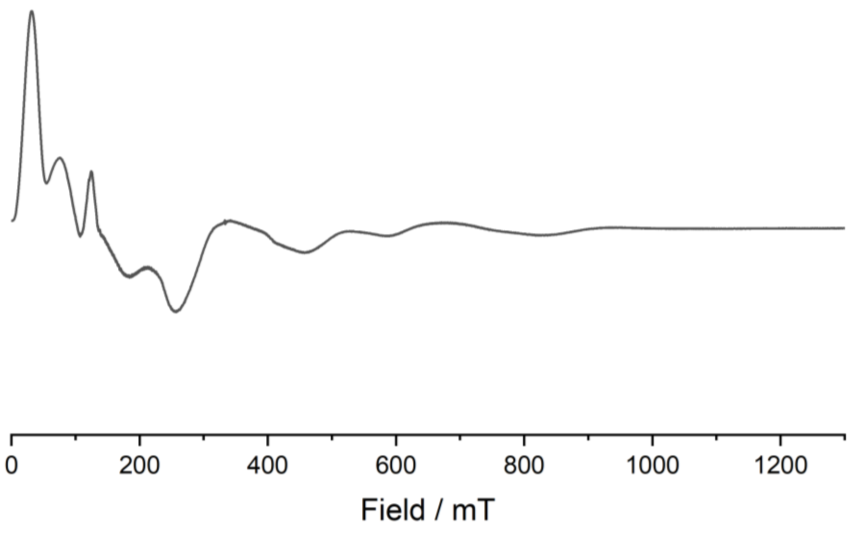

Figure S118. Frozen solution X-band (10 mM) CW EPR spectrum of **4-Eu** (9:1, toluene:hexane) measured at 10 K.

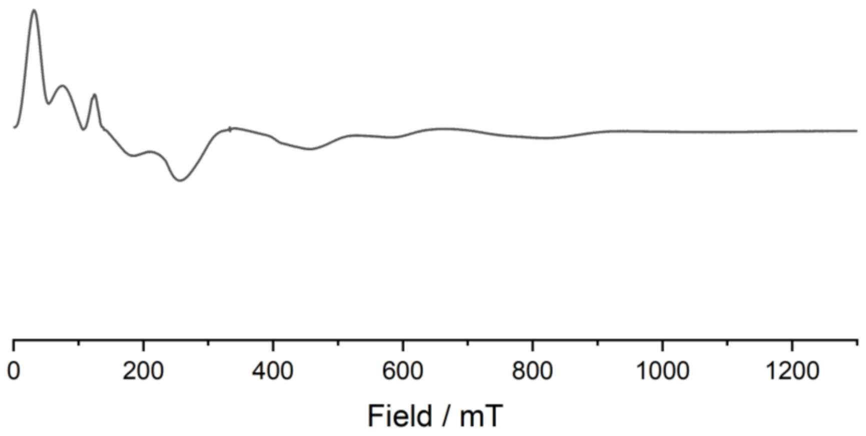

Figure S119. Frozen solution X-band (10 mM) CW EPR spectrum of **4-Eu** (9:1, toluene:hexane) measured at 20 K.

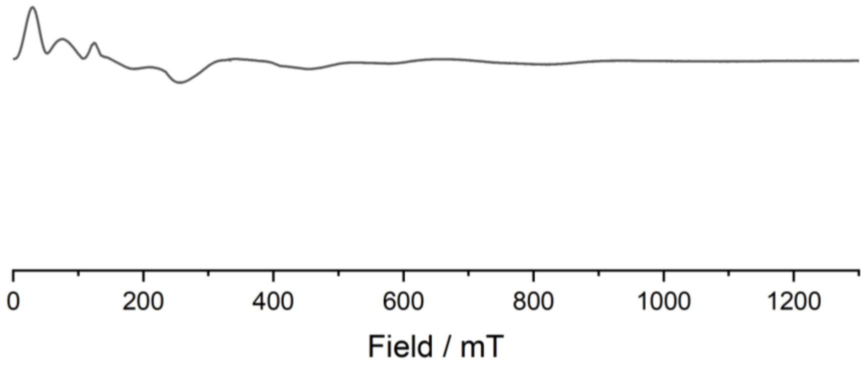

Figure S120. Frozen solution X-band (10 mM) CW EPR spectrum of **4-Eu** (9:1, toluene:hexane) measured at 50 K.

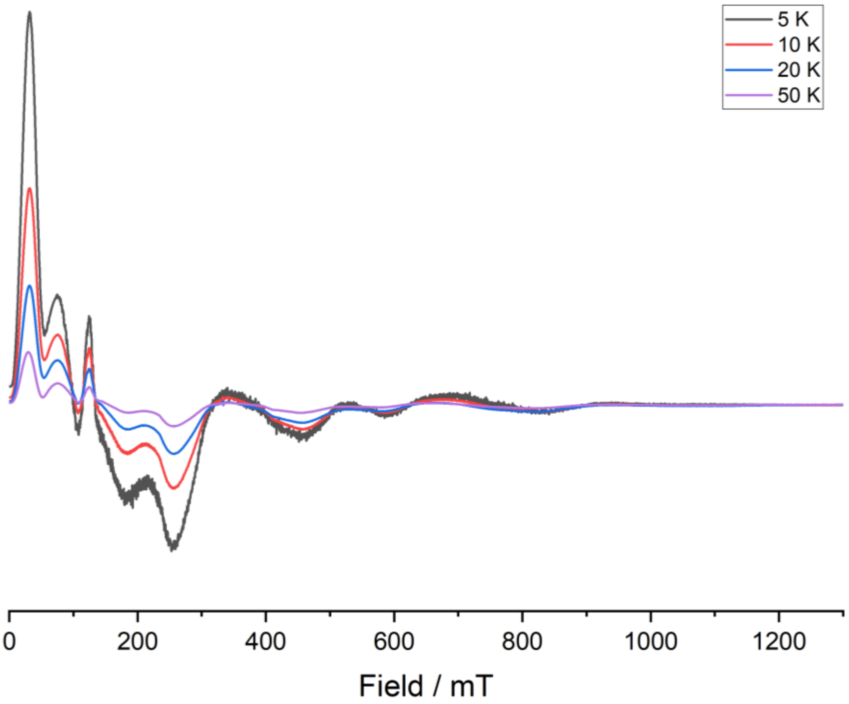

Figure S121. Overlaid frozen solution (10 mM) X-band CW EPR spectra of **4-Eu** measured between 5-50 K.

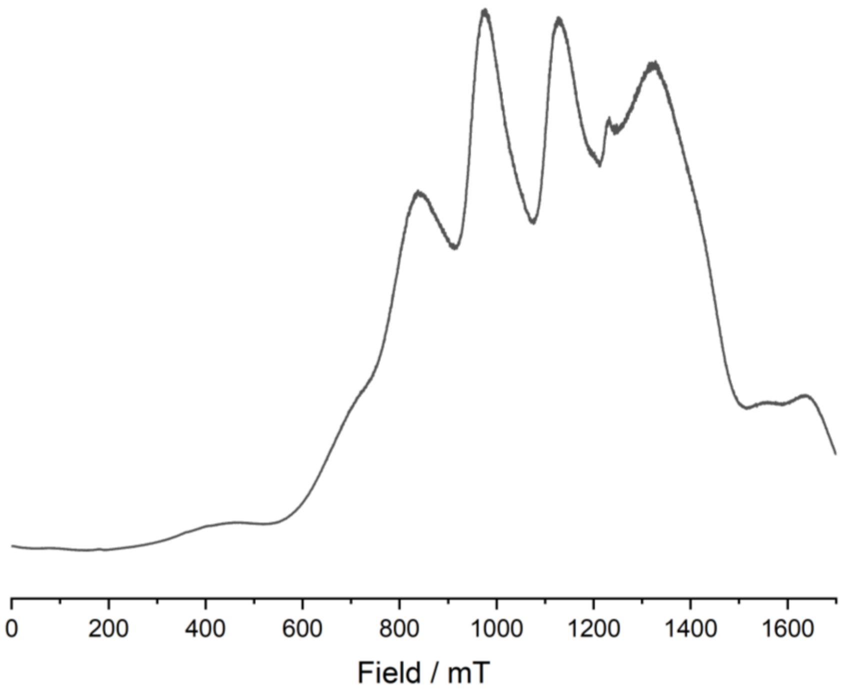

Figure S122. Frozen solution Q-band (10 mM) CW EPR spectrum of **4-Eu** (9:1, toluene:hexane) measured at 5 K.

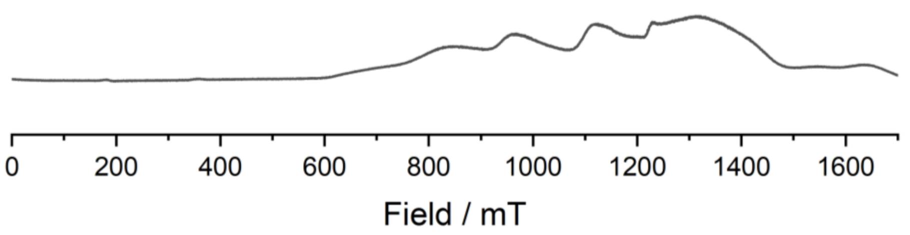

Figure S123. Frozen solution Q-band (10 mM) CW EPR spectrum of **4-Eu** (9:1, toluene:hexane) measured at 10 K.

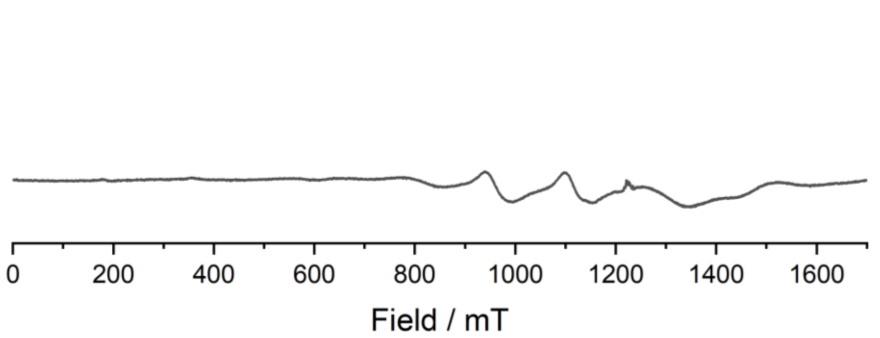

Figure S124. Frozen solution Q-band (10 mM) CW EPR spectrum of **4-Eu** (9:1, toluene:hexane) measured at 20 K.

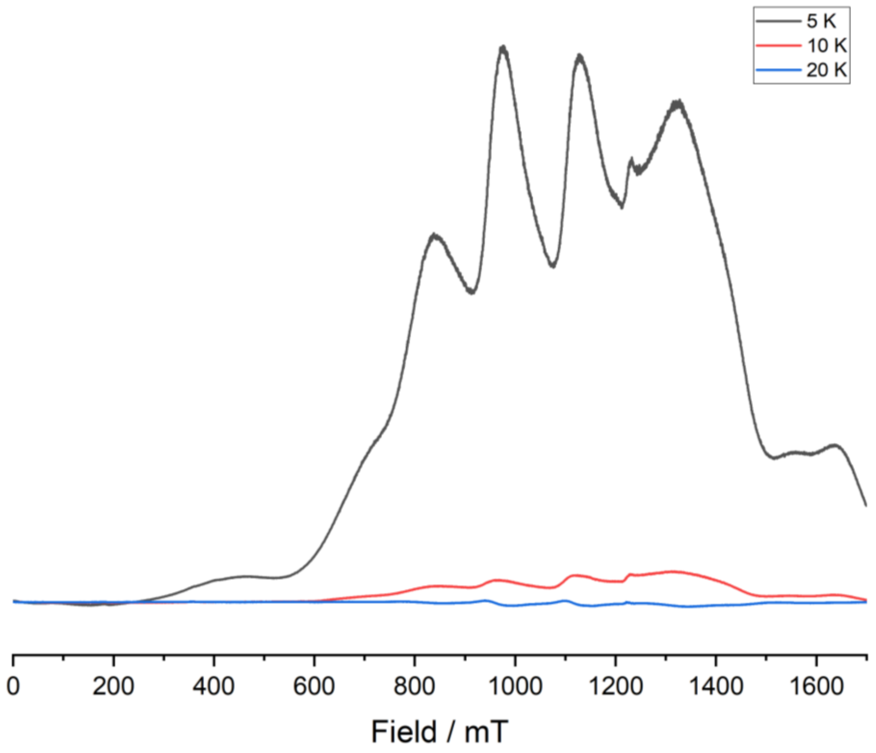

Figure S125. Overlaid frozen solution (10 mM) Q-band CW EPR spectra of **4-Eu** measured between 5-20 K.

10. *Ab initio* Calculations: 1-Ln, 3-Sm, 3-Eu, 4-Sm, 4-Eu

Table S6. Electronic structure of **1-Sm** calculated with the crystal field parameters obtained from CASSCF-SO using the solid-state geometry of **1-Sm** in zero-field. States are singly degenerate. [a] Contributions > 2%.

Energy (cm^{-1})	Energy (K)	Wavefunction[a]
0	0	98%|0⟩

Table S7. Electronic structure of **1-Eu** calculated with the crystal field parameters obtained from CASSCF-SO using the solid-state geometry of **1-Eu** in zero-field substituting Eu(1) for a Sr(II) ion. Each row corresponds to a Kramers doublet.
[a] Contributions > 2%.

Energy (cm^{-1})	Energy (K)	Wavefunction[a]	$\langle J_z \rangle$
0	0	39%|∓5/2⟩ + 31%|∓7/2⟩ + 20%|∓3/2⟩ + 7%|∓1/2⟩	±2.42
0.15	0.22	37%|∓7/2⟩ + 21%|∓3/2⟩ + 21%|∓1/2⟩ + 14%|±1/2⟩	±1.63
0.24	0.35	27%|∓1/2⟩ + 23%|±5/2⟩ + 21%|±7/2⟩ + 11%|∓3/2⟩ + 8%|±3/2⟩ + 7%|±1/2⟩	±1.14
0.33	0.47	33%|±5/2⟩ + 30%|±3/2⟩ + 15%|±1/2⟩ + 10%|±7/2⟩ + 7%|∓1/2⟩	±1.62

Table S8. Electronic structure of **1-Eu** calculated with the crystal field parameters obtained from CASSCF-SO using the solid-state geometry of **1-Eu** in zero-field substituting Eu(2) for a Sr(II) ion. Each row corresponds to a Kramers doublet.

[a] Contributions > 2%.

Energy (cm^{-1})	Energy (K)	Wavefunction[a]	$<J_z>$
0	0	$39\%|\pm 3/2\rangle + 36\%|\pm 5/2\rangle + 13\%|\pm 1/2\rangle + 11\%|\pm 7/2\rangle$	±1.95
0.12	0.17	$63\%|\pm 1/2\rangle + 16\%|\pm 5/2\rangle + 15\%|\pm 7/2\rangle$	±1.28
0.21	0.30	$47\%|\mp 3/2\rangle + 28\%|\mp 7/2\rangle + 19\%|\mp 1/2\rangle$	±1.89
0.36	0.52	$44\%|\pm 7/2\rangle + 43\%|\pm 5/2\rangle + 10\%|\pm 3/2\rangle$	±2.84

Table S9. Electronic structure of **3-Sm** calculated with the crystal field parameters obtained from CASSCF-SO using the solid-state geometry of **3-Sm** in zero-field. States are singly degenerate. [a] Contributions > 2%.

Energy (cm^{-1})	Energy (K)	Wavefunction[a]
0	0	$46\%|0\rangle$
523.45	753.13	$5\%|0\rangle$

Table S10. Electronic structure of **3-Eu** calculated with the crystal field parameters obtained from CASSCF-SO using the solid-state geometry of **3-Eu** in zero-field. Each row corresponds to a Kramers doublet. [a] Contributions > 2%.

Energy (cm^{-1})	Energy (K)	Wavefunction[a]	$\langle J_z \rangle$
0	0	44%$\|\pm 7/2\rangle$ + 29%$\|\pm 5/2\rangle$ + 16%$\|\pm 3/2\rangle$ + 7%$\|\pm 1/2\rangle$	±2.52
0.005	0.007	41%$\|\pm 7/2\rangle$ + 18%$\|\pm 1/2\rangle$ + 11%$\|\pm 3/2\rangle$ + 9%$\|\mp 1/2\rangle$ + 9%$\|\mp 3/2\rangle$ + 8%$\|\pm 5/2\rangle$	±1.59
0.009	0.013	27%$\|\mp 5/2\rangle$ + 26%$\|\pm 3/2\rangle$ + 16%$\|\pm 1/2\rangle$ + 15%$\|\pm 5/2\rangle$ + 12%$\|\mp 7/2\rangle$	±0.32
0.015	0.022	44%$\|\pm 1/2\rangle$ + 34%$\|\mp 3/2\rangle$ + 11%$\|\pm 5/2\rangle$ + 5%$\|\mp 5/2\rangle$	±0.17

Table S11. Electronic structure of **4-Sm** calculated with the crystal field parameters obtained from CASSCF-SO using the solid-state geometry of **4-Sm** in zero-field. States are singly degenerate. [a] Contributions > 2%.

Energy (cm^{-1})	Energy (K)	Wavefunction[a]
0	0	99%$\|0\rangle$

Table S12. Electronic structure of **4-Eu** calculated with the crystal field parameters obtained from CASSCF-SO using the solid-state geometry of **4-Eu** in zero-field. Each row corresponds to a Kramers doublet. [a] Contributions > 2%.

Energy (cm^{-1})	Energy (K)	Wavefunction[a]	$\langle J_z \rangle$
0	0	$33\%|\pm 7/2\rangle + 16\%|\pm 3/2\rangle + 13\%|\pm 5/2\rangle + 12\%|\pm 1/2\rangle +$ $11\%|\mp 1/2\rangle +$ $9\%|\mp 5/2\rangle +$ $5\%|\mp 3/2\rangle$	±1.45
0.116	0.173	$43\%|\pm 7/2\rangle + 24\%|\mp 5/2\rangle + 15\%|\mp 3/2\rangle + 8\%|\pm 1/2\rangle +$ $5\%|\mp 1/2\rangle$	±0.78
0.317	0.460	$20\%|\mp 5/2\rangle + 18\%|\pm 7/2\rangle + 14\%|\pm 3/2\rangle + 14\%|\pm 5/2\rangle +$ $13\%|\mp 3/2\rangle +$ $12\%|\mp 1/2\rangle +$ $6\%|\pm 1/2\rangle$	±0.46
0.619	0.892	$31\%|\mp 3/2\rangle + 25\%|\pm 1/2\rangle + 19\%|\mp 1/2\rangle + 13\%|\mp 5/2\rangle$	±0.78

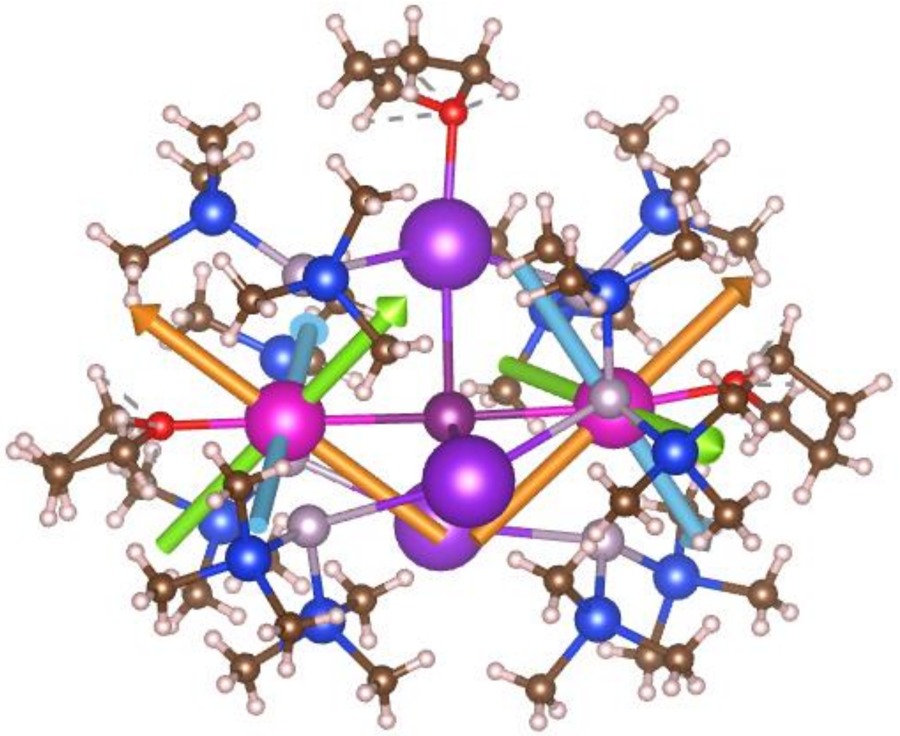

Figure S126. CASSCF-calculated magnetic axes for ground pseudo-doublet of **1-Eu** (green: g_1, orange: g_2, cyan: g_3). Europium, phosphorus, iodine, potassium, silicon, oxygen and carbon shown as magenta, gray, purple, mauve, blue, red, and brown, respectively.

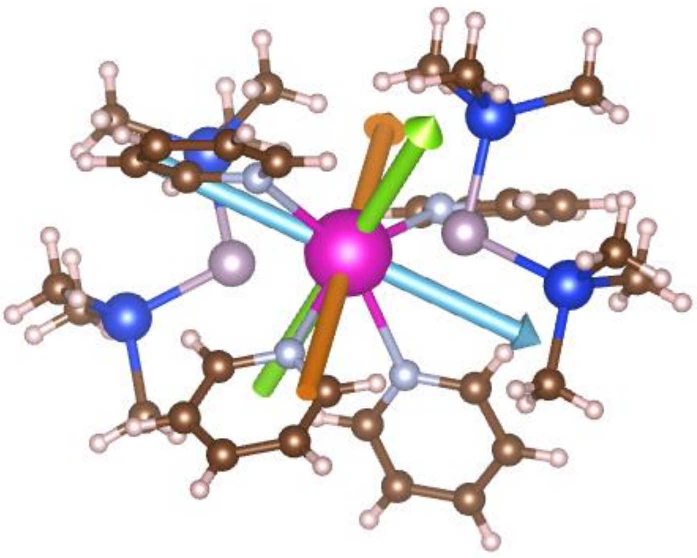

Figure S127. CASSCF-calculated magnetic axes for ground pseudo-doublet of **3-Eu** (green: g_1, orange: g_2, cyan: g_3). Europium, phosphorus, silicon, oxygen and carbon shown as magenta, gray, blue, red, and brown, respectively.

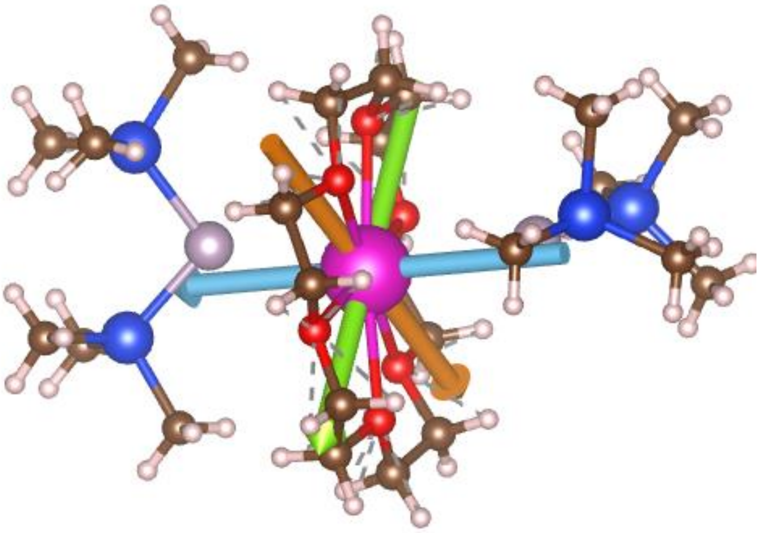

Figure S128. CASSCF-calculated magnetic axes for ground pseudo-doublet of **4-Eu** (green: g_1, orange: g_2, cyan: g_3). Europium, phosphorus, silicon, oxygen and carbon shown as magenta, gray, blue, red, and brown, respectively.

11. DFT Calculations: 3-Yb, 4-Yb

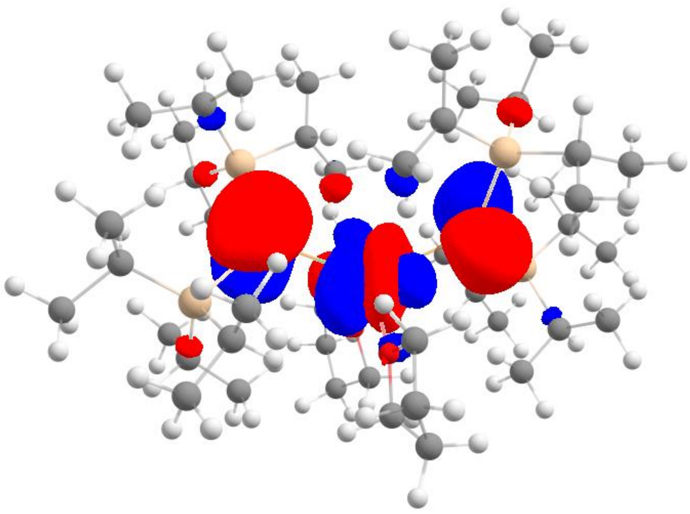

Figure S129. The highest occupied molecular orbital (HOMO) of **3-Yb** visualized at a 0.01 a.u. isosurface value.

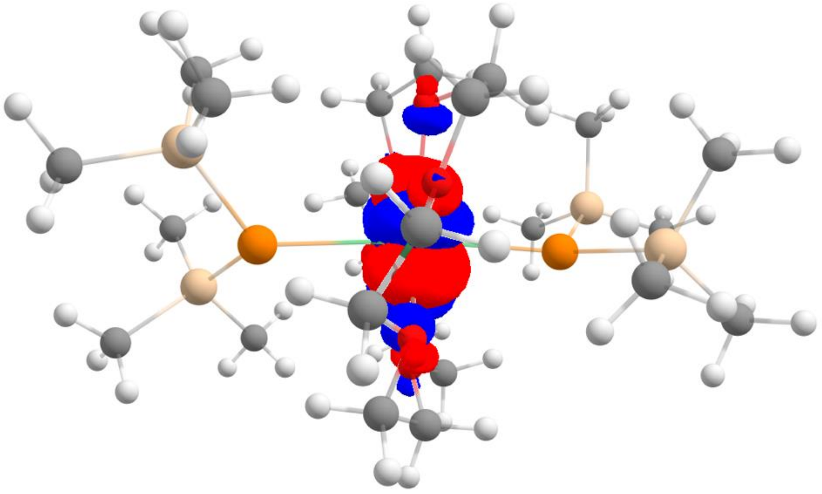

Figure S130. The highest occupied molecular orbital (HOMO) of **4-Yb** visualized at a 0.01 a.u. isosurface value.

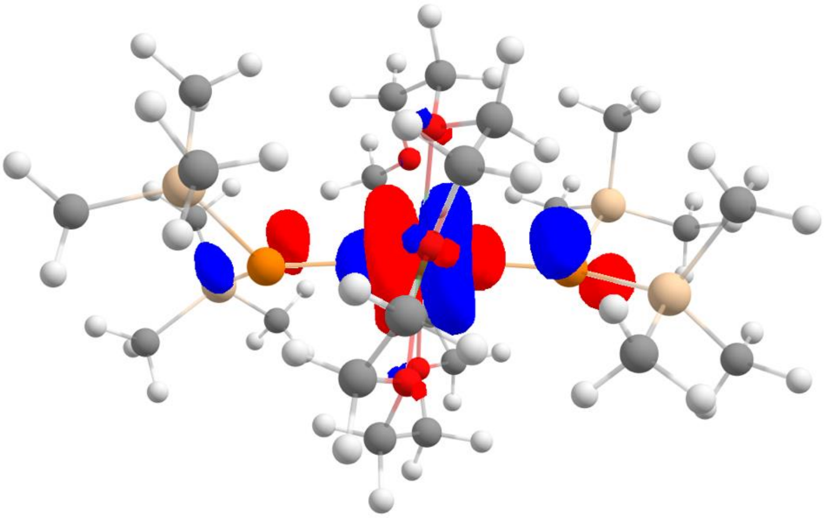

Figure S131. The second highest occupied molecular orbital (HOMO-1) of **4-Yb** visualized at a 0.01 a.u. isosurface value.
